# Supplementary material for: Guideline for reporting systematic reviews of outcome measurement instruments (OMIs): PRISMA-COSMIN for OMIs 2024
Source: J Patient Rep Outcomes. 2024 Jul 9;8:64. doi: 10.1186/s41687-024-00727-7 (PMC11231111; doi:10.1186/s41687-024-00727-7)
Supplement: Supplementary file 1 [file 41687_2024_727_MOESM1_ESM.pdf]

Guideline for reporting systematic reviews of outcome measurement instruments (OMIs): PRISMA-COSMIN for OMIs 2024

Ellen BM Elsman, Lidwine B Mokkink, Caroline B Terwee, Dorcas Beaton, Joel J Gagnier, Andrea C Tricco, Ami Baba, Nancy J Butcher, Maureen Smith, Catherine Hofstetter, Olalekan Lee Aiyegbusi, Anna Berardi, Julie Farmer, Kirstie L Haywood, Karolin R Krause, Sarah Markham, Evan Mayo-Wilson, Ava Mehdipour, Juanna Ricketts†, Peter Szatmari, Zahi Touma, David Moher, & Martin Offringa
martin.offringa@sickkids.ca

Online Resource 1. Deviations from the protocol [23] related to the development of PRISMA-COSMIN for OMIs 2024

- The intended response options for the Delphi study (definitely reject, probably reject, neutral, probably keep, definitely keep) were changed to strongly disagree, disagree, neutral, agree, strongly agree.
- In the protocol, we stated that only those items with a 70% reject vote would be excluded; all other items would move forward to the consensus meeting. This approach was not considered to be efficient. Therefore, we decided that consensus for inclusion was achieved when at least 67% of the panelists agreed or strongly agreed with a proposal and less than 15% disagreed or strongly disagreed. These items would not need further discussion at the workgroup meeting. Consensus for inclusion could only be reached starting from round 2 (i.e., after an item was evaluated at least two times), except for original and unmodified PRISMA 2020 items; their inclusion could be confirmed in round 1.
- Because we needed consensus on items that had no consensus yet after the Delphi study, we organized a hybrid workgroup meeting for which members of the steering committee and technical advisory group, as well as Delphi panelists (including patients/members of the public), knowledge users, and editors were invited, and in which consensus on those items was reached.
- To draft the guideline, instead of virtual workshops with members of the steering committee and technical advisory group, participants to the hybrid workgroup meeting were invited to draft the pre-final guideline.
- Instead of iteratively pilot testing each subsequent version of the guideline, starting after the Delphi study and continuing till after the consensus meeting, pilot testing took place after drafting the pre-final guideline, and only that version was pilot tested.
- The questions and response format used during pilot testing differed from those outlined in the protocol; the pilot testing form can be downloaded from https://osf.io/9fnj7/.
- Pilot testers were not recruited from specific disease areas. Instead, a broad sampling strategy was used to ensure coverage of various disease areas and types of OMI systematic reviews.
- The consensus meeting at the end of the project was renamed end-of-project meeting, as consensus on item inclusion was already obtained in an earlier stage and voting on items was no longer required. Members of the technical advisory group and Delphi panelists were not invited. Instead, pilot testers and experts in data visualization for systematic reviews of OMIs were present and the guideline was finalized based on results of pilot testing.
