# Supplementary material for: Guideline for reporting systematic reviews of outcome measurement instruments (OMIs): PRISMA-COSMIN for OMIs 2024
Source: J Patient Rep Outcomes. 2024 Jul 9;8:64. doi: 10.1186/s41687-024-00727-7 (PMC11231111; doi:10.1186/s41687-024-00727-7)
Supplement: Supplementary file 2 [file 41687_2024_727_MOESM2_ESM.pdf]

Guideline for reporting systematic reviews of outcome measurement instruments (OMIs): PRISMA-COSMIN for OMIs 2024

Ellen BM Elsman, Lidwine B Mokkink, Caroline B Terwee, Dorcas Beaton, Joel J Gagnier, Andrea C Tricco, Ami Baba, Nancy J Butcher, Maureen Smith, Catherine Hofstetter, Olalekan Lee Aiyegbusi, Anna Berardi, Julie Farmer, Kirstie L Haywood, Karolin R Krause, Sarah Markham, Evan Mayo-Wilson, Ava Mehdipour, Juanna Ricketts†, Peter Szatmari, Zahi Touma, David Moher, & Martin Offringa
martin.offringa@sickkids.ca

Online Resource 2. Group membership for the PRISMA-COSMIN for OMIs 2024 guideline

Name	Country	Group	Contribution
Ellen Elsman	Canada	Steering committee	Project lead; responsible for preparation, data collection, data analyses, dissemination of results
Wieneke Mokkink	Netherlands	Steering committee	Project oversight and supervision; workgroup meeting participant; drafted explanation & elaboration document
Caroline Terwee	Netherlands	Steering committee	Project oversight and supervision; workgroup meeting participant; drafted explanation & elaboration document
Dorcas Beaton	Canada	Steering committee	Delphi panelist; joined steering committee after Delphi study; workgroup meeting participant; drafted explanation & elaboration document
Joel Gagnier	Canada	Steering committee	Project oversight; workgroup meeting participant; drafted explanation & elaboration document
Andrea Tricco	Canada	Steering committee	Project oversight; workgroup meeting participant; drafted explanation & elaboration document
Nancy Butcher	Canada	Steering committee	Study conception; grant acquisition
Maureen Smith	Canada	Steering committee	Patient partner; project oversight; Delphi panelist; workgroup meeting participant; drafted explanation & elaboration document
David Moher	Canada	Steering committee	Grant acquisition; project oversight; workgroup meeting participant; drafted explanation & elaboration document
Martin Offringa	Canada	Steering committee	Study conception; grant acquisition; project oversight and supervision; workgroup meeting participant; drafted explanation & elaboration document
Ami Baba	Canada	Project manager	Project oversight and management
Olalekan Lee Aiyegbusi	UK	Technical advisory group	Delphi panelist; workgroup meeting participant; drafted explanation & elaboration document
Cornelia Borkhoff	Canada	Technical advisory group	Delphi panelist; workgroup meeting participant
Suneeta Monga	Canada	Technical advisory group	Grant acquisition
Karen Wong	Canada	Technical advisory group	Grant acquisition
Anne Klassen	Canada	Technical advisory group	Grant acquisition
Carolina Barnett-Tapia	Canada	Technical advisory group	Grant acquisition
Karolin Krause	Canada	Technical advisory group	Delphi panelist; workgroup meeting participant; drafted explanation & elaboration document

Elizabeth Potter	Canada	Technical advisory group	Delphi panelist; workgroup meeting participant
Andrea Monsour	Canada	Technical advisory group	Grant acquisition
Ava Mehdipour	Canada	Workgroup meeting participant	Delphi panelist; workgroup meeting participant; drafted explanation & elaboration document
Evan Mayo-Wilson	USA	Workgroup meeting participant	Delphi panelist; workgroup meeting participant; drafted explanation & elaboration document
Julie Farmer	Canada	Workgroup meeting participant	Delphi panelist; workgroup meeting participant; drafted explanation & elaboration document
Anna Berardi	Italy	Workgroup meeting participant	Delphi panelist; workgroup meeting participant; drafted explanation & elaboration document
Kirstie Haywood	UK	Workgroup meeting participant	Delphi panelist; workgroup meeting participant; drafted explanation & elaboration document
Zahi Touma	Canada	Workgroup meeting participant	Workgroup meeting participant; drafted explanation & elaboration document
Peter Szatmari	Canada	Workgroup meeting participant	Delphi panelist; workgroup meeting participant; drafted explanation & elaboration document
Kathleen Wyrwich	USA	Workgroup meeting participant	Delphi panelist; workgroup meeting participant
Sarah Markham	UK	Workgroup meeting participant	Patient/public contributor; Delphi panelist; workgroup meeting participant; drafted explanation & elaboration document
Juanna Ricketts†	Canada	Workgroup meeting participant	Patient/public contributor; Delphi panelist; workgroup meeting participant; drafted explanation & elaboration document
Catherine Hofstetter	Canada	Workgroup meeting participant	Patient/public contributor; Delphi panelist; accepted advisory role after Delphi study; workgroup meeting participant; drafted explanation & elaboration document

†Juanna Ricketts has been a valued patient contributor who recently passed away
