# Supplementary material for: Guideline for reporting systematic reviews of outcome measurement instruments (OMIs): PRISMA-COSMIN for OMIs 2024
Source: J Patient Rep Outcomes. 2024 Jul 9;8:64. doi: 10.1186/s41687-024-00727-7 (PMC11231111; doi:10.1186/s41687-024-00727-7)

Guideline for reporting systematic reviews of outcome measurement instruments (OMIs): PRISMA-COSMIN for OMIs 2024

Ellen BM Elsman, Lidwine B Mokkink, Caroline B Terwee, Dorcas Beaton, Joel J Gagnier, Andrea C Tricco, Ami Baba, Nancy J Butcher, Maureen Smith, Catherine Hofstetter, Olalekan Lee Aiyegbusi, Anna Berardi, Julie Farmer, Kirstie L Haywood, Karolin R Krause, Sarah Markham, Evan Mayo-Wilson, Ava Mehdipour, Juanna Ricketts†, Peter Szatmari, Zahi Touma, David Moher, & Martin Offringa
martin.offringa@sickkids.ca

Online Resource 3. Participant flow for the Delphi study (A) and pilot testing (B)

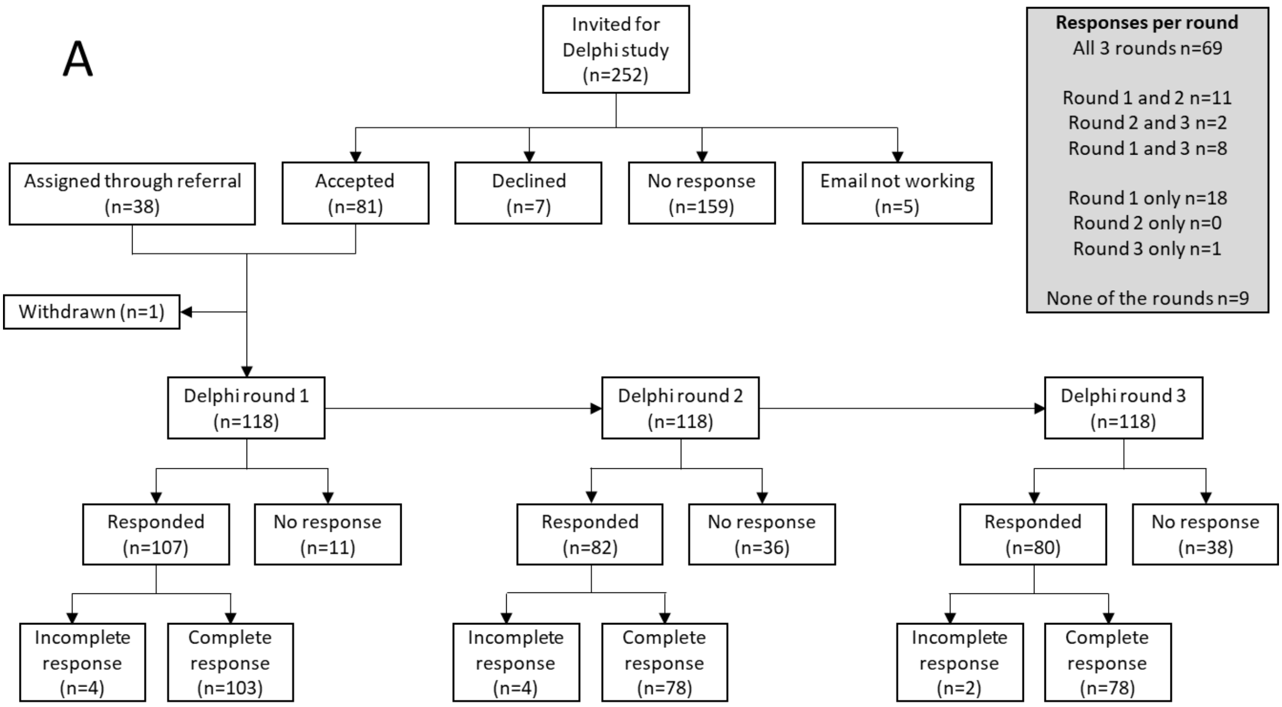

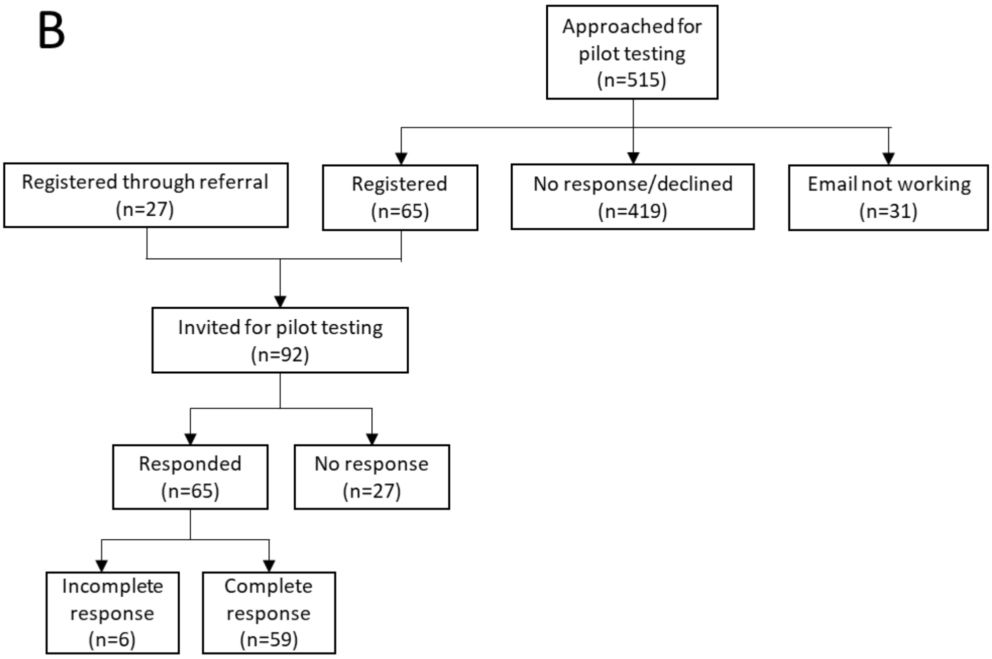

Supplement: Supplementary file 3 [file 41687_2024_727_MOESM3_ESM.pdf]
