# Supplementary material for: Guideline for reporting systematic reviews of outcome measurement instruments (OMIs): PRISMA-COSMIN for OMIs 2024
Source: J Patient Rep Outcomes. 2024 Jul 9;8:64. doi: 10.1186/s41687-024-00727-7 (PMC11231111; doi:10.1186/s41687-024-00727-7)
Supplement: Supplementary file 4 [file 41687_2024_727_MOESM4_ESM.pdf]

**Guideline for reporting systematic reviews of outcome measurement instruments (OMIs): PRISMA-COSMIN for OMIs 2024**

Ellen BM Elsman, Lidwine B Mokkink, Caroline B Terwee, Dorcas Beaton, Joel J Gagnier, Andrea C Tricco, Ami Baba, Nancy J Butcher, Maureen Smith, Catherine Hofstetter, Olalekan Lee Aiyegbusi, Anna Berardi, Julie Farmer, Kirstie L Haywood, Karolin R Krause, Sarah Markham, Evan Mayo-Wilson, Ava Mehdipour, Juanna Ricketts†, Peter Szatmari, Zahi Touma, David Moher, & Martin Offringa
martin.offringa@sickkids.ca

**Online Resource 4.** Changes in the reporting items over the Delphi rounds and workgroup meeting, and content comparisons to PRISMA 2020

| Section and topic | Item # | PRISMA 2020 item | Candidate PRISMA-COSMIN item Round 1 | Revised candidate PRISMA-COSMIN item Round 2 | Revised candidate PRISMA-COSMIN item Round 3 | Revised candidate PRISMA-COSMIN item Workgroup meeting |
|---|---|---|---|---|---|---|
| **TITLE** | | | | | | |
| Title | 1 | Identify the report as a systematic review. | Identify the report as a systematic review and include ~~the construct~~ of interest, population of interest, ~~(type of)~~ outcome measurement instrument(s) of interest, measurement property(ies) of interest.<br><br>**85% consensus for inclusion**<br>**73% consensus for wording** | Identify the report as a systematic review and include as applicable the following (in any order): outcome of interest, population of interest, name/type of outcome measurement instrument(s) of interest, and measurement property(ies) of interest.<br><br>**91% consensus for inclusion**<br>**86% consensus for wording** | *Consensus on inclusion obtained in Round 2.* | |
| **ABSTRACT** | | | | | | |
| Abstract | 2 | See the PRISMA ~~2020~~ for Abstracts checklist. | ~~Provide a structured summary, including objectives, eligibility criteria, information sources, risk of bias, synthesis of results, number of~~ included ~~articles, most important results, quality of~~ | See the PRISMA-COSMIN for Abstracts checklist. | See the PRISMA-COSMIN for Abstracts checklist. | See the PRISMA-COSMIN for Outcome Measurement Instruments Abstracts checklist. |

| Section and topic | Item # | PRISMA 2020 item | Candidate PRISMA-COSMIN item Round 1 | Revised candidate PRISMA-COSMIN item Round 2 | Revised candidate PRISMA-COSMIN item Round 3 | Revised candidate PRISMA-COSMIN item Workgroup meeting |
|---|---|---|---|---|---|---|
| | | | ~~evidence, interpretation, funding, registration.~~ ~~List keywords. If applicable, provide an executive/plain language summary with background information and main findings.~~<br><br>**79% consensus for inclusion**<br>**82% consensus for wording** | | | |
| **PRISMA-COSMIN for Abstracts checklist** | | | | | | |
| **TITLE** | | | | | | |
| Title | 2.1 | ~~Identify the report as a systematic review.~~ | | See item #1 of the PRISMA-COSMIN checklist.<br><br>**94% consensus for inclusion**<br>**92% consensus for wording** | *Consensus on inclusion obtained in Round 2.* | |
| **BACKGROUND** | | | | | | |
| Objectives | 2.2 | Provide an explicit statement of the main objective(s) or question(s) the review addresses. | | Provide an explicit statement of the main objective(s) or question(s) the review addresses.<br><br>**96% consensus for inclusion** | *Consensus on inclusion obtained in Round 2.* | |

| Section and topic | Item # | PRISMA 2020 item | Candidate PRISMA-COSMIN item Round 1 | Revised candidate PRISMA-COSMIN item Round 2 | Revised candidate PRISMA-COSMIN item Round 3 | Revised candidate PRISMA-COSMIN item Workgroup meeting |
|---|---|---|---|---|---|---|
| | | | | **97% consensus for wording** | | |
| **METHODS** | | | | | | |
| Eligibility criteria | 2.3 | Specify the inclusion and exclusion criteria for the review. | | Specify the inclusion and exclusion criteria for the review.<br><br>**96% consensus for inclusion**<br>**96% consensus for wording** | *Consensus on inclusion obtained in Round 2.* | |
| Information sources | 2.4 | Specify the information sources (e.g. databases, registers) used to identify studies and the date when each was last searched. | | Specify the information sources (e.g. databases, registers) used to identify studies and the date when each was last searched.<br><br>**92% consensus for inclusion**<br>**96% consensus for wording** | *Consensus on inclusion obtained in Round 2.* | |
| Risk of bias | 2.5 | Specify the methods used to assess risk of bias in the included studies. | | Specify the methods used to assess risk of bias in the included studies.<br><br>**88% consensus for inclusion**<br>**92% consensus for wording** | *Consensus on inclusion obtained in Round 2.* | |

| Section and topic | Item # | PRISMA 2020 item | Candidate PRISMA-COSMIN item Round 1 | Revised candidate PRISMA-COSMIN item Round 2 | Revised candidate PRISMA-COSMIN item Round 3 | Revised candidate PRISMA-COSMIN item Workgroup meeting |
|---|---|---|---|---|---|---|
| Synthesis of results | 2.6 | Specify the methods used to present and synthesize results. | | Specify the methods used to present and synthesize results.<br><br>**87% consensus for inclusion**<br>**92% consensus for wording** | *Consensus on inclusion obtained in Round 2.* | |
| **RESULTS** | | | | | | |
| Included studies | 2.7 | Give the total number of included ~~studies~~ and ~~participants~~ and summarize relevant characteristics of ~~studies.~~ | | Give the total number of included outcome measurement instruments and reports.<br><br>**95% consensus for inclusion**<br>**87% consensus for wording** | Give the total number of included outcome measurement instruments and reports.<br><br>**98% consensus for inclusion**<br>**91% consensus for wording** | *Consensus on inclusion obtained in Round 3.* |
| Synthesis of results | 2.8 | Present results ~~for main outcomes, preferably~~ indicating the ~~number of included studies and participants for each. If meta-analysis was done, report the summary estimate and confidence/credible interval. If comparing groups, indicate the direction of the effect~~ | | Present results for measurement properties of outcome measurement instruments, ~~preferably~~ indicating the certainty of the evidence.<br><br>**91% consensus for inclusion**<br>**83% consensus for wording** | Present ~~main~~ results ~~for measurement properties~~ of outcome measurement instruments, indicating the certainty of the evidence.<br><br>**91% consensus for inclusion**<br>**90% consensus for wording** | **Present the syntheses of results of outcome measurement instruments, indicating the certainty of the evidence.** |

| Section and topic | Item # | PRISMA 2020 item | Candidate PRISMA-COSMIN item Round 1 | Revised candidate PRISMA-COSMIN item Round 2 | Revised candidate PRISMA-COSMIN item Round 3 | Revised candidate PRISMA-COSMIN item Workgroup meeting |
|---|---|---|---|---|---|---|
| | | ~~(i.e. which group is favored).~~ | | | | |
| **DISCUSSION** | | | | | | |
| Limitations of evidence | 2.9 | Provide a brief summary of the limitations of the evidence included in the review (e.g. study risk of bias, inconsistency and imprecision). | | Provide a brief summary of the limitations of the evidence included in the review (e.g. study risk of bias, inconsistency and imprecision).<br><br>**92% consensus for inclusion**<br>**92% consensus for wording** | *Consensus on inclusion obtained in Round 2.* | |
| Interpretation | 2.10 | Provide a general interpretation of the results and important implications. | | Provide a general interpretation of the results and important implications.<br><br>**100% consensus for inclusion**<br>**97% consensus for wording** | *Consensus on inclusion obtained in Round 2.* | |
| **OTHER** | | | | | | |
| Funding | 2.11 | Specify the primary source of funding for the review. | | Specify the primary source of funding for the review.<br><br>**92% consensus for inclusion**<br>**96% consensus for wording** | *Consensus on inclusion obtained in Round 2.* | |

| Section and topic | Item # | PRISMA 2020 item | Candidate PRISMA-COSMIN item Round 1 | Revised candidate PRISMA-COSMIN item Round 2 | Revised candidate PRISMA-COSMIN item Round 3 | Revised candidate PRISMA-COSMIN item Workgroup meeting |
|---|---|---|---|---|---|---|
| Registration | 2.12 | Provide the register name and registration number. | | Provide the register name and registration number.<br><br>**90% consensus for inclusion**<br>**91% consensus for wording** | *Consensus on inclusion obtained in Round 2.* | |
| **PLAIN LANGUAGE SUMMARY** | | | | | | |
| Plain language summary | 2.13 | | If allowed by the journal, provide a plain language summary with background information and ~~main~~ findings.<br><br>**71% consensus for inclusion**<br>**82% consensus for wording** | If allowed by the journal, provide a plain language summary with background information and key findings.<br><br>**90% consensus for inclusion**<br>**91% consensus for wording** | *Consensus on inclusion obtained in Round 3.* | Plain language summary |
| **INTRODUCTION** | | | | | | |
| Rationale | 3 | Describe the rationale for the review in the context of existing knowledge. | Describe the rationale for the review in the context of existing knowledge.<br><br>**98% consensus for inclusion**<br>**98% consensus for wording** | *Consensus on inclusion obtained in Round 1.* | | |
| Name and description of outcome | PC1 | | *~~[If only one or few outcome measurement instrument(s) are subject~~* | *Not applicable – This item is merged into the* | *Consensus on deletion obtained in Round 2: Item deleted.* | |

| Section and topic | Item # | PRISMA 2020 item | Candidate PRISMA-COSMIN item Round 1 | Revised candidate PRISMA-COSMIN item Round 2 | Revised candidate PRISMA-COSMIN item Round 3 | Revised candidate PRISMA-COSMIN item Workgroup meeting |
|---|---|---|---|---|---|---|
| measurement instrument(s) of interest | | | ~~*of research:]* Specify the name(s) of the outcome measurement instrument(s) of interest with a citation, and how they were developed, their construct, intended use, and target population.~~<br><br>**91% consensus for inclusion**<br>**76% consensus for wording** | *background information of item #3*<br><br>**94% consensus for deletion** | | |
| Objectives | 4 | Provide an explicit statement of the objective(s) or question(s) the review addresses. | Provide an explicit statement of the objective(s) or question(s) the review addresses ~~including the construct~~ of interest, population of interest, ~~(type of)~~ outcome measurement instrument(s) of interest, measurement property(ies) of interest~~, as well as feasibility and interpretability aspects, if part of the objectives of the review~~.<br><br>**91% consensus for inclusion** | Provide an explicit statement of the objective(s) or question(s) the review addresses and include as applicable the following (in any order): outcome of interest, population of interest, name/type of outcome measurement instrument(s) of interest, and measurement property(ies) of interest.<br><br>**97% consensus for inclusion**<br>**88% consensus for wording** | *Consensus on inclusion obtained in Round 2.* | |

| Section and topic | Item # | PRISMA 2020 item | Candidate PRISMA-COSMIN item Round 1 | Revised candidate PRISMA-COSMIN item Round 2 | Revised candidate PRISMA-COSMIN item Round 3 | Revised candidate PRISMA-COSMIN item Workgroup meeting |
|---|---|---|---|---|---|---|
| | | | **82% consensus for wording** | | | |
| **METHODS** | | | | | | |
| Followed guidelines | PC2 | | Specify the methodology or guidelines used to conduct and report the systematic review ~~with citations~~.<br><br>**93% consensus for inclusion**<br>**92% consensus for wording** | Specify, with citations, the methodology and/or guidelines used to conduct ~~and report~~ the systematic review.<br><br>**92% consensus for inclusion**<br>**95% consensus for wording** | Specify, with citations, the methodology and/or guidelines used to conduct the systematic review.<br><br>**98% consensus for inclusion**<br>**98% consensus for wording** | *Consensus on inclusion obtained in Round 3.* |
| Eligibility criteria | 5 | Specify the inclusion and exclusion criteria for the review ~~and how studies were grouped for the syntheses~~. | Specify the inclusion and exclusion criteria for the review.<br><br>**92% consensus for inclusion**<br>**92% consensus for wording** | *Consensus on inclusion obtained in Round 1.* | | |
| Information sources | 6 | Specify all databases, registers, websites, organisations, reference lists and other sources searched or consulted to identify studies. Specify the date when each source was last searched or consulted. | Specify all databases, registers, websites, organisations, reference lists and other sources searched or consulted to identify studies ~~and articles, outcome measurement instrument(s), user/scoring manuals, and/or measurement protocols~~. Specify the date when each source | Specify all databases, registers, websites, organisations, reference lists and other sources searched or consulted to identify studies. Specify the date when each source was last searched or consulted.<br><br>**97% consensus for inclusion** | *Consensus on inclusion obtained in Round 2.* | |

| Section and topic | Item # | PRISMA 2020 item | Candidate PRISMA-COSMIN item Round 1 | Revised candidate PRISMA-COSMIN item Round 2 | Revised candidate PRISMA-COSMIN item Round 3 | Revised candidate PRISMA-COSMIN item Workgroup meeting |
|---|---|---|---|---|---|---|
| | | | was last searched or consulted.<br><br>**83% consensus for inclusion**<br>**85% consensus for wording** | **96% consensus for wording** | | |
| Search strategy | 7 | Present the full search strategies for all databases, registers, and websites, including any filters and limits used. | Present the full search strategies for all databases, registers, and websites, including any filters and limits used.<br><br>**99% consensus for inclusion**<br>**96% consensus for wording** | *Consensus on inclusion obtained in Round 1.* | | |
| Selection process | 8 | Specify the methods used to decide whether a study met the inclusion criteria of the review, including how many reviewers screened each record and each report retrieved, whether they worked independently, and if applicable, details of automation tools used in the process. | Specify the methods used to decide whether a study met the inclusion criteria of the review, including how many reviewers screened each record and each report retrieved, whether they worked independently and how discrepancies were resolved, and if applicable, details of automation tools used in the process. | Specify the methods used to decide whether a study met the inclusion criteria of the review, including how many reviewers screened each record and each report retrieved, whether they worked independently ~~and, if so, how discrepancies were resolved~~, and if applicable, details of automation tools used in the process. | Specify the methods used to decide whether a study met the inclusion criteria of the review, including how many reviewers screened each record and each report retrieved, whether they worked independently, and if applicable, details of automation tools used in the process.<br><br>**94% consensus for inclusion**<br>**94% consensus for wording** | *Consensus on inclusion obtained in Round 3.* |

| Section and topic | Item # | PRISMA 2020 item | Candidate PRISMA-COSMIN item Round 1 | Revised candidate PRISMA-COSMIN item Round 2 | Revised candidate PRISMA-COSMIN item Round 3 | Revised candidate PRISMA-COSMIN item Workgroup meeting |
|---|---|---|---|---|---|---|
| | | | **97% consensus for inclusion**<br>**94% consensus for wording** | **97% consensus for inclusion**<br>**91% consensus for wording** | | |
| Data collection process | 9 | Specify the methods used to collect data from reports, including how many reviewers collected data from each report, whether they worked independently, any processes for obtaining or confirming data from study investigators, and if applicable, details of automation tools used in the process. | Specify the methods used to collect data from reports, including how many reviewers collected data from each report, whether they worked independently and how discrepancies were resolved, any processes for obtaining or confirming data from study investigators, and if applicable, details of automation tools used in the process.<br><br>**98% consensus for inclusion**<br>**92% consensus for wording** | Specify the methods used to collect data from reports, including how many reviewers collected data from each report, whether they worked independently ~~and, if so, how discrepancies were resolved~~, any processes for obtaining or confirming data from study investigators, and if applicable, details of automation tools used in the process.<br><br>**97% consensus for inclusion**<br>**95% consensus for wording** | Specify the methods used to collect data from reports, including how many reviewers collected data from each report, whether they worked independently any processes for obtaining or confirming data from study investigators, and if applicable, details of automation tools used in the process.<br><br>**97% consensus for inclusion**<br>**90% consensus for wording** | *Consensus on inclusion obtained in Round 3.* |
| Data items | 10a | ~~List and define all outcomes for which data were sought. Specify whether all results that were compatible with each outcome domain in each study were sought (e.g. for all measures, time points, analyses),~~ | *Not applicable – item #10b is a better fit to the data extracted in systematic reviews of outcome measurement instruments*<br><br>**93% consensus for deletion** | *Consensus on deletion obtained in Round 1: Item deleted.* | | |

| Section and topic | Item # | PRISMA 2020 item | Candidate PRISMA-COSMIN item Round 1 | Revised candidate PRISMA-COSMIN item Round 2 | Revised candidate PRISMA-COSMIN item Round 3 | Revised candidate PRISMA-COSMIN item Workgroup meeting |
|---|---|---|---|---|---|---|
| | | ~~and if not, the methods used to decide which results to collect.~~ | | | | |
| | 10b | List and define ~~all other variables for~~ which data were ~~sought~~ (e.g. ~~participant and intervention~~ characteristics, ~~funding sources~~). Describe any assumptions made about any missing or unclear information. | List and define which data were extracted (e.g. study population~~,~~ ~~intervention~~ and outcome measurement instrument(s) characteristics, ~~funding sources,~~ ~~results of studies on~~ measurement properties, feasibility and interpretability aspects). Describe any assumptions made about any missing or unclear information.<br><br>**93% consensus for inclusion**<br>**87% consensus for wording** | List and define which data were extracted (e.g. study population and outcome measurement instrument(s) characteristics, measurement property(ies) results, and feasibility and interpretability aspects). Describe any assumptions made about any missing or unclear information.<br><br>**92% consensus for inclusion**<br>**87% consensus for wording** | List and define which data were extracted (e.g. characteristics of study population(s) and outcome measurement instrument(s), results of measurement property(ies), and aspects of feasibility and interpretability). Describe any assumptions made about any missing or unclear information.<br><br>**99% consensus for inclusion**<br>**91% consensus for wording** | *Consensus on inclusion obtained in Round 3.* |
| Study risk of bias assessment | 11 | Specify the methods used to assess risk of bias in the included studies, including details of the tool(s) used, how many reviewers assessed each study and whether they worked independently, and if applicable, details of | Specify the methods used to assess risk of bias in the included studies, including details of the tool(s) used, how many reviewers assessed each study, ~~their expertise,~~ whether they worked independently and how discrepancies were | Specify the methods used to assess risk of bias in the included studies, including details of the tool(s) used, how many reviewers assessed each study, whether they worked independently ~~and, if so, how discrepancies were resolved~~, and if | Specify the methods used to assess risk of bias in the included studies, including details of the tool(s) used, how many reviewers assessed each study and whether they worked independently, and if applicable, details of | *Consensus on inclusion obtained in Round 3.* |

| Section and topic | Item # | PRISMA 2020 item | Candidate PRISMA-COSMIN item Round 1 | Revised candidate PRISMA-COSMIN item Round 2 | Revised candidate PRISMA-COSMIN item Round 3 | Revised candidate PRISMA-COSMIN item Workgroup meeting |
|---|---|---|---|---|---|---|
| | | automation tools used in the process. | resolved, and if applicable, details of automation tools used in the process.<br>**77% consensus for inclusion**<br>**87% consensus for wording** | applicable, details of automation tools used in the process.<br>**91% consensus for inclusion**<br>**91% consensus for wording** | automation tools used in the process.<br>**87% consensus for inclusion**<br>**91% consensus for wording** | |
| ~~Criteria for good~~ Measurement properties | PC3 | | Specify the methods used to rate the results ~~of each individual study~~ and the summarized or pooled results. Specify how many reviewers evaluated each study, ~~their expertise,~~ whether they worked independently and how discrepancies were resolved.<br>*[For construct validity, responsiveness:]* Specify the hypotheses formulated.<br>*[For criterion validity:]* Specify which outcome measurement instrument is considered to be a gold standard.<br>**81% consensus for inclusion** | ~~*[For all measurement properties:]*~~ Specify the methods used to rate the results ~~on~~ a measurement property for each individual study and the summarized or pooled results ~~on a measurement property for each outcome measurement instrument~~. Specify how many reviewers ~~evaluated~~ each study, whether they worked independently ~~and, if so, how discrepancies were resolved.~~ ~~*[For construct validity, responsiveness:]*~~ ~~Specify the hypotheses formulated by the reviewers.~~ ~~*[For criterion validity:]*~~ ~~Specify which outcome measurement~~ | Specify the methods used to rate the results of a measurement property for each individual study and for the summarized or pooled results. Specify how many reviewers rated each study and whether they worked independently.<br>**93% consensus for inclusion**<br>**90% consensus for wording** | *Consensus on inclusion obtained in Round 3.* |

| Section and topic | Item # | PRISMA 2020 item | Candidate PRISMA-COSMIN item Round 1 | Revised candidate PRISMA-COSMIN item Round 2 | Revised candidate PRISMA-COSMIN item Round 3 | Revised candidate PRISMA-COSMIN item Workgroup meeting |
|---|---|---|---|---|---|---|
| | | | **70% consensus for wording** | ~~instrument is considered to be a gold standard.~~<br><br>**86% consensus for inclusion**<br>**75% consensus for wording** | | |
| Effect measures | 12 | ~~Specify for each outcome the effect measure(s) (e.g. risk ratio, mean difference) used in the synthesis or presentation of results.~~ | *Not applicable – effect measures is not applicable to systematic reviews of outcome measurement instruments; the item is replaced by item PC3*<br><br>**93% consensus for deletion** | *Consensus on deletion obtained in Round 1: Item deleted.* | | |
| Synthesis methods | 13a | Describe the processes used to decide which studies were eligible for each synthesis ~~(e.g. tabulating the study intervention characteristics and comparing against the planned groups for each synthesis (item #5)).~~ | Describe the processes used to decide which studies were eligible for ~~summarizing or pooling the results.~~<br>~~*[For content validity:]*~~ ~~Describe the processes used to decide which results can be extrapolated from one instrument to other instruments.~~<br><br>**82% consensus for inclusion**<br>**66% consensus for wording** | Describe the processes used to decide which studies ~~per measurement property~~ were eligible for each synthesis.<br><br>**89% consensus for inclusion**<br>**82% consensus for wording** | Describe the processes used to decide which studies were eligible for each synthesis ~~of each measurement property~~.<br><br>**97% consensus for inclusion**<br>**90% consensus for wording** | Describe the processes used to decide which studies were eligible for each synthesis. |

| Section and topic | Item # | PRISMA 2020 item | Candidate PRISMA-COSMIN item Round 1 | Revised candidate PRISMA-COSMIN item Round 2 | Revised candidate PRISMA-COSMIN item Round 3 | Revised candidate PRISMA-COSMIN item Workgroup meeting |
|---|---|---|---|---|---|---|
| | 13b | ~~Describe any methods required to prepare the data for presentation or synthesis, such as handling of missing summary statistics, or data conversions.~~ | ~~Describe any methods required to prepare the data for presentation or synthesis, such as handling of missing results, or data conversions.~~<br><br>**93% consensus for inclusion**<br>**90% consensus for wording** | *Not applicable – This item is merged into the background information of item #10b and item #13e*<br><br>**94% consensus for deletion** | *Consensus on deletion obtained in Round 2: Item deleted.* | |
| | 13c | ~~Describe any methods used to tabulate or visually display results of individual studies and syntheses.~~ | ~~Describe any methods used to tabulate or visually display results of individual studies and syntheses.~~<br><br>**86% consensus for inclusion**<br>**91% consensus for wording** | *Not applicable – Results are mostly tabulated and text in the methods on how this was done is not required*<br><br>**91% consensus for deletion** | *Consensus on deletion obtained in Round 2: Item deleted.* | |
| | 13d | Describe any methods used to synthesize results and provide a rationale for the choice(s). ~~If meta-analysis was performed, describe the model(s), method(s) to identify the presence and extent of statistical heterogeneity, and~~ | Describe any methods used to synthesize results and provide a rationale for the choice(s). ~~If meta-analysis was performed, describe the model(s), method(s) to identify the presence and extent of statistical heterogeneity, and~~ | Describe any methods used to synthesize results and provide a rationale for the choice(s).<br><br>**96% consensus for inclusion**<br>**98% consensus for wording** | *Consensus on inclusion obtained in Round 2.* | |

| Section and topic | Item # | PRISMA 2020 item | Candidate PRISMA-COSMIN item Round 1 | Revised candidate PRISMA-COSMIN item Round 2 | Revised candidate PRISMA-COSMIN item Round 3 | Revised candidate PRISMA-COSMIN item Workgroup meeting |
|---|---|---|---|---|---|---|
| | | ~~software package(s) used.~~ | ~~software package(s) used.~~<br><br>**91% consensus for inclusion**<br>**94% consensus for wording** | | | |
| | 13e | Describe any methods used to explore possible causes of ~~heterogeneity~~ among study results (e.g. subgroup analysis~~,~~ ~~meta-regression~~). | Describe any methods used to explore possible causes of inconsistency among study results.<br><br>**90% consensus for inclusion**<br>**87% consensus for wording** | Describe any methods used to explore possible causes of inconsistency among study results (e.g. subgroup analysis).<br><br>**94% consensus for inclusion**<br>**88% consensus for wording** | *Consensus on inclusion obtained in Round 2.* | |
| | 13f | Describe any sensitivity analyses conducted to assess robustness of the synthesized results. | Describe any ~~subgroup~~ analyses ~~if~~ conducted.<br><br>**88% consensus for inclusion**<br>**89% consensus for wording** | Describe any sensitivity analyses conducted to assess robustness of the synthesized results.<br><br>**79% consensus for inclusion**<br>**78% consensus for wording** | If applicable, describe any sensitivity analyses conducted to assess robustness of the synthesized results.<br><br>**91% consensus for inclusion**<br>**94% consensus for wording** | *Consensus on inclusion obtained in Round 3.* |
| Reporting bias assessment | 14 | ~~Describe any methods used to assess risk of bias due to missing results in a synthesis (arising from reporting biases).~~ | *Not applicable – it is impossible to know whether reporting/publication bias exists and hence this cannot be assessed* | *Consensus on deletion obtained in Round 1: Item deleted.* | | |

| Section and topic | Item # | PRISMA 2020 item | Candidate PRISMA-COSMIN item Round 1 | Revised candidate PRISMA-COSMIN item Round 2 | Revised candidate PRISMA-COSMIN item Round 3 | Revised candidate PRISMA-COSMIN item Workgroup meeting |
|---|---|---|---|---|---|---|
| | | | **81% consensus for deletion** | | | |
| Certainty assessment | 15 | Describe any methods used to assess certainty (or confidence) in the body of evidence ~~for an outcome~~. | Describe any methods used to assess certainty (or confidence) in the body of evidence for each measurement property for each outcome measurement instrument. Describe how many reviewers graded the evidence, ~~their expertise,~~ whether they worked independently and how discrepancies were resolved.<br><br>**83% consensus for inclusion**<br>**77% consensus for wording** | Describe any methods used to assess certainty (or confidence) in the body of evidence for each measurement property ~~for~~ each outcome measurement instrument. ~~Describe how many reviewers graded the evidence, whether they worked independently and, if so, how discrepancies were resolved.~~<br><br>**91% consensus for inclusion**<br>**87% consensus for wording** | Describe any methods used to assess certainty (or confidence) in the body of evidence ~~for each measurement property of each outcome measurement instrument~~.<br><br>**95% consensus for inclusion**<br>**91% consensus for wording** | Describe any methods used to assess certainty (or confidence) in the body of evidence. |
| Formulating recommendations | PC4 | | Describe any methods used to formulate recommendations and provide a rationale for the choice(s).<br><br>**79% consensus for inclusion**<br>**83% consensus for wording** | Describe any methods used to formulate recommendations ~~on the most~~ suitable outcome measurement instrument for a ~~given purpose and provide a rationale for the choice(s)~~.<br><br>**85% consensus for inclusion** | If ~~applicable~~, describe any methods used to formulate recommendations regarding ~~suitable~~ outcome measurement instruments for a particular use.<br><br>**91% consensus for inclusion** | If appropriate, describe any methods used to formulate recommendations regarding the suitability of outcome measurement instruments for a particular use. |

| Section and topic | Item # | PRISMA 2020 item | Candidate PRISMA-COSMIN item Round 1 | Revised candidate PRISMA-COSMIN item Round 2 | Revised candidate PRISMA-COSMIN item Round 3 | Revised candidate PRISMA-COSMIN item Workgroup meeting |
|---|---|---|---|---|---|---|
| | | | | **87% consensus for wording** | **91% consensus for wording** | |
| **RESULTS** | | | | | | |
| Study selection | 16a | Describe the results of the search and selection process, from the number of records identified in the search to the number of ~~studies~~ included in the review, ideally using a flow diagram. | Describe the results of the search and selection process, from the number of records identified in the search to the number of ~~articles~~ included in the review, ideally using a flow diagram. ~~Describe~~ the final number of outcome measurement instrument(s) included.<br><br>**95% consensus for inclusion**<br>**90% consensus for wording** | Describe the results of the search and selection process, from the number of records identified in the search to the number of ~~records~~ included in the review, ideally using a flow diagram. If applicable, also report the final number of outcome measurement instrument(s) included.<br><br>**96% consensus for inclusion**<br>**91% consensus for wording** | Describe the results of the search and selection process, from the number of records identified in the search to the number of reports included in the review, ideally using a flow diagram. If applicable, also report the final number of outcome measurement instrument(s) included.<br><br>**99% consensus for inclusion**<br>**90% consensus for wording** | Describe the results of the search and selection process, from the number of records identified in the search to the number of reports included in the review, ideally using a flow diagram. If applicable, also report the final number of outcome measurement instrument(s) included and the number of reports relevant to each instrument. |
| | 16b | Cite ~~studies~~ that might appear to meet the inclusion criteria, but which were excluded, and explain why they were excluded. | ~~Cite articles that might appear to meet the inclusion criteria, but which were excluded, and explain why they were excluded.~~<br><br>**85% consensus for inclusion**<br>**88% consensus for wording** | *Not applicable – Will be clear from the flow diagram*<br><br>**90% consensus for deletion** | Cite reports that might appear to meet the inclusion criteria, but which were excluded, and explain why they were excluded.<br><br>**71% consensus for inclusion**<br>**81% consensus for wording** | *Consensus on inclusion obtained in workgroup meeting.* |
| Outcome measurement | PC5 | | ~~*[If multiple outcome measurement*~~ | ~~Cite the first report on each included outcome~~ | Present characteristics of each included | *Consensus on inclusion obtained in Round 3.* |

| Section and topic | Item # | PRISMA 2020 item | Candidate PRISMA-COSMIN item Round 1 | Revised candidate PRISMA-COSMIN item Round 2 | Revised candidate PRISMA-COSMIN item Round 3 | Revised candidate PRISMA-COSMIN item Workgroup meeting |
|---|---|---|---|---|---|---|
| instrument characteristics | | | *~~instruments are included:]~~* Cite each included outcome measurement instrument and present outcome measurement instrument(s) ~~characteristics~~.<br><br>**93% consensus for inclusion**<br>**81% consensus for wording** | ~~measurement instrument and~~ present characteristics of ~~the~~ outcome measurement instrument~~(s)~~.<br><br>**87% consensus for inclusion**<br>**83% consensus for wording** | outcome measurement instrument, with appropriate citations.<br><br>**97% consensus for inclusion**<br>**96% consensus for wording** | |
| Study characteristics | 17 | Cite each included ~~study~~ and present its characteristics. | Cite each included ~~article on a~~ measurement property and present ~~its~~ population characteristics.<br><br>**90% consensus for inclusion**<br>**83% consensus for wording** | Cite each included report evaluating one or more measurement property(ies) and present sample characteristics ~~for each measurement property evaluated~~.<br><br>**96% consensus for inclusion**<br>**90% consensus for wording** | Cite each included report evaluating one or more measurement property(ies) and present sample characteristics.<br><br>**99% consensus for inclusion**<br>**95% consensus for wording** | *Consensus on inclusion obtained in Round 3.* |
| Risk of bias in studies | 18 | Present assessments of risk of bias for each included study. | Present ~~consensus ratings on~~ risk of bias ~~per outcome measurement instrument development and measurement property (i.e. COSMIN~~ | Present assessments of risk of bias for each included study.<br><br>**97% consensus for inclusion**<br>**96% consensus for wording** | *Consensus on inclusion obtained in Round 2.* | |

| Section and topic | Item # | PRISMA 2020 item | Candidate PRISMA-COSMIN item Round 1 | Revised candidate PRISMA-COSMIN item Round 2 | Revised candidate PRISMA-COSMIN item Round 3 | Revised candidate PRISMA-COSMIN item Workgroup meeting |
|---|---|---|---|---|---|---|
| | | | ~~box 1-10)~~ for each included study.<br><br>**86% consensus for inclusion**<br>**76% consensus for wording** | | | |
| Results of individual studies | 19 | For all ~~outcomes~~, present, for each study: (a) ~~summary statistics for each group (where appropriate)~~ and (b) ~~an effect estimate and its precision (e.g. confidence/credible interval),~~ ideally using structured tables or plots. | For all measurement properties, present, for each study: (a) the result and (b) the rating ~~(i.e. against the COSMIN Criteria for Good Measurement Properties)~~, ideally using structured tables or plots.<br><br>**91% consensus for inclusion**<br>**86% consensus for wording** | For all measurement properties, present, for each study: (a) the reported result and (b) the rating against quality criteria, ideally using structured tables or plots.<br><br>**97% consensus for inclusion**<br>**94% consensus for wording** | *Consensus on inclusion obtained in Round 2.* | |
| Results of syntheses | 20a | ~~For each synthesis, briefly summarise the characteristics and risk of bias among contributing studies.~~ | ~~*[For content validity:]* Present, for each outcome measurement instrument, the overall relevance, comprehensiveness, comprehensibility, and optionally content validity ratings.~~<br>~~*[For all other measurement properties:]* Present, for~~ | *Not applicable – The high number of syntheses usually conducted in systematic reviews of outcome measurement instruments make that requiring this item to be reported is unreasonable*<br><br>**89% consensus for deletion** | *Consensus on deletion obtained in Round 2: Item deleted.* | |

| Section and topic | Item # | PRISMA 2020 item | Candidate PRISMA-COSMIN item Round 1 | Revised candidate PRISMA-COSMIN item Round 2 | Revised candidate PRISMA-COSMIN item Round 3 | Revised candidate PRISMA-COSMIN item Workgroup meeting |
|---|---|---|---|---|---|---|
| | | | ~~each outcome measurement instrument: (a) the summarized or pooled result, and (b) the overall rating (i.e. against the COSMIN Criteria for Good Measurement Properties).~~<br><br>**89% consensus for inclusion**<br>**79% consensus for wording** | | | |
| | 20b | Present results of all ~~statistical~~ syntheses conducted. ~~If meta-analysis was done, present for each the summary estimate and its precision (e.g. confidence/credible interval) and measures of statistical heterogeneity. If comparing groups, describe the direction of the effect.~~ | Present results of all ~~statistical~~ syntheses conducted~~(e.g. pooled results)~~. ~~If meta-analysis was done, present for each the summary estimate and its precision (e.g. confidence/credible interval) and measures of statistical heterogeneity.~~<br><br>**93% consensus for inclusion**<br>**85% consensus for wording** | Present results of all synthesis conducted. For each measurement property, present~~, for each outcome measurement instrument~~: (a) the summarized or pooled result and (b) the overall rating against quality criteria.<br><br>**90% consensus for inclusion**<br>**77% consensus for wording** | Present results of all synthes~~i~~s conducted. For each measurement property of an outcome measurement instrument, present: (a) the summarized or pooled result and (b) the overall rating against quality criteria.<br><br>**99% consensus for inclusion**<br>**92% consensus for wording** | Present results of all syntheses conducted. For each measurement property of an outcome measurement instrument, present: (a) the summarized or pooled result and (b) the overall rating against quality criteria. |
| | 20c | Present results of all investigations of | Present results of all investigations of | Present results of all investigations of | *Consensus on inclusion obtained in Round 2.* | |

| Section and topic | Item # | PRISMA 2020 item | Candidate PRISMA-COSMIN item Round 1 | Revised candidate PRISMA-COSMIN item Round 2 | Revised candidate PRISMA-COSMIN item Round 3 | Revised candidate PRISMA-COSMIN item Workgroup meeting |
|---|---|---|---|---|---|---|
| | | possible causes of ~~heterogeneity~~ among study results. | possible causes of inconsistency among study results.<br><br>**89% consensus for inclusion**<br>**85% consensus for wording** | possible causes of inconsistency among study results.<br><br>**94% consensus for inclusion**<br>**86% consensus for wording** | | |
| | 20d | Present results of all sensitivity analyses conducted to assess the robustness of the synthesized results. | Present results of all ~~subgroup~~ analyses ~~if~~ conducted.<br><br>**93% consensus for inclusion**<br>**94% consensus for wording** | Present results of all sensitivity analyses conducted to assess the robustness of the synthesized results.<br><br>**79% consensus for inclusion**<br>**75% consensus for wording** | If applicable, present results of all sensitivity analyses conducted to assess the robustness of the synthesized results.<br><br>**91% consensus for inclusion**<br>**95% consensus for wording** | *Consensus on inclusion obtained in Round 3.* |
| Reporting biases | 21 | ~~Present assessments of risk of bias due to missing results (arising from reporting biases) for each synthesis assessed.~~ | *Not applicable – it is impossible to know whether reporting/publication bias exists and hence this cannot be assessed*<br><br>**80% consensus for deletion** | *Consensus on deletion obtained in Round 1: Item deleted.* | | |
| Certainty of evidence | 22 | Present assessments of certainty (or confidence) in the body of evidence for each ~~outcome~~ assessed. | Present assessments of certainty (or confidence) in the body of evidence for each measurement property for each | Present assessments of certainty (or confidence) in the body of evidence for each measurement property ~~for~~ each | Present assessments of certainty (or confidence) in the body of evidence for each measurement property of ~~each~~ | Present assessments of certainty (or confidence) in the body of evidence for each measurement property of an outcome measurement instrument assessed. |

| Section and topic | Item # | PRISMA 2020 item | Candidate PRISMA-COSMIN item Round 1 | Revised candidate PRISMA-COSMIN item Round 2 | Revised candidate PRISMA-COSMIN item Round 3 | Revised candidate PRISMA-COSMIN item Workgroup meeting |
|---|---|---|---|---|---|---|
| | | | outcome measurement instrument assessed.<br><br>**88% consensus for inclusion**<br>**83% consensus for wording** | outcome measurement instrument assessed.<br><br>**92% consensus for inclusion**<br>**91% consensus for wording** | outcome measurement instrument assessed.<br><br>**95% consensus for inclusion**<br>**95% consensus for wording** | |
| Interpretability and feasibility | PC6 | | Describe interpretability and feasibility aspects ~~per~~ outcome measurement instrument.<br><br>**80% consensus for inclusion**<br>**74% consensus for wording** | Describe interpretability and feasibility aspects for each outcome measurement instrument.<br><br>**84% consensus for inclusion**<br>**87% consensus for wording** | *Consensus on inclusion obtained in Round 2.* | |
| Recommendations | PC7 | | Describe the recommendations for use of the outcome measurement instrument(s) in the intended population and setting~~, e.g. COSMIN Recommendation Category A, B, or C~~.<br><br>**78% consensus for inclusion**<br>**85% consensus for wording** | ~~Describe the~~ recommendations for ~~use of the~~ outcome measurement instrument~~(s) in the intended population and setting~~.<br><br>**88% consensus for inclusion**<br>**87% consensus for wording** | If ~~applicable~~, make recommendations for suitable outcome measurement instruments for a particular use.<br>**91% consensus for inclusion**<br>**94% consensus for wording** | If appropriate, make recommendations for suitable outcome measurement instruments for a particular use. |
| **DISCUSSION** | | | | | | |
| Discussion | 23a | Provide a general interpretation of the | Provide a general interpretation of the | *Consensus on inclusion obtained in Round 1.* | | |

| Section and topic | Item # | PRISMA 2020 item | Candidate PRISMA-COSMIN item Round 1 | Revised candidate PRISMA-COSMIN item Round 2 | Revised candidate PRISMA-COSMIN item Round 3 | Revised candidate PRISMA-COSMIN item Workgroup meeting |
|---|---|---|---|---|---|---|
| | | results in the context of other evidence. | results in the context of other evidence.<br>**98% consensus for inclusion**<br>**97% consensus for wording** | | | |
| | 23b | Discuss any limitations of the evidence included in the review. | Discuss any limitations of the evidence included in the review.<br>**96% consensus for inclusion**<br>**97% consensus for wording** | *Consensus on inclusion obtained in Round 1.* | | |
| | 23c | Discuss any limitations of the review processes used. | Discuss any limitations of the review processes used.<br>**98% consensus for inclusion**<br>**96% consensus for wording** | *Consensus on inclusion obtained in Round 1.* | | |
| | 23d | Discuss implications of the results for practice, policy, and future research. | Discuss implications of the results for practice, policy, and future research.<br>**96% consensus for inclusion**<br>**96% consensus for wording** | *Consensus on inclusion obtained in Round 1.* | | |
| **OTHER INFORMATION** | | | | | | |
| Registration and protocol | 24a | Provide registration information for the | Provide registration information for the | *Consensus on inclusion obtained in Round 1.* | | |

| Section and topic | Item # | PRISMA 2020 item | Candidate PRISMA-COSMIN item Round 1 | Revised candidate PRISMA-COSMIN item Round 2 | Revised candidate PRISMA-COSMIN item Round 3 | Revised candidate PRISMA-COSMIN item Workgroup meeting |
|---|---|---|---|---|---|---|
| | | review, including register name and registration number, or state that the review was not registered. | review, including register name and registration number, or state that the review was not registered.<br>**95% consensus for inclusion**<br>**98% consensus for wording** | | | |
| | 24b | Indicate where the review protocol can be accessed, or state that a protocol was not prepared. | Indicate where the review protocol can be accessed, or state that a protocol was not prepared.<br>**90% consensus for inclusion**<br>**95% consensus for wording** | *Consensus on inclusion obtained in Round 1.* | | |
| | 24c | Describe and explain any amendments to information provided at registration or in the protocol. | Describe and explain any amendments to information provided at registration or in the protocol.<br>**85% consensus for inclusion**<br>**93% consensus for wording** | *Consensus on inclusion obtained in Round 1.* | | |
| Support | 25 | Describe sources of financial or non-financial support for the review, and the role of the | Describe sources of financial or non-financial support for the review, and the role of the | *Consensus on inclusion obtained in Round 1.* | | |

| Section and topic | Item # | PRISMA 2020 item | Candidate PRISMA-COSMIN item Round 1 | Revised candidate PRISMA-COSMIN item Round 2 | Revised candidate PRISMA-COSMIN item Round 3 | Revised candidate PRISMA-COSMIN item Workgroup meeting |
|---|---|---|---|---|---|---|
| | | funders or sponsors in the review. | funders or sponsors in the review.<br><br>**99% consensus for inclusion**<br>**97% consensus for wording** | | | |
| Author contributions | PC8 | | | Describe the contributions of ~~review~~ authors to the review.<br><br>**76% consensus for inclusion**<br>**88% consensus for wording** | ~~Describe the contributions of the authors to the review.~~<br><br>**71% consensus for inclusion**<br>**88% consensus for wording** | *Consensus on deletion obtained in workgroup meeting.* |
| Competing interests | 26 | Declare any competing interests of review authors. | Declare any competing interests of review authors.<br><br>**98% consensus for inclusion**<br>**96% consensus for wording** | *Consensus on inclusion obtained in Round 1.* | | |
| Availability of data, code, and other materials | 27 | Report which of the following are publicly available and where they can be found: template data collection forms; data extracted from included studies; data used for all analyses; analytic code; any other materials used in the review. | Report which of the following are publicly available and where they can be found: template data collection forms; data extracted from included studies; data used for all analyses; analytic code; any other materials used in the review. | *Consensus on inclusion obtained in Round 1.* | | |

| Section and topic | Item # | PRISMA 2020 item | Candidate PRISMA-COSMIN item Round 1 | Revised candidate PRISMA-COSMIN item Round 2 | Revised candidate PRISMA-COSMIN item Round 3 | Revised candidate PRISMA-COSMIN item Workgroup meeting |
|---|---|---|---|---|---|---|
| | | | **86% consensus for inclusion**<br>**96% consensus for wording** | | | |
