# Supplementary material for: Guideline for reporting systematic reviews of outcome measurement instruments (OMIs): PRISMA-COSMIN for OMIs 2024
Source: J Patient Rep Outcomes. 2024 Jul 9;8:64. doi: 10.1186/s41687-024-00727-7 (PMC11231111; doi:10.1186/s41687-024-00727-7)
Supplement: Supplementary file 5 [file 41687_2024_727_MOESM5_ESM.pdf]

PRISMA - COSMIN OUTCOME Measurement Instruments

Guideline for Reporting Systematic Reviews of Outcome Measurement Instruments

Explanation & Elaboration

Ellen BM Elsman
Lidwine B Mokkink
Caroline B Terwee
Dorcas Beaton
Nancy J Butcher
Joel J Gagnier
Andrea C Tricco
Olalekan Lee Aiyegbusi
Anna Berardi
Julie Farmer
Kirstie L Haywood
Karolin R Krause
Evan Mayo-Wilson
Ava Mehdipour
Peter Szatmari
Zahi Touma
Catherine Hofstetter
Sarah Markham
Juanna Ricketts
Maureen Smith
David Moher
Martin Offringa

January 2024

Content

PRISMA-COSMIN for OMIs 2024 2
Background 2
This guideline 2
Explanation & Elaboration 3
Title 7
Title 7
Abstract – Open Science 8
Funding 8
Registration 9
Abstract – Background 10
Objectives 10
Abstract – Methods 12
Eligibility criteria 12
Information sources 13
Risk of bias 14
Measurement properties 15
Synthesis methods 16
Abstract – Results 17
Included studies 17
Synthesis of results 18
Abstract – Discussion 19
Limitations of evidence 19
Interpretation 20
Abstract – Examples containing all Abstract reporting items 21
Plain language summary 23
Plain language summary 23
Open Science 25
Registration and protocol 25
Support 28
Competing interests 29
Availability of data, code, and other materials 30
Introduction 31
Rationale 31
Objectives 33
Methods 35

Followed guidelines 35
Eligibility criteria 36
Information sources 38
Search strategy 40
Selection process 45
Data collection process 47
Data items 48
Study risk of bias assessment 49
Measurement properties 51
Synthesis methods 55
Certainty assessment 60
Formulating recommendations 62
Results 63
Study selection 63
OMI characteristics 69
Study characteristics 73
Risk of bias in studies 75
Results of individual studies 80
Results of syntheses 82
Recommendations 90
Discussion 91
Discussion 91
References 96

PRISMA-COSMIN for OMIs 2024

PRISMA: **P**referred **R**eporting **I**tems for **S**ystematic reviews and **M**eta-**A**nalyses
COSMIN: **CO**nsensus-based **S**tandards for the selection of health **M**easurement **IN**struments
OMI: **O**utcome **M**easurement **I**nstrument

Background

OMIs are used by healthcare professionals or researchers to measure outcomes and refer to how an outcome is measured (e.g., with a questionnaire, performance-based test, lab test, or single rating scale). Systematic reviews evaluating the quality of OMIs are important in the evidence-based selection of the most appropriate OMI. COSMIN has developed a comprehensive and widespread guideline to conduct these systematic reviews; however, key information is often missing in published reports. This hinders the appraisal of the quality of OMIs, and impacts the decisions of users (e.g., researchers, healthcare providers, patients and policymakers) regarding the appropriateness of an OMI. Until now, authors of OMI systematic reviews have been encouraged to complete and adhere to the widely used PRISMA 2020 guideline.[1] This guideline does not include all essential information for systematic reviews of OMIs, limiting the reproducibility (ability to replicate the results using the same data) and interpretability (the ability to understand and interpret the findings) of such reviews. PRISMA-COSMIN for OMIs 2024 aims to harmonize reporting of systematic reviews of OMIs.

This guideline

PRISMA-COSMIN for OMIs 2024 is a stand-alone extension of PRISMA 2020, specifically intended for reporting systematic reviews of OMIs where *at least one measurement property of at least one OMI is evaluated*. PRISMA-COSMIN for OMIs 2024 is not intended for reviews that only provide an overview (i.e., characteristics) of the OMIs used, as these review types are more scoping in nature. Moreover, PRISMA-COSMIN for OMIs 2024 is **not a quality assessment instrument or risk of bias tool to gauge the quality of a systematic review** and should not be confused with tools that have specifically been developed for that purpose.

PRISMA-COSMIN for OMIs 2024 is intended for all systematic reviews of OMIs, conducted with any methodology or tools; it does not specifically apply to systematic reviews conducted with the methodology or tools from the COSMIN initiative.

PRISMA-COSMIN for OMIs 2024 might also be used to report systematic reviews of instruments other than *outcome* measurement instruments, for example systematic reviews in which experience measures or process measures are being evaluated. It depends however on the methodology that is used to conduct those systematic reviews if PRISMA-COSMIN for OMIs 2024 is applicable to these systematic reviews.

PRISMA-COSMIN for OMIs 2024 consists of:

- A checklist for **full reports** containing 54 (sub)items, including the abstract items
- A checklist for **abstracts** (journal or conference abstracts) containing 13 items
- An Explanation & Elaboration (E&E) document for **full reports** and abstracts (this document)
- An Explanation & Elaboration (E&E) document for **abstracts**
- A flow diagram (available for download and included in this document)

Explanation & Elaboration

In this document, we explain why reporting of each item is recommended, with evidence supporting the inclusion of the item whenever possible. We present bullet points that detail the reporting recommendations and include exemplars of good reporting from published open access systematic reviews of OMIs. This structure is similar to the structure of the Explanation & Elaboration (E&E) document of PRISMA 2020.[2] Where possible, we used the exact same wording and phrasing as the PRISMA 2020 E&E, published open access and distributed under the terms of the Creative Commons CC BY 4.0 license, to facilitate implementation of the guidance. Note, for this extension of PRISMA 2020 a few generic items were added as well, including Plain Language Summary and Open Science items.

We encourage authors to use this document in conjunction with the checklist(s). Box 1 includes a glossary of terms used throughout PRISMA-COSMIN for OMIs 2024.

Box 1. Glossary of terms used in PRISMA-COSMIN for OMIs 2024

Systematic review
A study design that uses explicit, systematic methods to collect data from primary studies, critically appraises the data, and synthesizes the findings descriptively or quantitatively in order to address a clearly formulated research question.[2-4] Typically, a systematic review includes a clearly stated objective, pre-defined eligibility criteria for primary studies, a systematic search that attempts to identify all studies that meet the eligibility criteria, risk of bias assessments of the included primary studies, and a systematic presentation and synthesis of findings of the included studies.[4] Systematic reviews can provide high quality evidence to guide decision making in healthcare, owing to the trustworthiness of the findings derived through systematic approaches that minimize bias.[5]

Outcome domain
Refers to *what* is being measured (e.g., fatigue, physical function, blood glucose, pain).[6,7] Other terms include construct, concept, latent trait, factor, attribute.

Outcome measurement instrument (OMI)
Refers to *how* the outcome is being measured, i.e., the OMI used to measure the outcome domain. Different types of OMIs exist such as questionnaires or patient-reported outcome measures (PROMs) and their variations, clinical rating scales, performance-based tests, laboratory tests, scores obtained through a physical examination or observations of an image, or responses to single questions.[6,7] An OMI consists of a set of components and phases, i.e., 'equipment', 'preparatory actions', 'collection of raw data', 'data processing and storage', and 'assignment of the score'.[8] A specific type of OMIs is clinical outcome assessments (COAs),[9] which specifically focus on outcomes related to clinical conditions, often emphasizing the patient's experience and perspective.

Report
A document with information about a particular study or a particular OMI. It could be a journal article, preprint, conference abstract, study register entry, clinical study report, dissertation, unpublished manuscript, government report, or any other document providing relevant information such as a manual for an OMI or the PROM itself.[2] A **study report** is a document with information about a particular study like a journal article or a preprint.

Record
The title and/or abstract of a report indexed in a database or website. Records that refer to the same report (such as the same journal article) are "duplicates".[2]

Study
The empirical investigation of a measurement property in a specific population, with a specific aim, design and analysis.

Quality
The technical concept 'quality' is used to address three different aspects defined by COSMIN, OMERACT, and GRADE: 1) quality of the OMI refers to the measurement properties; 2) quality of the study refers to the risk of bias; and 3) quality of the evidence refers to the certainty assessment.[7,10,11]

Measurement properties
The quality aspects of an OMI, referring to the validity, reliability, and responsiveness of the instrument's score.[12] Each measurement property requires its own study design and statistical methods for evaluation. Different definitions for measurement properties are being used. COSMIN has a taxonomy with consensus-based definitions for measurement properties.[12] Another term for measurement properties is psychometric properties.

Feasibility
The ease of application and the availability of an OMI, e.g., completion time, costs, licensing, length of an OMI, ease of administration, etc.[10,13] Feasibility is not a measurement property, but is important when selecting an OMI.[7]

Interpretability
The degree to which one can assign meaning to scores or change in scores of an OMI in particular contexts (e.g., if a patient has a score of 80, what does this mean?).[12] Norm scores, minimal important change and minimal important difference are also relevant concepts related to interpretability. Like feasibility, interpretability is not a measurement property, but is important to interpret the scores of an OMI and when selecting an OMI.[7]

Measurement properties' results
The findings of a study on a measurement property. Measurement properties' results have different formats, depending on the measurement property. For example, reliability results might be the estimate of the intraclass correlation coefficient (ICC), or structural validity results might be the factor loadings of items to their respective scales and the percentage of variance explained.

Measurement properties' ratings
The comparison of measurement properties' results against quality criteria, to give a judgement (i.e., rating) about the results. For example, the ICC of an OMI might be 0.75; this is the result. A quality criterion might prescribe that the ICC should be >0.7. In this case the result (0.75) is thus rated to be sufficient.

Risk of bias
Risk of bias refers to the potential that measurement properties' results in primary studies systematically deviate from the truth due to methodological flaws in the design, conduct or

analysis.[2,14] Many tools have been developed to assess the risk of bias in primary studies. The COSMIN Risk of Bias checklist for PROMs was specifically developed to evaluate the risk of bias of primary studies on measurement properties.[15] It contains standards referring to design requirements and preferred statistical methods of primary studies on measurement properties, and is specifically intended for PROMs. The COSMIN Risk of Bias tool to assess the quality of studies on reliability or measurement error of OMIs can be used for any type of OMI.[8]

Synthesis
Combining quantitative or qualitative results of two or more studies on the same measurement property and the same OMI. Results can be synthesized quantitatively or qualitatively. Meta-analysis is a statistical method to synthesize results. Although this can be done for some measurement properties (i.e., internal consistency, reliability, measurement error, construct validity, criterion validity, responsiveness), it is not very common in systematic reviews of OMIs because the point estimates of the results are not used. Instead, the score obtained with an OMI is used. End-users therefore only need to know whether the result of a measurement property is sufficient or not. For some measurement properties (i.e., content validity, structural validity, cross-cultural validity/measurement invariance) it is not even possible to statistically synthesize the results by meta-analysis or pooling. In general, most often the robustness of the results is described (e.g., the found factor structure, the number of confirmed and unconfirmed hypotheses), or a range of the results is provided (e.g., the range of Cronbach's alphas or ICCs).

Certainty (or confidence) assessment
Together with the **synthesis**, often an assessment of the certainty (or confidence) in the body of evidence is provided. Authors conduct such an assessment to reflect how certain (or confident) they are that the synthesized result is trustworthy. These assessments are often based on established criteria, which include the risk of bias, consistency of findings across studies, sample size, and directness of the result to the research question.[7] A common framework for the assessment of certainty (or confidence) is GRADE (Grading of Recommendations Assessment, Development, and Evaluation).[11] A modified GRADE approach has been developed for communicating the certainty (or confidence) in systematic reviews of OMIs.[7]

OMI recommendations
Systematic reviews of OMIs provide a comprehensive overview of the measurement properties of OMIs and support evidence-based recommendations for the selection of suitable OMIs for a particular use. Unlike systematic reviews of interventions, systematic reviews of OMIs often make recommendations about the suitability of OMIs for a particular use, although in some cases this might not be appropriate (e.g., if restricted by the funder). Making recommendations also facilitates much needed standardization in use of OMIs, although their quality and score interpretation might be context dependent. Making recommendations essentially involves conducting a synthesis at the level of the OMI, across different measurement properties, taking feasibility and interpretability into account as well. Various methods and tools for OMI recommendation exist (e.g., from COSMIN, OMERACT and others).[7,16,17]

Most of the following information has been reused from Page et al., 2021.[2] We used standardized language in the E&E to indicate whether reporting recommendations for each item (referred to as "elements" throughout) are essential or additional. *Essential elements* should be reported in the

main report or included in the supplementary material for all systematic reviews of OMIs (except for those preceded by “If…,” which should only be reported where applicable). These have been selected as essential because transparent and complete reporting of these elements is important for users to assess the trustworthiness and applicability of a review’s findings, or their reporting would aid in reproducing the findings. *Additional elements* are those which are not essential but provide supplementary information that may enhance the completeness and usability of systematic review reports. Finally, although PRISMA-COSMIN for OMIs 2024 provides a template for where information might be located, the suggested location should not be seen as prescriptive; the guiding principle is to ensure the information is reported. This can either be in the text of the main report, in tables or figures, or as supplementary material.

Journals and publishers might impose word and section limits, and limits on the number of tables and figures allowed in the main report. In such cases, if the relevant information for some items already exists (e.g., in open document repositories or other reports), providing a reference or link to the information may suffice.

We found reporting exemplars for each checklist item from published systematic reviews of OMIs. We have edited the examples by removing all citations within them (to avoid potential confusion with the citation for each example) and removing names and/or initials of authors. We have spelled out abbreviations to aid comprehension, except when this concerned names of OMIs, which we printed in *italic*. On page 21-22 of this document, we also provide two fictional examples of 350-word abstracts in which all Abstract reporting items are included.

Title

Title

Item #1: Identify the report as a systematic review and include as applicable the following (in any order): outcome domain of interest, population of interest, name/type of OMIs of interest, and measurement properties of interest.

Explanation: Inclusion of "systematic review" in the title facilitates identification by potential users (patients, healthcare providers, policy makers, researchers, etc.) and appropriate indexing in databases.[2] Terms such as "review", "literature review", "evidence synthesis", or "knowledge synthesis" are not recommended because they do not distinguish systematic and non-systematic approaches.[2] The objective or question that the systematic review addresses often includes four key elements: the outcome domain, population, name or type of OMI and the measurement properties.[7] It is therefore recommended to include these four key elements in the title of the review, if word count permits, unless certain key elements are clearly irrelevant or redundant. For example, if the objective of the review is to evaluate the measurement properties of a certain OMI in a specific population, it might be irrelevant to include the outcome if that is clear from the name of the OMI. If multiple measurement properties are evaluated in the review, authors can state "measurement properties" or "quality" instead of listing each of the measurement properties. If multiple OMIs are evaluated in the review, authors can state the type of OMI (for example patient-reported outcome measures (PROMs) or performance-based tests). If different types of OMIs are evaluated in the review, authors can state "outcome measurement instruments".

Essential elements

- Identify the report as a systematic review in the title.[2]
- Report an informative title that provides key information about the main objective or question that the review addresses, for example with respect to the outcome domain of interest, population of interest, name/type of OMI of interest, and/or measurement properties of interest (which can also be referred to as the quality of the OMIs).[7]

Example of item #1

Example 1: "Systematic review on the measurement properties of diabetes-specific patient-reported outcome measures (PROMs) for measuring physical functioning in people with type 2 diabetes"[18]

Example 2: "Content Validity of Patient-Reported Outcome Measures Developed for Assessing Health-Related Quality of Life in People with Type 2 Diabetes Mellitus: a Systematic Review"[19]

Example 3: "A systematic review of the measurement properties of the Body Image Scale (BIS) in cancer patients"[20]

Abstract – Open Science

Funding

Item #2.2:[a] Specify the primary source of funding for the review.

Explanation: As with any research report, authors should be transparent about the sources of funding received to conduct the systematic review.[2] The abstract should include the main source of funding for the systematic review, whether from host institutions or from external funders,[21] unless the journal has a designated section to report this information. For conference abstracts, this information should always be reported.

Essential elements

- Specify the main funding source for the systematic review.

Example of item #2.2

*"*Funding: The source of funding: Frans Huygen Stichting."[22]

[a] Item #2.1 in the PRISMA-COSMIN for OMIs 2024 Abstracts checklist refers to the title. Item #2.1 in the Abstracts checklist is identical to item #1 in the Full Report checklist.

Registration

Item #2.3: Provide the register name and registration number.

Explanation: Registration of systematic reviews provides a record of reviews that have been initiated, even if they have not been published.[21] It is therefore a means of alerting researchers to systematic reviews that are in progress, and serves as a public record of the proposed systematic review.[21] Registration also helps to detect reporting bias (i.e., publication bias) by enabling better identification of unpublished systematic reviews.[21,23] The abstract should record the name of the database with which the review is registered, and the registration number,[21] unless the journal has a designated section to report this information. For conference abstracts, this information should always be reported.

Essential elements

- Provide registration information for the review, including register name and registration number, or state that the review was not registered.[2]

Example of item #2.3

Example 1: "This systematic review was registered in the PROSPERO international prospective register of systematic reviews, with registration number CRD42019130936."[24]

Example 2: "PROSPERO registration CRD42021282032"[25]

Abstract – Background

Objectives

Item #2.4: Provide an explicit statement of the main objective(s) or question(s) the review addresses.

Explanation: The objectives in an abstract should convey succinctly the aim or research question the systematic review addresses.[21] An explicit and concise statement of the main review objective(s) or question(s) will help readers understand the scope of the review.[2] Such statements may be written in the form of aims or objectives ("… to examine the measurement properties of…") or as questions ("what are the measurement properties of….?", "what is the quality of…").[2,26] The objective or question that the systematic review addresses often includes four key elements: the outcome domain, population, name or type of OMI, and the measurement properties.[7] It is therefore recommended to include these four key elements in the objective(s) or question(s) the review addresses, unless certain key elements are clearly irrelevant or redundant. For example, if the objective of the review is to evaluate the measurement properties of a certain OMI in a specific population, it might be irrelevant to include the outcome if that is clear from the name of the OMI. If multiple measurement properties are evaluated in the review, authors can state "measurement properties" or "quality" instead of listing each of the measurement properties. If multiple OMIs are evaluated in the review, authors can state the type of OMI (for example patient-reported outcome measures (PROMs) or performance-based tests). If different types of OMIs are evaluated in the review, authors can state "outcome measurement instruments".

The objective or question could also be linked to the rationale for the systematic review, for example, to provide an overview of the quality of available OMIs or to select the best OMI for a particular use (e.g., in a core outcome set or a clinical trial).

Essential elements

- Provide an explicit statement of the main objective(s) or question(s) the review addresses.[2]
- Use the four key elements (outcome domain, population, name or type of OMI and the measurement properties of interest) as applicable to formulate the objective(s) or question(s).[7]

Additional elements

- Consider linking the main objective(s) or question(s) to the rationale for the review (for example, to provide an overview of the quality of available OMIs or to select the best OMI for a particular use).

Example of item #2.4

*Example 1: "*We aimed to systematically assess the measurement properties of diabetes-specific patient-reported outcome measures (PROMs) for measuring physical functioning, one of the core outcomes, in adults with type 2 diabetes."[18]

Example 2: "We aimed to systematically evaluate the content validity of patient-reported outcome measures (PROMs) specifically developed to measure (aspects of) health-related quality of life (HRQOL) in people with type 2 diabetes."[19]

Example 3: “The aim of this study was to systematically review measurement properties of the *BIS* among cancer patients.”[20]

Abstract – Methods

Eligibility criteria

Item #2.5: Specify the inclusion and exclusion criteria for the review.

Explanation: Specifying the main criteria used to decide what evidence was eligible should enable readers to understand the scope of the review and verify the inclusion decisions.[2] The inclusion and exclusion criteria often relate to the four key elements: the outcome domain, population, name or type of OMI, and the measurement properties.[7] For a study to be included, often the aim should be to evaluate one or more of the measurement properties of interest, report on the development of an OMI, or report on its interpretability and feasibility aspects.[7]

Essential elements

- Briefly specify the main study characteristics used to decide whether a study was eligible for inclusion in the review, which can include the outcome domain, population, name/type of OMI, and/or measurement properties of interest,[7] and other characteristics, such as eligible study design(s) and setting(s).

Additional elements

- Consider specifying eligibility criteria with regard to report characteristics, such as year of dissemination, language, and report status (for example, whether reports such as unpublished manuscripts and conference abstracts were eligible for inclusion).[2]

Example of item #2.5

Example 1: "Eligible studies were peer-reviewed English language publications that sampled a population of children with mean age between 5 and 12 years and focused on developing and evaluating at least one psychometric property of a teacher proxy-report instrument for assessing one or more of the 30 APLF [Australian Physical Literacy Framework] elements."[24]

Example 2: "Studies reporting on the development and/or validation of any PROMs [patient-reported outcome measures] for uncomplicated UTIs [urinary tract infections] in women were considered eligible."[27]

Example 3: "Studies on development of the *LEFS* and/or the evaluation of one or more measurement properties of the *LEFS* in patients with lower extremity fractures were included […]."[28]

Information sources

Item #2.6: Specify the information sources (e.g., databases, registers) used to identify studies and the date when each was last searched.

Explanation: Authors should provide a brief description of the information sources searched or consulted, including the dates when each source was last searched, to allow readers to assess the completeness and currency of the systematic review.[2] If multiple information sources were used, the total number of databases could be specified instead. In the abstract, it is sufficient to state the month and year information sources were searched.

Essential elements

- Specify the date (month and year) when each source (such as database, register, website, organization) was last searched or consulted.[2]
- If bibliographic databases were searched, specify for each database its name (such as MEDLINE, CINAHL) or state the number of databases searched if multiple databases were searched.

Additional elements

- If study registers (such as PROSPERO), and other online repositories (such as the COSMIN database) were searched, consider specifying the name of each source and any restrictions that were applied.[2]

Example of item #2.6

*Example 1: "*MEDLINE, Embase, AMED and PsycINFO were searched from inception to 1 July 2020 […] unlimited by publication date or language."[29]

Example 2: "[…] we reviewed empirical research published from 1980 through February 2020 with an updated search in March 2021 in Medline, Embase, PsycINFO, Health and Psychological Instruments, CINAHL, ERIC, and Web of Science databases."[30]

Example 3: "Nine databases were searched from January 1996 to October 2020."[31]

Risk of bias

Item #2.7: Specify the methods used to assess risk of bias in the included studies.

Explanation: Limitations in the design and conduct of individual studies can raise questions about the internal validity of their findings.[14] An important aspect of a systematic review is therefore to assess the validity of individual studies by means of a risk of bias assessment.[21] Risk of bias refers to the potential for study findings to systematically deviate from the truth due to methodological flaws in the design, conduct or analysis.[14] Authors should describe the methods used to assess risk of bias in the included studies.[21] If the review was conducted following established guidance (e.g., the COSMIN guideline for systematic reviews or the OMERACT filter), items #2.7, #2.8 and #2.9 can be summarized into one general statement, as it can be inferred that the tools and methods within the guidance were used (see examples 4 and 5).

Essential elements

- Specify the method(s) used to assess risk of bias in the included studies.
- If the review was conducted following established guidance, methods used to assess risk of bias (item #2.7), rate the results of a measurement property (item #2.8), and synthesize the results (item #2.9) can be summarized into a general statement referring to that guidance.

Example of item #2.7

Example 1: "Methodological quality of the included studies was evaluated using the Consensus-based Standards for the selection of health Measurement Instruments (COSMIN) risk of bias checklist."[32]

Example 2: "For critical appraisal, the COSMIN Risk of Bias tool for reliability and measurement of error was used."[33]

Example 3: "The Agency for Healthcare Research and Quality (AHRQ) checklists was used to assess the risk of bias for each included study."[34]

Example 4: "Following the OMERACT Filter 2.1 instrument selection process, [...]."[35]

Example 5: "Data extraction and quality assessment (including a risk of bias evaluation) of the included studies was undertaken [...] in accordance with COSMIN guidelines."[36]

Measurement properties

Item #2.8: Specify the methods used to rate the results of a measurement property.

Explanation: To interpret the results, readers need to know how the results of a measurement property were rated. Authors should describe the methods used to rate the results of a measurement property, both for each individual study and for the summarized or pooled results (if different). If the review was conducted following established guidance (e.g., the COSMIN guideline for systematic reviews or the OMERACT filter), items #2.7, #2.8, and #2.9 can be summarized into one general statement, as it can be inferred that the tools and methods within the guidance were used (see examples 4 and 5).

Essential elements

- Specify the method(s) used to rate the results of a measurement property.
- If the review was conducted following established guidance, methods used to assess risk of bias (item #2.7), rate the results of a measurement property (item #2.8), and synthesize the results (item #2.9) can be summarized into a general statement referring to that guidance.

Example of item #2.8

Example 1: “The COSMIN criteria for good measurement properties were used to judge the results of the studies [...].”[37]

Example 2: “The measurement properties were then scored using quality criteria (positive/negative/indeterminate).”[38]

Example 3: “Furthermore, available evidence of the reliability, validity, responsiveness, and interpretability of the included scales was rated according to published quality criteria.”[39]

Example 4: “Following the OMERACT Filter 2.1 instrument selection process, [...].”[35]

Example 5: "Data extraction and quality assessment (including a risk of bias evaluation) of the included studies was undertaken [...] in accordance with COSMIN guidelines.”[36]

Synthesis methods

Item #2.9: Specify the methods used to present and synthesize results.

Explanation: Results of multiple studies on a measurement property are mostly synthesized by summarizing them qualitatively. For some measurement properties (i.e., internal consistency, reliability, measurement error, construct validity, responsiveness), results can be pooled or meta-analyzed, although this is not commonly done in systematic reviews of OMIs, because the point estimates of these results are generally not used. The methods used to synthesize the results should be specified in the abstract. If word count permits, details on the assessment of certainty (or confidence) can also be provided. If the review was conducted following established guidance (e.g., according to the COSMIN guideline for systematic reviews or the OMERACT filter), items #2.7, #2.8, and #2.9 can be summarized into a general statement, as it can be inferred that the tools and methods within the guidance were used (see examples 4 and 5).

Essential elements

- Report the methods used to synthesize the results.
- If meta-analysis was done, specify the meta-analysis model.
- If the review was conducted following established guidance, methods used to assess risk of bias (item #2.7), rate the results of a measurement property (item #2.8), and synthesize the results (item #2.9) can be summarized into a general statement referring to that guidance.

Additional elements

- Consider providing details about the certainty (or confidence) assessment.

Example of item #2.9

Example 1: "Data analysis and synthesis followed COSMIN methodology for reviews of outcome measurement instruments."[40]

Example 2: "Extracted evidence was qualitatively synthesized and evaluated […]."[41]

Example 3: "We used the COSMIN criteria to summarize and rate the psychometric properties of each PROM [patient-reported outcome measure]. A modified Grading, Recommendations, Assessment, Development, and Evaluation (GRADE) system was used to assess the certainty of evidence."[31]

Example 4: "Following the OMERACT Filter 2.1 instrument selection process, [...]."[35]

Example 5: "Data extraction and quality assessment (including a risk of bias evaluation) of the included studies was undertaken [...] in accordance with COSMIN guidelines."[36]

Abstract – Results

Included studies

Item #2.10: Give the total number of included OMIs and study reports.

Explanation: Providing the number of included OMIs and reports enables readers to understand the extent of the evidence included in the systematic review. If different versions of the same OMI are found, this should also be reported because each version of an OMI is considered a separate OMI (except for language versions).[7]

Essential elements

- Report the total number of OMIs included in the review.
- Report the total number of reports included in the review.

Additional elements

- Consider reporting the number of separate versions of OMIs included in the review.

Example of item #2.10

Example 1: "Out of 6423 screened publications, 32 original articles were eligible for inclusion in this review, reporting evidence on the measurement properties of 22 self- and/or proxy-reported questionnaires (including seven cultural adaptations) for various pediatric orthopedic conditions, including cerebral palsy (CP) and obstetric brachial plexus palsy (OBPP)."[36]

Example 2: "In total 21 articles were included, describing 12 versions of 7 unique diabetes-specific PROMs or subscales measuring physical functioning."[18]

Example 3: "We included 24 articles describing the development and/or evaluation of 21 instruments."[22]

Synthesis of results

Item #2.11: Present the syntheses of results of OMIs, indicating the certainty of the evidence.

Explanation: The main syntheses of results (i.e., those most relevant to the aim of the review) should be given in the abstract.[21] For example, if a study evaluates all measurement properties but pre-specified that content validity and structural validity were imperative for the conclusions, then syntheses of at least those measurement properties should be provided for the most relevant OMIs. Along with the syntheses of results, the certainty of the evidence for each of these syntheses could be provided, if word count permits, as this shows the confidence in the trustworthiness of the synthesized results.[7,42]

Essential elements

- Report the results of the main syntheses conducted.

Additional elements

- Consider reporting the overall level of certainty in the body of evidence (such as high, moderate, low, or very low) for each main synthesis.

Example of item #2.11

Example 1: In a review examining the measurement properties of diabetes-specific PROMs measuring physical functioning,[18] the authors pre-specified that at least sufficient content validity, structural validity, and internal consistency was needed for an OMI to be recommended. In the abstract, the authors report the results of these syntheses for the PROMs that were found to have sufficient ratings for these measurement properties, along with the certainty of the evidence for content validity.

"Both had sufficient ratings for aspects of content validity, although with mostly very low-quality evidence. Sufficient ratings for structural validity, internal consistency, and reliability were also found for both instruments, but responsiveness was rated inconsistent for both instruments. The other PROMs or subscales often had insufficient aspects of content validity, or their unidimensionality could not be confirmed."[18]

Example 2: In a review examining the validity and reliability of quality of life questionnaires in patients with ankylosing spondylitis and non-radiographic axial spondylarthritis,[34] the authors opted to present the syntheses of the instruments with the most favorable measurement properties.

"Cronbach's alpha (α) Coefficients were generally high (0.79–0.97) for overall scales. The ankylosing spondylitis quality of life (ASQOL) and evaluation of ankylosing spondylitis quality of life (EASi-QoL) questionnaires showed the strongest measurement properties in high-quality studies. The correlation coefficient for test–retest reliability of the ASQOL questionnaire was 0.85 (95% CI 0.80 to 0.89). The pooled Cronbach's α coefficients of the ASQOL questionnaire and the EASi-QoL questionnaire were high."[34]

Abstract – Discussion

Limitations of evidence

Item #2.12: Provide a brief summary of the limitations of the evidence included in the review (e.g., study risk of bias, inconsistency, and imprecision).

Explanation: The abstract should briefly describe the limitations of the evidence across studies.[21] Briefly summarizing the completeness, applicability, and uncertainties in the evidence included in the review should help readers interpret the findings appropriately.[2] For example, authors might acknowledge that they identified few eligible studies or studies with a small number of participants, leading to imprecision; have concerns about risk of bias in studies or missing results; found studies with conflicting results, leading to inconsistency; or identified studies that only partially or indirectly address the population of interest, leading to concerns about their relevance and applicability to particular patients, settings, or other target audiences.[2]

Essential elements

- Provide a brief summary of the limitations of the evidence included in the review (e.g., study risk of bias, inconsistency and imprecision).

Example of item #2.12

Example 1: "However, due to the high heterogeneity of the studies available, these results should not be considered conclusive."[43]

Example 2: "In interpreting the outcomes, one should therefore be aware that not all relevant aspects of physical functioning may be accounted for in the *LEFS*."[28]

Example 3: "The *HAQ*, however, was frequently associated with considerable ceiling effects while the *SF-36* has limited content coverage."[39]

Example 4: "The quantity and quality of the evidence on the other measurement properties of the included questionnaires varied substantially with insufficient sample sizes and/or poor methodological quality resulting in significant downgrading of evidence quality."[36]

Interpretation

Item #2.13: Provide a general interpretation of the results and important implications.

Explanation: To help readers interpret the results, an overall summary of the main findings should be given.[21] This could include an indication of what is clear, what important uncertainties remain, and whether further research is needed to address these.[21] If there is not enough evidence from well-conducted studies to answer the review's question, this should be made clear to the reader.[21] If the conclusions of the review differ substantially from previous systematic reviews, then some explanation might also be provided.[21] Possible implications for policy and practice should be stated.[21] The general interpretation and implications could be linked to the rationale of the review (for example, to provide an overview of the quality of available OMIs or to select the best OMI for a particular use (e.g., in a core outcome set or a clinical trial)).

Essential elements

- Provide a general interpretation of the results and important implications.

Additional elements

- Consider linking the general interpretation and important implications to the rationale for the review (for example to provide an overview of the quality of available OMIs or to select the best OMI for a particular use).

Example of item #2.13

Example 1: "We suggest considering the KDQOL-36 for use in pre-dialysis patients; the KDQOL-SF or KDQOL-36 for dialysis patients and the ESRD-SCLTM for use in transplant recipients. However, further research is required to evaluate the measurement error, structural validity, responsiveness and patient acceptability of PROMs [patient-reported outcome measures] used in CKD [chronic kidney disease]."[44]

Example 2: "The first studies into the Dutch–Flemish PROMIS-PF item bank and the UE [upper extremity] subdomain show promising results, with especially high quality evidence for sufficient structural validity and measurement precision. However, more studies, and with higher methodological quality, are needed to study the instruments derived from these item banks. These studies should also evaluate content validity, reliability and responsiveness."[37]

Example 3: "Our review shows there is extensive evidence on the internal consistency and structural validity of QoL [quality of life] instruments used on parents during pregnancy and the postpartum period, but that the evidence on other psychometric properties is sparse. Validation studies and primary studies are needed to provide evidence on the reliability, validity, responsiveness, and interpretability of QoL instruments for this target group, in particular for fathers and partners."[45]

Example 4: "Smartphone applications showed sufficient intra-rater reliability, inter-rater reliability, and validity to measure neck ROM [range of motion] in people with and without neck pain. However, the quality of evidence and the confidence in the findings are low. High-quality research with large sample sizes is needed to further provide evidence to support the measurement properties of smartphone applications for the assessment of neck ROM."[46]

Abstract – Examples containing all Abstract reporting items

Here, we provide two fictional examples that contain all Abstract reporting items within 350 words. These examples can be used by authors who are drafting their abstract, either for conferences or for journals. Example 1 is based on a conference abstract submitted to the 9th Annual PROMIS® International Conference by Stallwood et al.,[47] whereas example 2 is based on a journal abstract as published by Elsman et al., 2022.[18]

Example 1: **Measurement properties of pediatric PROMIS questionnaires for overall pediatric health: a systematic review**

Background: The International Consortium for Health Outcomes Measurement (ICHOM) recently developed a standard set for overall pediatric health outcomes in routine care, which recommends Patient-Reported Outcomes Measurement Information System (PROMIS) measures to measure global health and cognitive functioning.

Objective: To systematically evaluate whether the PROMIS Pediatric Scale v1.0- Global Health 7+2, PROMIS Parent Proxy Scale v1.0- Global Health 7+2, and the PROMIS Parent Proxy Short Form v1.0 - Cognitive Function 7a have sufficient measurement properties to be recommended for their target age groups in pediatric healthcare, according to the COnsensus-based Standards for the selection of health Measurement INstruments (COSMIN) guidelines.

Methods: Embase, PsycINFO, and Web of Science were searched from year of inception of the Outcome Measurement Instruments measures to May 25, 2020; MEDLINE was searched up to October 24, 2022. Studies were included if they reported on the development or aimed to evaluate at least one measurement property of the aforementioned PROMIS measures. We used the COSMIN guideline for systematic reviews to appraise eligible studies (e.g., risk of bias of studies and measurement properties' results), synthesize, and descriptively summarize the overall evidence to determine whether these measures can be recommended for use.

Results: Screening of over 4000 titles and abstracts yielded 4 to 6 eligible study reports for each measure. While all measures met the minimum COSMIN criteria for recommending its use (i.e., sufficient evidence for content validity, and at least low-quality evidence for sufficient structural validity and internal consistency), the quality of the evidence for content validity was low due to poor reporting.

Conclusion: The PROMIS measures evaluated in this review measure their intended construct for their targeted age group and are fit-for-purpose for child health outcome measurement. Implementation of standard outcome sets with measures that are valid, reliable, and responsive to change will lay the foundation for value-based child and adolescent healthcare. As most studies included in this review were conducted in English speaking populations, future research is needed to confirm if these measures are valid and reliable in other languages.

Funding: No funding was received for this study.

Registration: OSF: https://osf.io/vx92r/

Example 2: **Systematic review on the measurement properties of diabetes-specific patient-reported outcome measures (PROMs) for measuring physical functioning in people with type 2 diabetes**

Objective: To systematically assess the measurement properties of diabetes-specific patient-reported outcome measures (PROMs) for measuring physical functioning, one of the core outcomes, in adults with type 2 diabetes.

Methods: Studies reported in English were included if they reported on the development or validation of a diabetes-specific PROM or subscale measuring physical functioning. Embase and MEDLINE were searched from year of inception to January 1, 2022. Risk of bias was evaluated with the COSMIN Risk of Bias checklist. Measurement properties of PROMs or subscales were rated using Terwee's criteria. If multiple studies on the same measurement property for the same PROM were found, results were synthesized descriptively.

Results: In total 21 study reports were included, describing 12 versions of 7 unique diabetes-specific PROMs or subscales measuring physical functioning. In general, there were few high-quality studies on measurement properties of PROMs measuring physical functioning in adults with type 2 diabetes. The Dependence/Daily Life subscale of the Diabetic Foot Ulcer Scale—Short Form (DFS-SF) and the Impact of Weight on Activities of Daily Living Questionnaire (IWADL) were most extensively evaluated. Both had sufficient ratings for aspects of content validity, although mostly with very low-quality evidence. Sufficient ratings for structural validity, internal consistency, and reliability were also found for both instruments, but responsiveness was rated inconsistent for both instruments. The other PROMs or subscales often had insufficient aspects of content validity, or their unidimensionality could not be confirmed.

Discussion: This systematic review showed that the Dependence/Daily Life subscale of the DFS-SF and the IWADL could be used to measure physical functioning in people with type 2 diabetes in research or clinical practice, while keeping the limitations of these instruments in mind. The measurement properties that have not been evaluated extensively for these PROMs should be evaluated in future studies. High risk of bias was found for many of the included studies, especially for the measurement properties content validity, structural validity, and reliability, leading to more uncertainty in the body of evidence.

Registration: The study protocol was registered in the PROSPERO database, number CRD42021234890.

Funding: No specific funding was received for this research.

Plain language summary

Plain language summary

Item #3: If allowed by the journal, provide a plain language summary with background information and key findings.

Explanation: Reports on technical topics, such as systematic reviews on the measurement properties of OMIs, are often difficult to understand by patients, clinicians, and researchers outside the field. A plain language summary is a very efficient way of conveying the essence of a report briefly and clearly.[48] Where requested or permitted by the journal, authors should endeavor to provide plain language summaries to further the reach and impact of their findings. The process of writing a good plain language summary can help the authors improve the overall clarity of their report. Plain language summaries should be written at maximum at a Grade 9 (Flesch-Kincaid grade level) reading comprehension level (Flesh reading ease score 70.0-60.0) and be intended for a variety of audiences, including patients and members of the public. If patients or members of the public are co-authors, they can check the clarity of the plain language summary. A readability analyzer can also be used (e.g., https://datayze.com/readability-analyzer). If a plain language summary after the regular abstract is not allowed by a journal, plain language summaries may be included as supplementary material.

Essential elements

- Provide a short paragraph outlining the content of the report, using short sentences, aimed at non-specialists in the field and written at maximum Grade 9 level in a way that they can easily understand.[48,49]
- If a technical term must be used, provide a description using simple language.[48,49]
- The structure should answer the main questions of "who/what/where/when/how many/why?" in a concise manner.[48,49]
- Provide a final sentence that explains why the research is important, and what the article has concluded.[48,49]

Example of item #3

Example 1: "Exercise has long been recognized as an important feature of eating disorders. Research has consistently found that many people with eating disorders exercise because they feel a drive to exercise, or in order to regulate their emotions. This type of exercise, called 'compulsive exercise' can have a detrimental impact on peoples' health and well-being. Compulsive exercise in eating disorders has been found to be associated with a range of adverse outcomes such as longer hospitalization, higher risk of relapse, and higher risk of a chronic outcome. In order to treat exercise as a symptom of eating disorders, clinicians need a way to measure exercise behaviors specific to eating disorders. There are a number of tests that measure exercise behaviors, however most of them were not designed for the needs of eating disorder patients. The current review therefore examines the literature in order to identify and assess measurement tools for patients with eating disorders."[50]

Example 2: "Bone fractures of the lower extremities are a common injury. During rehabilitation it is essential to evaluate how patients experience their physical functioning, in order to monitor the progress and to optimize treatment. To measure physical functioning often questionnaires (also

known as Patient Reported Outcome Measures) are used, such as the Lower Extremity Functional Scale (LEFS). However, it is not clear if the LEFS actually measures physical function, and if its other measurement properties are sufficient for using this questionnaire among patients with fractures in the lower extremities. Therefore, we systematically searched and assessed scientific papers on the development of the LEFS (i.e., its ability to measure physical functioning), and papers on the performance of the LEFS with regard to several measurement properties to identify possible factors that may cause measurement errors. Hereby we have assessed the quality of the studies included. Our main finding was that the LEFS may not measure all aspects of physical function. Given the low quality of the papers included in our study, these findings come with considerable uncertainty. As the LEFS was developed more than 20 years ago, it may not represent physical functioning as we currently conceptualize this. Therefore, we recommend to perform a study in which the content of the LEFS will be evaluated by experts in the field as well as patients, and modify the questionnaire as needed."[28]

Open Science

Registration and protocol

Item #4a: Provide registration information for the review, including register name and registration number, or state that the review was not registered.

Explanation: Stating where the systematic review was registered (such as PROSPERO, Open Science Framework) and the registration number or digital object identifier (DOI) for the register entry facilitates identification of the systematic review in the register.[2] This allows readers to compare what was pre-specified with what was eventually reported in the review and decide if any deviations may have introduced bias.[2] Reporting registration information also facilitates linking of publications related to the same systematic review (such as when a review is presented at a conference and published in a journal).[2,51]

Essential elements

- Provide registration information for the review, including register name and registration number, or state that the review was not registered.[2]

Example of item #24a

"This systematic review was registered in PROSPERO (Registration Number: CRD42020171591) […]."[32]

Item #4b: Indicate where the review protocol can be accessed, or state that a protocol was not prepared.

Explanation: A review protocol is distinct from a register entry for a review.[2] Although register entries (e.g., in PROSPERO) require information that might be included in the protocol, a review protocol is more extensive. Comparison of the methods pre-specified in the review protocol with what was eventually done allows readers to assess whether any deviations may have introduced bias.[2] The protocol may also contain information about the methods used that is not provided in the final review report.[2] Providing a citation, DOI, or link to the review protocol allows readers to locate the protocol more easily.[2] If the review protocol was not published or deposited in a public repository, or uploaded as a supplementary file to the review report, providing the contact details of the author responsible for sharing the protocol is recommended.[2] If authors did not prepare a review protocol, or prepared one but are not willing to make it accessible, this should be stated to prevent users spending time trying to locate the document.[2]

Essential elements

- Indicate where the review protocol can be accessed (such as by providing a citation, DOI, or link), or state that a protocol was not prepared.[2]

Example of item #4b

Example 1: "This systematic review was conducted and reported according to a registered and published protocol (PROSPERO registration number: CRD42016035554) (See S1 Text. Review Protocol) [citation to protocol provided] […]."[44]

Example 2: "This review was conducted according to an *a priori* published protocol [citation to protocol provided]."[52]

Item #4c: Describe and explain any amendments to information provided at registration or in the protocol.

Explanation: Careful consideration of a review's methodological and analytical approach early on is likely to lessen unnecessary changes after protocol development.[2,53] However, it is difficult to anticipate all scenarios that will arise, necessitating some clarifications, modifications, and changes to the protocol (such as data available may not be amenable to the planned synthesis).[2,54,55] For transparency, authors should report details of any amendments.[2] It might also be helpful to report if there were no amendments from the protocol. Amendments could be recorded in various places, including the full text of the review, a supplementary file, or as amendments to the published protocol or registration record.[2]

Essential elements

- Report details of any amendments to information provided at registration or in the protocol, noting: (a) the amendment itself, (b) the reason for the amendment, and (c) the stage of the review process at which the amendment was implemented.[2]

Example of item #4c

Example 1: "The protocol is available through ResearchGate [citation to protocol provided]. There were no deviations from the protocol […]."[45]

Example 2: "The objectives section has been revised, compared to the a priori protocol, to provide more clarity without changing the overall objectives of the review. […] Further inclusion criteria were added while identifying and screening the literature to complement those of the a priori protocol: Instruments needed to be multidimensional (e.g., include more dimensions than only information needs). […] In contrast to the a priori protocol, Embase was searched as recommended by COSMIN, and CINAHL was searched instead of OVID Nursing."[52]

Support

Item #5: Describe sources of financial or non-financial support for the review, and the role of the funders in the review.

Explanation: As with any research report, authors should be transparent about the sources of support received to conduct the review.[2] For example, funders may provide salary to researchers to undertake the review, or access to commercial databases that would otherwise not have been available.[2] Authors may have also obtained support from a translation service to translate articles, used the services of an information specialist to conduct searches, or in-kind use of software to manage or analyze the study data.[2] In some reviews, the funder may have contributed to defining the review question, determining eligibility of studies, collecting data, analyzing data, interpreting results, or approving the final review report.[2] There is potential for bias in the review findings arising from such involvement, particularly when the funder has an interest in obtaining a particular result.[2,56]

Essential elements

- Describe sources of financial or non-financial support for the review, specifying relevant grant ID numbers for each funder. If no specific financial or non-financial support was received, this should be stated.[2]
- Describe the role of the funders in the review. If funders had no role in the review, this should be declared – for example, by stating, "The funders had no role in the design of the review, data collection and analysis, decision to publish, or preparation of the manuscript."[2]

Example of item #5

Example 1: "The authors have not declared a specific grant for this research from any funding agency in the public, commercial or not-for-profit sectors."[18]

Example 2: "The first author is supported by a doctoral scholarship from Deakin University Faculty of Health, Australia. Author 2 is funded by an Alfred Deakin Postdoctoral Fellowship. Author 3 is supported by a Leadership Level 2 Fellowship, National Health and Medical Research Council (APP 1176885). Author 6 is a recipient of a doctoral scholarship from Coventry University, United Kingdom. These funders had no role in the design of this study, execution, analyses, and interpretation of the data, or involvement in the writing and decision to submit the manuscript."[24]

Competing interests

Item #6: Declare any competing interests of review authors.

Explanation: Authors of a systematic review may have been involved in the development, validation, or dissemination of one or several of the reviewed OMIs. They may also have relationships with organizations or entities with an interest in the review findings (for example, an author may serve as a consultant for a company or organization distributing the OMI under review). This may lead to bias in study evaluations and favorable conclusions about the studies or OMIs pertaining to the review author. Such relationships or activities are examples of a competing interest (or conflict of interest), which can negatively affect the integrity and credibility of systematic reviews.[2] Information about authors' relationships or activities that readers could consider pertinent or to have influenced the review should be disclosed using the format requested by the publishing entity (such as using the International Committee of Medical Journal Editors (ICMJE) disclosure form).[2,57] Authors should report how competing interests were managed for particular review processes. For example, if a review author was an author of an included study, they may have been prevented from assessing the risk of bias in the study results.[2]

Essential elements

- Disclose any of the authors' relationships or activities that readers could consider pertinent or to have influenced the review, such as being involved in the development, validation or dissemination of one of the reviewed OMIs.[2]
- If any authors had competing interests, report how they were managed for particular review processes, such as not being involved in the assessment of the OMI.[2]

Example of item #6

Example 1: "AI and BB declare that they have no competing interests. PW and PK are authors on some of the included articles. They were not involved in assessing the methodological quality of these articles. They have no other competing interests."[37]

Example 2: "Author ZZ was co-author on one of the included PROM development papers [citation provided]. She was not involved in any of the ratings of this paper."[19]

Availability of data, code, and other materials

Item #7: Report which of the following are publicly available and where they can be found: template data collection forms; data extracted from included studies; data used for all analyses; analytic code; any other materials used in the review.

Explanation: Sharing of data, analytic code, and other materials enables others to reuse the data, check the data for errors, attempt to reproduce the findings, and understand more about the analysis than may be provided by descriptions of methods.[2,58,59] Support for sharing of data, analytic code, and other materials is growing, including from journal editors.[2] Sharing of data, analytic code, and other materials relevant to a systematic review includes making them publicly available, such as all data extracted from included studies and a file indicating necessary data conversions[2]. Other materials might include a list of all references screened and any decisions about eligibility.[2]

Because sharing of data, analytic code, and other materials is not yet universal in health and medical research, even interested authors may not know how to make their materials publicly available.[2] Data, analytic code, and other materials can be included in the supplementary materials or uploaded to one of several publicly accessible repositories (such as Open Science Framework, Dryad, figshare).[2] The Systematic Review Data Repository (https://srdr.ahrq.gov) is another example of a platform for sharing materials specific to the systematic review community.[2,60] All of these open repositories should be given consideration, particularly if the completed review is to be considered for publication in a paywalled journal.[2] The Findable, Accessible, Interoperable, Reusable (FAIR) data principles are also a useful resource for authors to consult,[61] as they provide guidance on the best way to share information.[2] There are some situations where authors might not be able to share review materials, such as when the review team are custodians rather than owners of the data, or when there are legal or licensing restrictions.[2]

Essential elements

- Report which of the following are publicly available: template data collection forms; data extracted from included studies; data used for all analyses; analytic code; any other materials used in the review.[2]
- If any of the above materials are publicly available, report where they can be found (such as provide a link to files deposited in a public repository).[2]
- If data, analytic code, or other materials will be made available upon request, provide the contact details of the author responsible for sharing the materials and describe the circumstances under which such materials will be shared.[2]

Example of item #7

Example 1: " All data relevant to the study are included in the article or uploaded as supplementary information."[18]

Example 2: "All data generated and analyzed in this review are included in the articles."[32]

Example 3: "All relevant data are within the paper and its Supporting Information files."[44]

Introduction

Rationale

Item #8: Describe the rationale for the review in the context of existing knowledge.

Explanation: Systematic reviews of OMIs can provide a comprehensive overview of the measurement properties of the OMIs included. The rationale for wanting to have such an overview is often twofold: either to select the most suitable OMI for a particular use (e.g., for use as a primary outcome in research, for use in clinical practice, or for inclusion in a core outcome set), or to identify gaps in knowledge about the measurement properties of included OMIs. Describing the rationale should help readers understand why the review was conducted and what the review might add to existing knowledge.[2] Here, authors might also detail the outcome domain of interest for their review, their understanding of the outcome, and why this outcome is important to patients. For some outcome domains and populations, numerous systematic reviews have been conducted. Conducting additional systematic reviews could be redundant and might be wasting resources. If other systematic reviews or overviews addressing the same (or a largely similar) question are available, explanations should be given why the current review was considered necessary. This could for example be because previous reviews are out of date or have produced discordant results; new review methods are available to address the review question; existing reviews are methodologically flawed; or the current review was commissioned to inform a guideline or policy for a particular organisation.[2]

Essential elements

- Describe the current state of knowledge and its uncertainties.[2]
- Articulate why it is important to do the review.[2]
- If other systematic reviews or overviews addressing the same (or a largely similar) question are available, explain why the current review was considered necessary. If the review is an update or replication of a particular systematic review, indicate this and cite the previous review.[2]

Additional elements

- Consider elaborating on the outcome domain of interest and why this outcome is important to patients.

Example of item #8

Example 1: "Many trials in aged care in the acute hospital setting have been confounded by inadequate physical outcomes measures. The importance of measures of physical ability across the spectrum of ability has been argued by those prescribing exercise for older people. Pressure on already limited healthcare resources is predicted to increase as the average population age rises. An outcome measure that can accurately measure mobility is required to identify interventions that optimize physical outcomes of hospitalized older patients and facilitate effective targeting of healthcare services.

When selecting an outcome measure for a particular clinical purpose, there are many factors to consider. No systematic review assists clinicians to determine the most appropriate mobility outcome measure for older general medical patients in the acute care setting."[62]

Example 2: "[…] a variety of cancer-specific self-efficacy measures have been developed and validated. To ensure robust application of any instrument, a clearly delineated developmental process (e.g., definition of measurement aim, target population, item identification and selection) and critical validation (e.g., characterization of reliability and validity) are required. Not knowing whether existing instruments fulfill these quality criteria complicates comparison and selection. To the best of our knowledge, only one systematic review has been published on this subject, which focused exclusively on self-efficacy instruments developed for chronic diseases, such as asthma, arthritis, heart failure, and chronic obstructive pulmonary disease, and did not include cancer."[63]

Objectives

Item #9: Provide an explicit statement of the objective(s) or question(s) the review addresses and include as applicable the following (in any order): outcome domain of interest, population of interest, name/type of OMIs of interest, and measurement properties of interest.

Explanation: An explicit and concise statement of the review objective(s) or question(s) will help readers understand the scope of the review and assess whether the methods used in the review (such as eligibility criteria, search methods, data items, and synthesis) adequately address the objective(s).[2] Such statements may be written in the form of aims or objectives ("to examine the measurement properties of …") or as questions ("what is the quality of…?", "what are the measurement properties of...?").[2,26] The objective or question that the systematic review addresses often includes four key elements: the outcome domain, population, name or type of OMI, and the measurement properties.[7] It is therefore recommended to include these four key elements in the objective(s) or question(s) the review addresses, unless certain key elements are clearly irrelevant or redundant. For example, if the objective of the review is to evaluate the measurement properties of a certain OMI in a specific population, it might be irrelevant to include the outcome if that is clear from the name of the OMI. If multiple measurement properties are evaluated in the review, authors can state "measurement properties" or "quality" instead of listing each of the measurement properties. If multiple OMIs are evaluated in the review, authors can state the type of OMI (for example patient-reported outcome measures (PROMs) or performance-based tests). If different types of OMIs are evaluated in the review, authors can state "outcome measurement instruments".

The objective or question could also be linked to the rationale for the systematic review, for example, to identify gaps in knowledge in the measurement properties of available OMIs or to select the most suitable OMI for a particular use (e.g., in a core outcome set or a clinical trial). Depending on the rationale for the systematic review, providing information on interpretability and feasibility aspects of included OMIs might be a secondary objective. This is often most relevant if the rationale for the review is to select the most suitable OMI for a particular use.

Essential elements

- Provide an explicit statement of all objective(s) or question(s) the review addresses.[2]
- Use the four key elements (outcome domain, population, name or type of OMI and the measurement properties of interest) as applicable to formulate the objective(s) or question(s).[7]

Additional elements

- Consider linking the main objective(s) or question(s) to the rationale for the review (for example, to identify gaps in knowledge in the measurement properties of available OMIs or to select the most suitable OMI for a particular use).
- If information on feasibility and interpretability aspects is being provided, consider specifying this as a secondary objective.

Example of item #9

Example 1: "Therefore, this study aims to systematically assess the measurement properties of diabetes-specific PROMs [patient-reported outcome measure] for measuring physical functioning in

adults with type 2 diabetes to make recommendations on the most suitable PROM to use in research or clinical practice."[18]

Example 2: "The aim of the present study was to systematically evaluate the content validity of PROMs, which have specifically been developed to measure (aspects of) HRQOL [health-related quality of life] in people with type 2 diabetes."[19]

Example 3: "The aim of this study was to systematically review the content validity and measurement properties of all PF [physical function] scales that have been validated for use in patients with RA [rheumatoid arthritis], by linking their content to the ICF [International Classification of Functioning, Disability and Health] and to appraise the currently available evidence of the quality of their measurement properties in order to offer recommendations for the use of PF scales for various purposes and settings."[39]

Example 4: "Therefore, this study aimed to systematically review the literature to evaluate the content validity and other measurement properties of the *LEFS* in patients with fractures of the lower extremities […]."[28]

Methods

Followed guidelines

Item #10: Specify, with references, the methodology and/or guidelines used to conduct the systematic review.

Explanation: Different methodologies and guidelines are available (and regularly updated) that guide the overall process of conducting a systematic review of OMIs, such as the COSMIN guideline for systematic reviews,[7] OMERACT filter 2.1[64] or 2.2,[65] or the JBI Manual for Evidence Synthesis.[66] The most recent versions of these methodologies and guidelines can be found on the websites of the respective organizations (www.cosmin.nl; www.omeract.org; www.jbi.global). Specifying the methodology and/or guidelines used and being specific about the versions and checklists used within those guidelines by providing references, allows readers to determine whether the study was conducted following established guidance and used high quality methods.

Essential elements

- Provide an explicit statement of the methodology and/or guidelines used to conduct the systematic review.
- Provide a citation for each (version of the) methodology/and or guidelines used.

Example of item #10

Example 1: "In conducting this systematic review, the updated Consensus-based Standards for selection of health Measurement INstruments (COSMIN) methodology for systematic reviews of PROMs was used [references provided to the COSMIN guideline for systematic reviews of PROMs, the COSMIN risk of bias checklist, and the COSMIN methodology for content validity]."[36]

Example 2: "Physical function was the first core outcome domain for which candidate instruments Were evaluated through the OMERACT Filter 2.1 using the OMERACT Instrument Selection Workbook templates [references provided to the OMERACT Filter 2.1, the elaboration of the OMERACT Filter 2.1, and the OMERACT Handbook]."[35]

Eligibility criteria

Item #11: Specify the inclusion and exclusion criteria for the review.

Explanation: Specifying the criteria used to decide what evidence was eligible in sufficient detail should enable readers to understand the scope of the review and verify inclusion decisions.[2,67] The inclusion and exclusion criteria often relate to the four key elements: the outcome domain, population, name or type of OMI, and measurement properties.[7] Definitions for these elements should be provided. For measurement properties, it is important that review authors state the dictionary/taxonomy used (e.g., the COSMIN taxonomy[12]), as authors of primary studies often differ in terminology used for measurement properties. In a review examining the measurement properties of the Dutch-Flemish PROMIS (Patient-Reported Outcomes Measurement Information System) physical function item bank and instruments, the authors therefore stated that they used the terminology from the COSMIN taxonomy in their assessment of measurement properties.[37] For a study to be included, often an aim should be to evaluate one or more of the measurement properties of interest, report on the development of an OMI, or report on its interpretability and feasibility aspects.[7] Other inclusion and exclusion criteria can relate to the language and publication status of study reports included in the review.

Essential elements

- Specify all study characteristics used to decide whether a study was eligible for inclusion in the review, which can include the outcome domain, population, name/type of OMI, and/or measurement properties of interest,[7] as well as other characteristics, such as eligible study design(s) (e.g., should an aim of the study be the development or validation of an OMI, or are studies in which an OMIs is used also included) and setting(s).
- Specify eligibility criteria with regard to report characteristics, such as year of dissemination, language, and report status (for example, whether reports such as unpublished manuscripts and conference abstracts were eligible for inclusion).[2]
- Provide rationales for any notable restrictions to study eligibility.[2] For example, authors might explain that the review was restricted to studies published from 2015 onwards because that was the year the OMI was first available.

Example of item #11

Example 1: "PROMs that were considered to measure physical functioning based on the Wilson and Cleary model in the first review were included in the current study when the following criteria were met:

1. Construct of interest: The PROM [patient-reported outcome measure] or a relevant sub-scale of a PROM should measure physical functioning. We adopted the definition of the Patient-Reported Outcomes Measurement Information System (PROMIS), a large US initiative that developed generic PROMs for core health outcomes, which defined physical functioning as the capability to perform physical activities (i.e., what a person can do in the daily environment), rather than performance (i.e., what a person actually does) or capacity (i.e., what a person can do in a standardized-controlled environment, often measured by performance-based tests). Capability to perform physical activities includes the functioning of one's upper extremities (dexterity), lower extremities (walking or mobility), and central regions (neck, back), as well as instrumental activities of daily living, such as running errands. In case a subscale of the instrument measures physical functioning, only that subscale was included.

2. Population: At least 50% of the study population or reported subgroups should consist of adults with type 2 diabetes mellitus.
3. Instrument type: The instrument should be a questionnaire, to be completed by the person with type 2 diabetes in self-report or interview form.
4. Measurement properties: At least one of the aims of the paper should be the development of a diabetes-specific PROM or the evaluation of one or more measurement properties of a diabetes-specific PROM. Studies that aim to evaluate the interpretability of a PROM were also included. Studies that use a PROM but do not intend to evaluate its measurement properties or in which the PROM is only used as a comparison instrument in the validation of another instrument were excluded."[18]

Example 2: "Original studies reporting the development and/or validation of pain scoring instruments in farm animals as well as manuscripts reporting the assessment of one or more measurement properties of these instruments, were included. These studies involved naturally-occurring or experimental acute and chronic painful conditions in bovine (beef and dairy cattle, and buffalo), ovine (sheep and lamb), caprine (goat and kid), camel, porcine (pig and piglets) and poultry (chicken, fowl, ducks, turkeys and geese). These species were chosen since they are the most relevant species used for production of animal protein (meat, dairy products and eggs) according to the Organization for Economic Co-operation and Development (OECD) and the Food and Agriculture Organization (FAO) of the United Nations, the OECD-FAO Agricultural Outlook 2020–2029.

Studies that only reported the use of pain scales as an OMI (e.g., in randomized controlled trials comparing two different treatments), studies in which a pain scale was used in the validation of another instrument, studies reporting only ethogram/list of pain-related behaviors without a scoring system, studies reporting non-ordinal pain assessment variables, or review and systematic reviews were not included. Studies reporting the use of pain scoring instruments to measure constructs other than pain, for example studies assessing animal welfare, in which pain was considered within the overall evaluation, studies assessing nociceptive testing, and studies for which the full text was not available were excluded."[68]

Information sources

Item #12: Specify all databases, registers, preprint servers, websites, organizations, reference lists and other sources searched or consulted to identify studies. Specify the date when each source was last searched or consulted.

Explanation: Authors should provide a detailed description of the information sources, such as bibliographic databases, registers, preprint servers, websites, organizations, reference lists and other sources that were searched or consulted, including the dates when each source was last searched, to allow readers to assess the completeness and currency of the systematic review, and allow updating.[2,69] Authors should fully report the "what, when, and how" of the sources searched; the "what" and "when" are covered in item #12, and the "how" is covered in item #13.[2] Further guidance and examples about searching can be found in PRISMA-Search, an extension to the PRISMA statement for reporting literature searches in systematic reviews.[2,70]

Besides the studies described in reports, a copy of the OMI, a user/scoring manual and/or a measurement protocol may also need to be retrieved to evaluate the OMI (for example to assess content validity). Therefore, authors should also state the sources searched or consulted to retrieve OMI(s), user/scoring manual(s), and/or measurement protocol(s). Review authors should also state what was done if they could not obtain OMIs, user/scoring manuals, and/or measurement protocols (in the required language). For example, would content validity then be assessed based on what is described in the reports, not be assessed at all, or would they exclude the OMI from the review.

Essential elements

- Specify the date when each source (such as database, register, website, organization) was last searched or consulted.[2]
- If bibliographic databases were searched, specify for each database its name (such as MEDLINE, CINAHL), the interface or platform through which the database was searched (such as Ovid, EBSCOhost), and the dates of coverage (where this information is provided).[2]
- If study registries (such as PROSPERO) and other online repositories (such as COSMIN, PROMIS, COMET, PROQUEST or PROQOLID) were searched, specify the name of each source and any restrictions that were applied.[2]
- If preprint servers were searched, specify the name of each source and any restrictions that were applied.
- If websites, search engines, or other online sources were browsed or searched, specify the name and URL (uniform resource locator) of each source.[2]
- If information pertaining to OMIs (such as a copy of the OMI, user/scoring manuals, measurement protocols) was searched, specify each source and when it was consulted, and any restrictions that were applied.
- If information pertaining to OMIs could not be obtained, describe how this was dealt with.
- If organizations or manufacturers were contacted to identify studies or information pertaining to OMIs (such as a copy of the OMI, user/scoring manuals, measurement protocols), specify the name of each source.[2]
- If individuals were contacted to identify studies or information pertaining to OMIs (such as a copy of the OMI, user/scoring manuals, measurement protocols), specify the types of

individuals contacted (such as developers of OMIs, authors of studies included in the review, or researchers with expertise in measurement).[2]

- If reference lists were examined, specify the types of references examined (such as references cited in study reports included in the systematic review, or references cited in systematic review reports on the same or a similar topic).[2]
- If cited or citing reference searches (also called backward and forward citation searching) were conducted, specify the bibliographic details of the reports to which citation searching was applied, the citation index or platform used (such as Web of Science), and the date the citation searching was done.[2]
- If journals or conference proceedings were consulted, specify the name of each source, the dates covered and how they were searched (such as handsearching or browsing online).[2]

Example of item #12

Example 1: "A comprehensive search was performed in the bibliographic databases MEDLINE (through PubMed) and EMBASE (through www.embase.com) from inception up to January 1, 2022 without language restrictions. […] Reference lists of included articles were searched by hand to ensure all relevant studies and available translations were considered. […] PROMs [patient-reported outcome measures] and manuals were retrieved by searching Google or by contacting PROM developers."[18]

Example 2: "Electronic databases

The electronic databases searched for the systematic review are outlined in Table 1. All databases were searched from inception.

Additional searches

Following recognized approaches, we searched Google Scholar (last searched 5th July 2021) with the names of the instruments identified in the database searches and taken forward for review in order to identify potential development papers for assessing content validity. The first 100 hits on Google Scholar were screened for inclusion. Where development papers were not found in this manner, manual searching of instrument citations in the included papers was conducted. In addition, citation tracking, by means of screening of references (via Scopus) and Google Scholar citations, was conducted on full text research articles (not development papers) meeting the eligibility criteria at Stage 2 (last searched 5th July 2021), as a supplementary measure to identify any additional studies not captured by the database searching."[41]

The following is a reproduced version of Table 1 in the review by Carlton et al., 2022.[41]

Table 1 Electronic databases for the primary searches

Host	Database	Dates covered	Date searched (Stage 1)	Date searched (Stage 2)
Ovid	Ovid MEDLINE(R) and Epub Ahead of Print, In-Process & Other Non-Indexed Citations, Daily and Versions(R)	1946 to Present	8th July 2020	2nd November 2020
Ovid	Embase	1974 to Present	8th July 2020	2nd November 2020
Wiley	Cochrane Database of Systematic Reviews (Cochrane Library)	CDSR 1996 to Present	8th July 2020	2nd November 2020
Wiley	Cochrane Central Register of Controlled Trials (Cochrane Library)	CENTRAL 1898 to Present	8th July 2020	2nd November 2020
EBSCO	CINAHL	1974 to Present	9th July 2020	2nd November 2020
Ovid	PsycINFO	1806 to Present	8th July 2020	2nd November 2020

Search strategy

Item #13: Present the full search strategies for all databases, registers, and websites, including any filters and limits used.

Explanation: Reporting the full details of all search strategies (such as the full, line by line search strategy as run in each database) enhances the transparency of the systematic review, improve replicability, and enable a review to be more easily updated.[2,69,71] Presenting only one search strategy from among several hinders the readers' ability to assess how comprehensive the searchers were and does not provide them with the opportunity to detect any errors.[2] Furthermore, making only one search strategy available limits replication or updating of the searches in the other databases, as the search strategies would need to be reconstructed through adaptation of the one(s) made available.[2] As well as reporting the full search strategies, which is often included in the supplementary files, a description of the conceptual structure of the search strategy in relation to the research question can help readers understand whether search terms were included for the population, outcome domain, OMI of interest and measurement properties, and how these search terms were linked. Additionally, a description of the search strategy development process can help readers judge to what extent the strategy is likely to have identified all studies relevant to the review's inclusion criteria.[2] The description of the search strategy development process might include details of the approaches used to identify keywords, synonyms, or subject indexing terms used in the search strategies, or any processes used to validate or peer review the search strategies, for example by consulting a medical information specialist.[2] Empirical evidence suggests that peer review of search strategies is associated with improvements to search strategies, leading to retrieval of additional relevant records.[2,72] Further guidance and examples of reporting search strategies can be found in PRISMA-Search.[2,70]

Methodological search filters have been developed to support the development of search strategies for systematic reviews of OMIs and their measurement properties. For example, a validated search filter exists to find studies on measurement properties of OMIs.[73] Use of such filters can help to improve the efficiency of search strategies. Other filters which speak to, for example, the type of OMI (for example, the PROM Group Construct and Instrument Type filters)[74] are available as well, as are websites where you can find search filters (for example blocks.bmi-online.nl).

Essential elements

- Provide the full line by line search strategy as run in each database with a sophisticated interface (such as Ovid), or the sequence of terms that were used to search simpler interfaces, such as search engines or websites.[2] This can be included in the supplementary files.
- Describe any limits applied to the search strategy (such as date or language) and justify these by linking back to the review's eligibility criteria.[2]
- Describe the conceptual structure of the search strategy in relation to the research question. Specify all components (such as the outcome domain, population, name/type of OMI, and measurement properties of interest), how these components were linked, and describe omissions or adaptations to any element.[7]
- If published approaches such as search filters designed to retrieve specific types of records (for example, search filter for measurement properties)[73], or search strategies from other

systematic reviews were used, cite them. If published approaches were adapted – for example if existing search filters were amended – note the changes made.[2]

- If an information specialist or librarian was involved in developing the search strategy, report this.
- If natural language processing or text frequency analysis tools were used to identify or refine keywords, synonyms or subject indexing terms to use in the search strategy,[75,76] specify the tool(s) used.[2]
- If a tool was used to automatically translate search strings for one database to another,[77] specify the tool used.[2]
- If the search strategy was validated – for example, by evaluating whether it could identify a set of clearly eligible studies – report the validation process used and specify which studies were included in the validation set.[2,69]
- If the search strategy was peer reviewed, report the peer review process used and specify any tools used (such as the Peer Review of Electronic Search Strategies (PRESS) checklist).[2,78]

Example of item #13

Example 1: “The search consisted of three elements: (1) type 2 diabetes, using a comprehensive set of search terms from a clinical librarian of the Vrije Universiteit Amsterdam, the Netherlands; (2) PROMs [patient-reported outcome measures], using a PROM filter; and (3) measurement properties, using a modified version of the measurement properties filter. No search terms were used for the construct, as the complete series of reviews intended to find all instruments that have been validated in people with type 2 diabetes. Moreover, for this specific review, we intended to also include physical functioning subscales of PROMs measuring broader constructs, such as quality of life. Adding search terms for physical functioning could have prevented finding these broader instruments as subscales are not always mentioned in the abstract. The complete search strategy can be found in online supplemental appendix 2.”[18]

The following is an abridged version of Appendix 2 in the review by Elsman et al., 2022.[18]

Appendix 2. Search strategy

PUBMED search January 1, 2022

#1 Diabetes type 2

((Diabet*[tiab] AND (("non insulin"[tiab] AND depend*[tiab]) OR ("noninsulin"[tiab] AND depend*[tiab]) OR “type 2”[tiab] OR “type II” [tiab])) OR iddm[tiab] OR niddm[tiab] OR “glucose intolerance”[tiab] OR “insulin resistant”[tiab] OR “insulin resistance”[tiab])

#2 Modified filter for studies on measurement properties*

~~instrumentation[sh] OR methods[sh] OR~~ "Validation Studies"[pt] ~~OR "Comparative Study"[pt]~~ OR "psychometrics"[MeSH] OR psychometr*[tiab] OR clinimetr*[tw] OR clinometr*[tw] OR "outcome assessment (health care)"[MeSH] OR "outcome assessment"[tiab] OR "outcome measure*"[tw] OR "observer variation"[MeSH] OR "observer variation"[tiab] ~~OR "Health Status Indicators"[Mesh]~~ OR "reproducibility of results"[MeSH] OR reproducib*[tiab] OR "discriminant analysis"[MeSH] OR reliab*[tiab] OR unreliab*[tiab] OR valid*[tiab] OR "coefficient of variation"[tiab] ~~OR coefficient[tiab]~~ OR homogeneity[tiab] OR homogeneous[tiab] OR "internal consistency"[tiab] OR (cronbach*[tiab] AND (alpha[tiab] OR alphas[tiab])) OR (item[tiab] AND (correlation*[tiab] OR selection*[tiab] OR reduction*[tiab])) OR agreement[tw] OR precision[tw] OR imprecision[tw] OR "precise values"[tw] OR test-retest[tiab] OR (test[tiab] AND retest[tiab]) OR (reliab*[tiab] AND (test[tiab] OR retest[tiab])) OR stability[tiab] OR interrater[tiab] OR inter-rater[tiab] OR intrarater[tiab] OR intra-rater[tiab] OR intertester[tiab] OR inter-tester[tiab] OR intratester[tiab] OR intra-tester[tiab] OR interobserver[tiab] OR inter-observer[tiab] OR intraobserver[tiab] OR intra-observer[tiab] OR intertechnician[tiab] OR inter-technician[tiab] OR intratechnician[tiab] OR intra-technician[tiab] OR interexaminer[tiab] OR inter-examiner[tiab] OR intraexaminer[tiab] OR intra-examiner[tiab] OR interassay[tiab]

OR inter-assay[tiab] OR intraassay[tiab] OR intra-assay[tiab] OR interindividual[tiab] OR inter-individual[tiab] OR intraindividual[tiab] OR intra-individual[tiab] OR interparticipant[tiab] OR inter-participant[tiab] OR intraparticipant[tiab] OR intra-participant[tiab] OR kappa[tiab] OR kappa's[tiab] OR kappas[tiab] OR repeatab*[tw] OR ((replicab*[tw] OR repeated[tw]) AND (measure[tw] OR measures[tw] OR findings[tw] ~~OR result[tw] OR results[tw]~~ OR test[tw] OR tests[tw])) OR generaliza*[tiab] OR generalisa*[tiab] OR concordance[tiab] OR (intraclass[tiab] AND correlation*[tiab]) OR discriminative[tiab] OR "known group"[tiab] OR "factor analysis"[tiab] OR "factor analyses"[tiab] OR "factor structure"[tiab] OR "factor structures"[tiab] ~~OR dimension*[tiab]~~ OR subscale*[tiab] OR (multitrait[tiab] AND scaling[tiab] AND (analysis[tiab] OR analyses[tiab])) OR "item discriminant"[tiab] OR "interscale correlation*"[tiab] OR error[tiab] OR errors[tiab] OR "individual variability"[tiab] OR "interval variability"[tiab] OR "rate variability"[tiab] OR (variability[tiab] AND (analysis[tiab] OR values[tiab])) ~~OR (uncertainty[tiab] AND (measurement[tiab] OR measuring[tiab]))~~ OR "standard error of measurement"[tiab] ~~OR sensitiv*[tiab]~~ OR responsive*[tiab] OR (limit[tiab] AND detection[tiab]) OR "minimal detectable concentration"[tiab] OR interpretab*[tiab] OR ((minimal[tiab] OR minimally[tiab] OR clinical[tiab] OR clinically[tiab]) AND (important[tiab] ~~OR significant[tiab]~~ OR detectable[tiab]) AND (change[tiab] OR difference[tiab])) OR (small*[tiab] AND (real[tiab] OR detectable[tiab]) AND (change[tiab] OR difference[tiab])) OR "meaningful change"[tiab] OR "ceiling effect"[tiab] OR "floor effect"[tiab] OR "Item response model"[tiab] OR IRT[tiab] OR Rasch[tiab] OR "Differential item functioning"[tiab] OR DIF[tiab] OR "computer adaptive testing"[tiab] OR "item bank"[tiab] OR "cross-cultural equivalence"[tiab]

#3 PROM filter (developed by the University of Oxford, see www.comin.nl)

(HR-PRO[tiab] OR HRPRO[tiab] OR HRQL[tiab] OR HRQoL[tiab] OR QL[tiab] OR QoL[tiab] OR quality of life[tw] OR life quality[tw] OR health index*[tiab] OR health indices[tiab] OR health profile*[tiab] OR health status[tw] OR ((patient[tiab] OR self[tiab] OR child[tiab] OR parent[tiab] OR carer[tiab] OR proxy[tiab]) AND ((report[tiab] OR reported[tiab] OR reporting[tiab]) OR (rated[tiab] OR rating[tiab] OR ratings[tiab]) OR based[tiab] OR (assessed[tiab] OR assessment[tiab] OR assessments[tiab]))) OR ((disability[tiab] OR function[tiab] OR functional[tiab] OR functions[tiab] OR subjective[tiab] OR utility[tiab] OR utilities[tiab] OR wellbeing[tiab] OR well being[tiab]) AND (index[tiab] OR indices[tiab] OR instrument[tiab] OR instruments[tiab] OR measure[tiab] OR measures[tiab] OR questionnaire[tiab] OR questionnaires[tiab] OR profile[tiab] OR profiles[tiab] OR scale[tiab] OR scales[tiab] OR score[tiab] OR scores[tiab] OR status[tiab] OR survey[tiab] OR surveys[tiab])))

(#1 AND #2 AND #3) NOT ("addresses"[Publication Type] OR "biography"[Publication Type] OR "case reports"[Publication Type] OR "comment"[Publication Type] OR "directory"[Publication Type] OR "editorial"[Publication Type] OR "festschrift"[Publication Type] OR "interview"[Publication Type] OR "lectures"[Publication Type] OR "legal cases"[Publication Type] OR "legislation"[Publication Type] OR "letter"[Publication Type] OR "news"[Publication Type] OR "newspaper article"[Publication Type] OR "patient education handout"[Publication Type] OR "popular works"[Publication Type] OR "congresses"[Publication Type] OR "consensus development conference"[Publication Type] OR "consensus development conference, nih"[Publication Type] OR "practice guideline"[Publication Type]) NOT ("animals"[MeSH Terms] NOT "humans"[MeSH Terms])

* Modified from Terwee et al.[28] The crossed-out search terms were left out because these terms, in combination with the search terms for diabetes, yielded too many abstracts to read.

Example 2: "The search strategy was developed by an information librarian using a wide range of search terms for intellectual and developmental disabilities, MH [mental health] issues, children and adolescents, and psychometric properties. No limits were applied to the study design, language, or publication type. The search strategy was adapted to each database (see complete search strategies in Appendix II)."[30]

The following is an abridged version of Appendix II in the review by Halvorsen et al., 2023.[30]

Appendix II Search strategies

Date of search: 21st of February 2020

Date of updated search 13th of March 2021. The search was for the outcome measures included from the 2020 search, in Ovid databases limited to publication date since the first search.

Searches for ongoing and unpublished trials 16th of May 2021

Embase < 1980 to 2020 Week 07 > (Ovid interface)

#	Searches
1	mental deficiency/or mental retardation malformation syndrome/or down syndrome/or de lange syndrome/or fragile x syndrome/or prader willi syndrome/or williams beuren syndrome/or x linked mental retardation/or wagr syndrome/or cat cry syndrome/or developmental disorder/or learning disorder/or language disability/
2	((intellectual* or mental* or developmental* or learning* or cognit*) adj3 (disab* or impair* or handicap* or disorder* or subnormal* or deficien* or difficult*)).ti,ab
3	(retard* or rett* or prader willi or fragile X or Crying cat or cri du chat or savants or William* syndrome* or (down* adj2 syndrome*)).ti,ab
4	1 or 2 or 3
5	mental disease/or comorbidity/or behavior disorder/or disruptive behavior/or problem behavior/or conduct disorder/or emotional disorder/
6	((mental* or emotional* or psych*) adj2 (disorder* or disturbance* or ill* or well-being or health* or disease* or abnormal* or patholog* or problem* or condition*)).ti,ab
7	((behavi* or conduct* or anger) adj3 (problem* or disorder*)).ti,ab
8	5 or 6 or 7
9	exp child/or exp adolescent/or exp adolescence/or exp childhood/or exp pediatrics/
10	(child* or kid or kids* or minors* or juvenil* or adoles* or youth* or youngster* or teen* or preteen* or boy or boys* or girl* or pediatr* or paediatr*).ti,ab,kw,hw,jx
11	9 or 10
12	4 and 8 and 11
13	psychologic assessment/or psychologic test/or mental test/or psychological interview/or psychological rating scale/or psychometry/or structured interview/
14	(psychometric* or instrument* or inventor* or self-report* or validat* or validity or reliab* or norm or norms or (measurement* adj tool*)).ti,ab
15	13 or 14
16	12 and 15

Searches for ongoing and unpublished trials

ClinicalTrials.gov

31 Studies found for: psychometric | Intellectual Disability OR mental health | Child (birth-17)

https://clinicaltrials.gov/ct2/results?cond=Intellectual+Disability+OR+mental+health&term=psychometric&cntry=&state=&city=&dist=&Search=Search&age=0

WHO International Clinical Trials Registry Platform (ICTRP)

https://apps.who.int/trialsearch/

intellect* OR mental*AND psyhometric* propertie* limited clinical trials in children

Selection process

Item #14: Specify the methods used to decide whether a study met the inclusion criteria of the review, e.g., including how many reviewers screened each record and each report retrieved, whether they worked independently, and if applicable, details of automation tools/AI used in the process.

Explanation: Study selection is typically a multi-stage process in which potentially eligible studies are first identified from screening titles and abstracts, then assessed through full text review, and, where necessary, contact with study investigators.[2] Increasingly, a mix of screening approaches might be applied (such as automation or artificial intelligence (AI) methods to eliminate records before screening or prioritize records during screening).[2] In addition to automation/AI, authors increasingly have access to screening decisions that are made by people independent of the author team (such as crowdsourcing).[2] Authors should describe in detail the process for deciding how records retrieved by the search were considered for inclusion in the review, to enable readers to assess the potential for errors in the selection.[2,79-82]

Essential elements – regardless of the selection processes used

- Report how many reviewers screened each record (title/abstract) and each report retrieved, whether multiple reviewers worked independently (that is, were unaware of each other's decisions) at each stage of screening or not (for example, records screened by one reviewer and exclusions verified by another), and any processes used to resolve disagreements between screeners (for example, referral to a third reviewer or by consensus).[2]
- Report any processes used to obtain or confirm relevant information from study investigators.[2]
- If only a subset of abstracts or articles was screened by a second reviewer, report the percentage specific agreement between the two reviewers.
- If abstracts or articles required translation into another language to determine their eligibility, report how these were translated (for example, by asking a native speaker or by using software programs).[2]

Essential elements – selection process with automation tools/AI

- Report how automation tools/AI were integrated within the overall study selection process; for example, whether records were excluded based solely on a machine assessment or whether machine assessments were used to double-check human decisions.[2]
- If an externally derived machine learning classifier was applied, either to eliminate records or to replace a single screener, include a reference or URL to the version used. If the classifier was used to eliminate records before screening, report the number eliminated in the PRISMA-COSMIN for OMIs 2024 flow diagram as "Records marked as ineligible by automation tools".[2]
- If an internally derived machine learning classifier was used to assist with the screening process, identify the software/classifier and version, describe how it was used (such as to remove records or replace a single screener) and trained (if relevant), and what internal or external validation was done to understand the risk of missed studies or incorrect classifications. For example, authors might state that the classifier was trained on the set of records generated for the review in question (as may be the case when updating reviews) and specify which thresholds were applied to remove records.[2]

- If machine learning algorithms were used to prioritize screening (whereby unscreened records are continually re-ordered based on screening decisions), state the software used and provide details of any screening rules applied (for example, screening stopped altogether leaving some records to be excluded based on automated assessment alone, or screening switched from double to single screening once a pre-specified number or proportion of consecutive records was eliminated).[2]

Essential elements – selection proceed with crowdsourcing or previous "known" assessments

- If crowdsourcing was used to screen records, provide details of the platform used and specify how it was integrated within the overall study selection process.[2]
- If datasets of already-screened records were used to eliminate records retrieved by the search from further consideration, briefly describe the derivation of these datasets. For example, if prior work has already determined that a given record does not meet the eligibility criteria, it can be removed without manual checking.[2]

Example of item #14

Example 1: "Each abstract or full-text paper was independently reviewed by two reviewers from the review team. If reviewers disagreed, they discussed the abstract or paper until consensus was reached or a third author with experience in systematic reviews of PROMs [patient-reported outcome measures] made the final decision."[19]

Example 2: "All titles and abstracts were independently screened by at least two reviewers in Covidence. All full-text papers were independently screened. Disagreements were resolved by discussion, and if needed, a third author was consulted to reach a final decision."[30]

Example 3: "Articles retrieved from the electronic search were imported into the EndNote reference program (Ver. 9.3.1). After removing duplicates, two reviewers independently screened the titles and the abstracts of all identified records, and evaluated the full texts of all potentially eligible articles. […] Any disagreements between the two reviewers were resolved by discussion with an expert researcher.[32]

Data collection process

Item #15: Specify the methods used to collect data from reports, e.g., including how many reviewers collected data from each report, whether they worked independently, any processes for obtaining or confirming data from study investigators, and if applicable, details of automation tools/AI used in the process.

Explanation: Authors should report the methods used to collect data from included reports, to enable readers to assess the potential for errors in the data presented.[2,83-85]

Essential elements

- Report how many reviewers collected data from each report, whether multiple reviewers worked independently or not (for example, data collected by one reviewer and checked by another),[86] and any processes used to resolve disagreements between data collectors.[2]
- Report any processes used to obtain or confirm relevant data from OMI developers or study investigators (such as how they were contacted, what data were sought, and success in obtaining the necessary information).[2]
- If any automation tools/AI were used to collect data, report how the tool was used (such as machine learning models to extract sentences from articles relevant to the characteristics of the population, OMI or measurement properties),[87,88] how the tool was trained, and what internal or external validation was done to understand the risk of incorrect extractions.[2]
- If articles required translation into another language to enable data collection, report how these articles were translated (for example, by asking a native speaker or by using software programs).[2,89]
- If any software was used to extract data from figures,[90] specify the software used.[2]
- If any decision rules were used to select data from multiple reports corresponding to a study, and any steps were taken to resolve inconsistencies across reports, report the rules and steps used.[2,91]

Additional elements

- If a published data extraction form was used (e.g., from another source/report, for example the data extraction tables in the COSMIN guideline), consider citing the source.

Example of item #15

Example 1: "For each included study, data were extracted independently by one reviewer. This was then verified for accuracy by a second reviewer. Where disagreements occurred, these were resolved through discussion. Data were extracted onto a bespoke data extraction table."[25]

Example 2: "Data extraction was undertaken independently by two reviewers using a pre-prepared data extraction sheet, with consensus reached through discussion. The data extraction sheet was first piloted (on two development paper articles and two measurement property articles), before being revised for further use. Extraction was informed by tools developed by COSMIN on reporting guidance: https://www.cosmin.nl/tools/guideline-conducting-systematic-review-outcome-measures/."[41]

Data items

Item #16: List and define which data were extracted (e.g., characteristics of study populations and OMIs, measurement properties' results, and aspects of feasibility and interpretability). Describe methods used to deal with any missing or unclear information.

Explanation: Authors should report the data and information extracted from each included report so that readers can understand the type of information sought and to inform data collection in other similar reviews.[2] Variables of interest might include characteristics of included study populations (e.g., country, setting, response rate, age, gender, and sex of sample; disease duration and severity if applicable), characteristics of included OMIs (e.g., construct of interest, mode of administration, recall period, scoring, language), information on feasibility (e.g., completion time, ease of administration, cost of OMI) and interpretability (e.g., floor/ceiling effects, change scores, minimal important change and difference, information on response shift) aspects, and the measurement properties' results (e.g., factor structures, Cronbach's alphas, correlation coefficients). For studies on responsiveness, authors may also collect data on characteristics of the interventions (such as what interventions were delivered, how they were delivered, by whom, where, and for how long).[2] If important information is missing, this information might be retrieved by contacting the study authors, or assumptions might be made about the missing or unclear information. For example, if a study was conducted in the U.S., and the language of the PROM was not specified, authors might assume that the language of the PROM was English.

Essential elements

- List and define all variables for which data were sought. It may be sufficient to report a brief summary of information collected if the data collection and dictionary forms are made available (for example, as additional files or deposited in a publicly available repository).[2]
- Describe methods used to deal with any missing or unclear information from the included studies.

Example of item #16

Example 1: "[…] data collection involved extracting information on the general characteristics of included studies as follows: (a) instrument, author(s) and year of publication; (b) general construct assessed; (c) APLF [Australian Physical Literacy Framework] domain(s) assessed; (d) targeted age group/grades; (e) sample population/country; (f) sample size, mean age, standard deviation; (g) instrument available translation; (h) completion time (minutes or seconds); (i) recall period; (j) tool sub-scale(s)/number of items; (k) response options; (l) psychometric properties evaluated/statistical tests utilized."[24]

Example 2: "The following data were extracted from the included articles: first author, year of publication, study participants, study setting, study design, study location, and the characteristics and psychometric properties of PROMs [patient-reported outcome measures]."[32]

Study risk of bias assessment

Item #17: Specify the methods used to assess risk of bias in the included studies, e.g., including details of the tool(s) used, how many reviewers assessed each study and whether they worked independently, and if applicable, details of automation tools/AI used in the process.

Explanation: Users of reviews need to know the risk of bias in the included studies to appropriately interpret the evidence.[2] Risk of bias refers to the potential for study findings to systematically deviate from the truth due to methodological flaws in the design, conduct or analysis.[14] Several (versions of) tools have been developed to assess study limitations for individual studies on measurement properties of OMIs.[92] The COSMIN Risk of Bias Checklist was specifically developed to assess the risk of bias in individual studies on measurement properties of OMIs.[15] It is the most detailed and widely used tool to assess risk of bias in individual studies on measurement properties, and was developed using a consensus-based process. Other tools, including a risk of bias tool for minimal important change (MIC),[93,94] also exist. Reporting details of the selected tool, such as its version and the scoring system used in the tool, enables readers to assess whether the tool was appropriate for identifying risk of bias. Reporting details of how studies were assessed (such as by one or two authors) allows readers to assess the potential for errors in the assessments.[2,83] If reviewers worked independently, it should be stated how discrepancies were resolved. Review authors should also report whether an overall risk of bias judgment per measurement property was made (for example the "worst score counts" method).[15]

Essential elements

- Specify the tool(s) (and version) used to assess risk of bias in the included studies.[2]
- Report whether an overall risk of bias judgment per measurement property was made, and if so, what rules were used to reach an overall judgment.
- If any adaptations to an existing tool to assess risk of bias in studies were made (such as omitting or modifying items), specify the adaptations.[2]
- If a new risk of bias tool was developed for use in the review, describe the content of the tool and make it publicly accessible.[2]
- Report how many reviewers assessed risk of bias in each study, whether multiple reviewers worked independently (such as assessments performed by one reviewer and checked by another), and any processes used to resolve disagreements between assessors.[2]
- Report any processes used to obtain or confirm relevant information from study investigators.[2]
- If automation tools/AI were used to assess risk of bias in studies, report how the automation tool was used (such as machine learning models to extract sentences from articles relevant to risk of bias), how the tool was trained, and details on the tool's performance and internal validation.[2]

Example of item #17

Example 1: "Two authors […] independently evaluated the measurement properties in each article against the COSMIN Risk of Bias checklist. […] Study quality was assessed separately for each measurement property using a four-point rating system (very good, adequate, doubtful or inadequate). The 'worst score counts' principle was used, where the overall rating for each measurement property is given by the lowest rating of any standard in the box [citation provided].[29]

Example 2: Methodological quality of the included studies was evaluated using the COSMIN risk of bias checklist [citation provided]. Following the COSMIN manual for systematic reviews of PROMs [patient-reported outcome measures] and the COSMIN methodology for evaluating content validity [references provided], all procedures were conducted by two reviewers […] independently. The COSMIN risk of bias checklist included 10 aspects: PROM development, content validity, structural validity, internal consistency, cross-cultural validity/measurement invariance, reliability, measurement error, criterion validity, hypotheses testing for construct validity, and responsiveness. The methodological quality of each aspect was assessed and rated on a 4-point scale: “very good” (V), “adequate” (A), “doubtful” (D), and “inadequate” (I). The ratings were determined based on “the worst score counts” principle, i.e., the lowest rating for any item was the rating for the study.[32]

Example 3: “Methodological quality assessment: The methodological quality of the included studies was assessed by two independent reviewers, using the COSMIN Risk of Bias (RoB) checklist [citation provided]. The studies’ methodological quality was assessed per measurement property separately. That is, per measurement property, only the boxes pertaining to that measurement property were used. Each box consists of four or more items, all of which were rated on a 4-point rating scale (i.e., “very good”, “adequate”, “doubtful”, or “inadequate”). The studies’ overall score per measurement property was equal to the lowest rated item of the respective box (i.e., "the worst score counts" principle). Discrepancies between reviewers were discussed and solved by consensus.”[28]

Example 4: “The tool recommended by the Agency for Healthcare Research and Quality (AHRQ) [citation provided] was adopted to assess the risk of bias of include studies. The following criteria were assessed: selection bias and confounding, performance bias, attrition bias, detection bias, reporting bias, and other bias [...]. Each item was judged as low risk of bias, high risk of bias or unclear on consensus between two reviewers. Disagreement was resolved by consulting a third reviewer.”[34]

Measurement properties

Item #18: Specify the methods used to rate the results of a measurement property for each individual study and for the summarized or pooled results, e.g., including how many reviewers rated each study and whether they worked independently.

Explanation: To interpret the results, users need to know what criteria for measurement properties have been used within each individual study and across studies (i.e., summarized or pooled results). Authors should specify the criteria used to rate the measurement properties' results within each individual study and across studies. If construct validity and responsiveness are evaluated in the review, authors should specify the (a priori) hypotheses used (e.g., about the expected direction and magnitude of correlations between the OMI of interest and comparison OMIs, as well as expected differences in scores between relevant groups) to rate the results of these measurement properties. Reporting details of how results were rated (such as by one or two authors, whether a logbook/rulebook was used) allows readers to assess the potential for errors in the ratings. If reviewers worked independently, it should be stated how discrepancies were resolved.

Essential elements

- Specify the criteria used to rate the results of each measurement property studied for each individual study and for the summarized or pooled results.
- If any adaptations to existing criteria for measurement properties' results were made, specify the adaptations.
- If construct validity and responsiveness were evaluated, specify the hypotheses used to rate the results of these measurement properties.
- If criterion validity was evaluated, provide a justification as to why the OMI can be considered a gold standard for the construct of interest.
- Report how many reviewers rated the results of each measurement property for each individual study and for the summarized or pooled results, whether multiple reviewers worked independently, and any processes used to resolve disagreements between assessors.

Example of item # 18

"[…] criteria for good measurement properties were applied to each result using the quality criteria [citation provided], resulting in a sufficient (+), insufficient (−), or indeterminate (?) rating (online supplemental appendix 3). A priori hypotheses were formulated to evaluate the results on construct validity and responsiveness. Figure 1 shows the predefined hypotheses for comparisons with other instruments. Hypotheses for comparisons between relevant subgroups or before and after intervention were: effect size (e.g., Cohen's D, standardized response mean) ≥0.20 for differences between relevant subgroups, score differences between relevant subgroups ≥10% (e.g., people with type 2 diabetes should score 10% worse than controls), or correlation ≥0.30 between relevant subgroups and score. Relevant subgroups were selected in consultation with an expert on type 2 diabetes. [...] evidence from multiple individual studies on the same PROM or subscale was summarized per measurement property and the summarized result was rated against the quality criteria for good measurement properties [citation provided]. […] Each step of the quality evaluation was done by two reviewers independently. Discrepancies were resolved by discussion and/or consultation of a third reviewer."[18]

The following are reproduced versions of Figure 1 and Appendix 3 in the review by Elsman et al., 2022.[18]

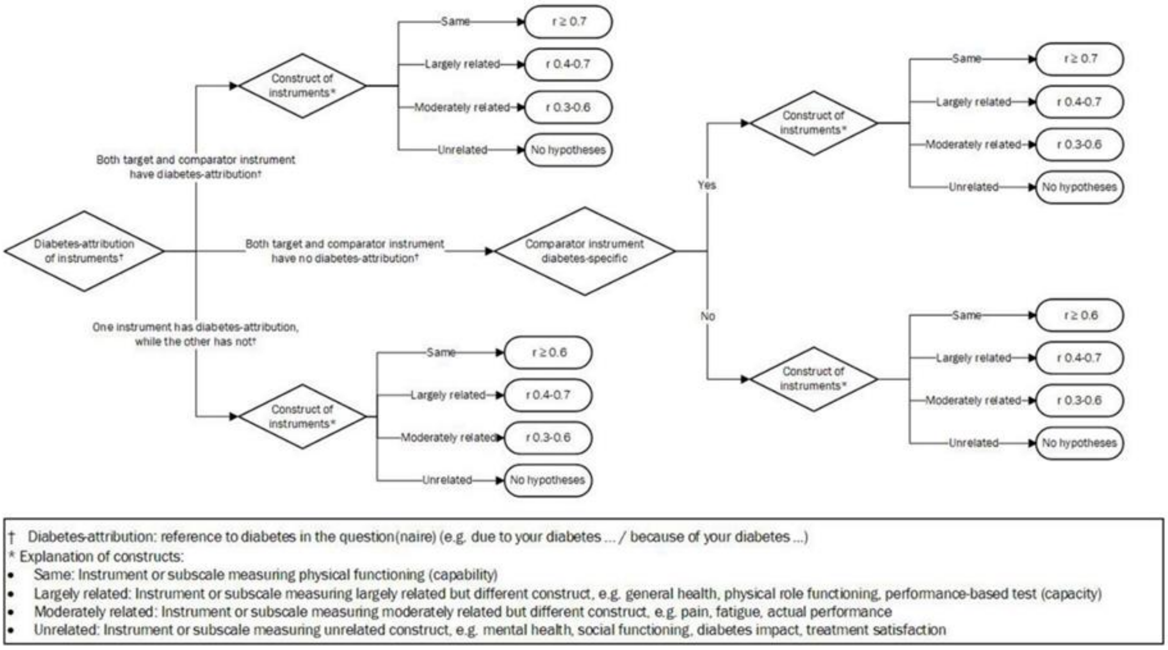

Figure 1 Decision tree for hypotheses regarding the comparisons of instruments.

Appendix 3. Criteria for good measurement properties

Measurement property	Rating	Criteria
Structural validity*	+	**CTT:**<br>EFA/PCA: factor loadings of each item on its factor is at least 0.30<br>*AND*<br>maximum 10% of the items load on more than one factor<br>*AND*<br>minimum explained variance is 50% and structure is in line with the theory about the construct to be measured *OR* results on scree plot or Kaiser criterion (Eigenvalues >1) are in line with the theory about the construct to be measured<br><br>CFA: CFI or TLI or comparable measure >0.95 *OR* RMSEA <0.06 *OR* SRMR <0.08<br><br>**IRT/Rasch:**<br>no violation of <u>unidimensionality:</u> CFI or TLI or comparable measure >0.95 *OR* RMSEA <0.06 *OR* SRMR <0.08<br>*AND*<br>no violation of <u>local independence:</u> residual correlations among the items after controlling for dominant factor <0.20 *OR* Q3's <0.37<br>*AND*<br>no violation of <u>monotonicity:</u> adequate looking graphs *OR* item scalability >0.30<br>*AND*<br>adequate <u>model fit:</u><br>IRT: χ^2 >0.01<br>Rasch: infit and outfit mean squares ≥0.5 and ≤1.5 *OR* Z-standardized values >-2 and <2
	?	**CTT:** not all information for '+' reported<br>**IRT/Rasch:** model fit not reported
	-	Criteria for '+' not met
Internal consistency	+	At least low evidence for sufficient structural validity *AND* Cronbach's alpha(s) ≥0.70 for each unidimensional scale or subscale
	?	Criteria for "at least low evidence for sufficient structural validity" not met
	-	At least low evidence for sufficient structural validity *AND* Cronbach's alpha(s) <0.70 for each unidimensional scale or subscale
Reliability	+	ICC or (weighted) kappa or Pearson/Spearman correlation ≥0.70
	?	ICC or (weighted) kappa or Pearson/Spearman correlation not reported
	-	ICC or (weighted) kappa or Pearson/Spearman correlation <0.70
Measurement error	+	SDC or LoA <MIC
	?	MIC not defined
	-	SDC or LoA > MIC
Hypotheses testing for construct validity	+	≥75% of the results is in accordance with predefined hypotheses
	?	No hypotheses defined (by the review team)
	-	≥75% of the results is not in accordance with predefined hypotheses
Cross-cultural validity\ measurement invariance	+	No important differences found between group factors (such as age, gender, language) in multiple group factor analysis *OR* no important DIF for group factors (McFadden's R^2 <0.02)
	?	No multiple group factor analysis *OR* DIF analysis performed
	-	Important differences between group factors *OR* DIF was found
Criterion validity	+	Correlation with gold standard ≥0.70 *OR* AUC ≥0.70
	?	Not all information for '+' reported
	-	Correlation with gold standard <0.70 *OR* AUC <0.70
Responsiveness	+	≥75% of the results is in accordance with predefined hypotheses *OR* AUC ≥0.70
	?	No hypotheses defined (by the review team)
	-	≥75% of the results is not in accordance with predefined hypotheses *OR* AUC <0.70

AUC = area under the curve, CFA = confirmatory factor analysis, CFI = comparative fit index, CTT = classical test theory, DIF = differential item functioning, EFA = exploratory factor analysis, ICC = intraclass correlation coefficient, IRT = item response theory, LoA = limits of agreement, MIC = minimal important change, PCA = principal component analyses, RMSEA: Root Mean Square Error of Approximation, SEM = Standard Error of Measurement, SDC = smallest detectable change, SRMR: Standardized Root Mean Residuals, TLI = Tucker-Lewis index

*Standard 1 in Box 3 in the COSMIN Risk of Bias checklist[32] was rated very good if CFA was performed, adequate if EFA was performed, doubtful if PCA was performed and inadequate if none of the previous was performed.

Example 2: "For the diagnosis of uncomplicated UTIs [urinary tract infections], urine analysis is considered the gold standard, with appropriate clinical examinations and typical symptom assessment. However, such a diagnosis is not suitable to evaluate impact and bothersomeness of UTI or any PROs [patient-reported outcomes] in UTI, but the clinical diagnosis lends itself for the evaluation of known-groups validity by comparing PROM [patient-reported outcome measure] scores of women with and without diagnosed UTI. For interpreting the results of studies on hypotheses testing for construct validity, and on studies using a construct approach for the evaluation of responsiveness, a priori hypotheses were formulated for each PROM. [...] With respect to responsiveness, we expected improvement of the scores in all domains after antibiotic treatment. The evaluation of the quality of hypotheses testing for construct validity and responsiveness using a construct approach was performed according to the generic hypotheses as outlined in the COSMIN manual: (1) Correlations with (changes in) instruments measuring similar constructs should be ≥ 0.50, (2) Correlations with (changes in) instruments measuring related, but dissimilar constructs should be lower, i.e., 0.30–0.50, (3) Correlations with (changes in) instruments measuring unrelated constructs should be < 0.30, (4) Correlations defined under 1, 2, and 3 should differ by a minimum of 0.10; (5) Meaningful changes between relevant (sub)groups; and (4) AUC should be ≥ 0.70 for responsiveness. [...] the result of each single study on a measurement property was rated against the criteria for good measurement properties. Measurement properties were rated as either sufficient (+), insufficient (–), or indeterminate (?). [...] The summarized results were then rated against the criteria for good measurement properties (Table 3)."[27]

Table 3 in the review by Piontek et al.[27] shows a similar table as Appendix 3 in the review by Elsman et al.,[18] shown in *Example 1.*

Example 3: "Results obtained from single studies on measurement properties were rated against COSMIN's updated criteria for good measurement properties. Each result was rated as either sufficient (+), insufficient (−), or indeterminate (?). For studies reporting on content validity, the quality of the results were rated using the criteria for relevance (5), comprehensiveness (1), and comprehensibility (4). Regarding hypothesis testing for construct validity and responsiveness, COSMIN recommends setting a priori hypotheses prior to review commencement. Following De Vet et al., for both measurement properties, correlations were expected to be: ≥ 0.50 with instruments measuring similar constructs; < 0.50 and ≥ 0.30 with instruments measuring related but dissimilar constructs; and < 0.30 with instruments measuring unrelated constructs. No hypotheses were formulated for expected differences between groups (e.g., age, gender) for discriminant and known-groups validity. [...] an overall rating of study results per measurement property per tool was summarized as sufficient (+), insufficient (−), indeterminate (?), or inconsistent (±). Specifically, an overall rating was determined through combining the scoring of each single study; if ≥75% of the studies displayed the same scoring, that scoring became the overall rating (+ or −), whereas if < 75% of studies displayed the same scoring, the overall rating became inconsistent (±)."[24]

Synthesis methods

Item #19a: *Describe the processes used to decide which studies were eligible for each synthesis.*

Explanation: Before the measurement properties of relevant OMIs can be synthesized (item #13d), decisions must be made about which individual studies are eligible to include for each synthesis.[2] Often, results of multiple studies on the same measurement property of the same OMI are synthesized.[7] Inconsistency in the results of studies or differences in the populations in which these results were found can influence the decision on which studies to synthesize. These decisions will likely involve some degree of subjective judgement that could alter the results of a synthesis.[2] Therefore, the selection processes and any supporting information should be reported for transparency of the decision made.[2]

Essential elements

- Describe the processes used to decide which studies were eligible for each synthesis.[2]

Example of item #19a

Example 1: "The summary of the overall evidence of measurement properties of the PROMs was determined by the number of studies, the methodological quality of the studies, and consistency of the findings."[95]

Example 2: "Multiple articles were combined if they concerned the same physical capacity task and included samples with comparable characteristics."[96]

Example 3: "[…] evidence from multiple individual studies on the same PROM or subscale was summarized per measurement property […]."[18]

Item #19b: *Describe any methods used to synthesize results.*

Explanation: Various methods are available to synthesize results.[2] In systematic reviews of OMIs, the most common method is to qualitatively summarize measurement properties' results (i.e., for content validity, structural validity, and cross-cultural validity\measurement invariance), provide the number of confirmed and unconfirmed hypotheses (i.e., for construct validity, and responsiveness), or give a range of the measurement properties' results across individual studies (i.e., for criterion validity, internal consistency, reliability, and measurement error).[7] For some measurement properties (i.e., internal consistency, reliability, measurement error, construct validity, and responsiveness), it is possible to pool the results or perform a meta-analysis, although this is not common for systematic reviews of OMIs, as the point estimates of these results are commonly not used as such. Regardless of the chosen synthesis method(s), authors should provide sufficient detail such that readers are able to assess the appropriateness of the selected methods and could reproduce the reported results (if they had access to the data).[2]

Essential elements

- Describe and justify the summary approach or synthesis method used.
- If different approaches are used for different measurement properties, describe which approach was used for each measurement property.
- If statistical synthesis methods were used, reference the software, packages, and version numbers used to implement synthesis methods (such as metafor (version 2.1-0) in R).[2,97]
- If meta-analysis was done, specify:[2]
  - the meta-analysis model (fixed-effect, fixed-effects, or random-effects) and provide rationale for the selected model.
  - the method used (such as Mantel-Haenszel, inverse-variance).[98]
  - any methods used to identify or quantify statistical heterogeneity (such as visual inspection of results, a formal statistical test for heterogeneity,[98] heterogeneity variance (τ2), inconsistency (such as I^2),[99] and prediction intervals).[100]
- If a planned synthesis was not considered possible or appropriate, report this and the reason for that decision.[2]

Example of item #19b

Example 1: "Individual ratings for each measurement property were qualitatively synthesized using a priori rules based on those recommended by COSMIN […]. Based on these rules, each instrument could receive an overall (synthesized) rating of sufficient (+), insufficient (−), or inconsistent (±) for each measurement property (with content validity additionally split into relevance, comprehensibility, and comprehensiveness)."[41]

Example 2: "[…] either a meta-analysis or narrative synthesis was conducted, based on the heterogeneity of the included studies. For a meta-analysis to be indicated, an adequate number of studies that contained similar study demographics, design and low/moderate heterogeneity were needed to be included. The I2 statistical analysis was used to evaluate the variation between studies that was due to heterogeneity rather than chance. Heterogeneity was considered 'substantial' if the I2 scores were > 50%. The meta-analysis was performed in R (version 1.4.1106). Due to the expected variability between the studies, the standard generic inverse variance random effects model was

used. […] For the outcomes where there was a lack of homogeneity, a narrative synthesis was conducted in line with the narrative synthesis in systematic reviews recommendation."[46]

Example 3: "[…] a qualitative synthesis of the evidence per measurement property, per PROM [patient-reported outcome measure] was constructed to come to an overall conclusion of PROM quality. If consistent (i.e., ≥ 75% of the results are either rated 'sufficient' or 'insufficient'), the results of the individual studies on measurement properties were qualitatively summarized and again rated against the criteria for good measurement properties. If inconsistent, an explanation for this inconsistency was sought. When the inconsistency remained unexplained, the overall result was rated as 'inconsistent' (±). An 'indeterminate' (?) rating was given when the individual results were all rated as 'indeterminate'."[36]

Item #19c: *If applicable, describe any methods used to explore possible causes of inconsistency among study results (e.g., subgroup analysis).*

Explanation: If authors used methods to explore possible causes of variation in results across studies (that is, inconsistency) they should provide details about which causes were explored to explain inconsistency, and how they dealt with the inconsistency, so that readers are able to assess the appropriateness of the selected methods and could reproduce the reported results (if they had access to the data).[2] Possible causes of inconsistency might for example be participant or OMI characteristics, risk of bias in the included studies, study methods, or study recentness.[7] Subgroup analyses can be conducted if variation in results across studies can be explained by one of these causes. This involves splitting studies into subgroups and comparing the results in the subgroups.[2] Authors might use subgroup analyses to explore whether the measurement properties' results varied with for example different participant characteristics (such as acute versus chronic conditions) or study quality (such as very good/adequate studies versus inadequate studies).[2,7]

Essential elements

- If methods were used to explore possible causes of inconsistency, specify which causes were explored.
- If methods were followed to deal with inconsistency, specify the methods used (such as subgroup analysis, ignoring certain results).

Example of item #19c

Example 1: "When individual studies showed inconsistent results, explanations for inconsistency in terms of differences in populations or study quality were explored. When inconsistency could be explained, results were summarized and rated per subset of studies. When inconsistency could not be explained, the overall rating was inconsistent (±), without summarizing the results or based on the majority of consistent results (+, −, or ?). If studies with a + or − rating were available, studies with a ? were ignored and not included when summarizing the results."[18]

Example 2: "When the number of studies is sufficient (n ≥ 3), subgroup analyses were conducted to explore the potential sources of heterogeneity. Subgroup were defined a priori and included running speed, IMUs' [inertial measurement units] position and running surface. The running speed was set to two levels: low (speed ≤ 15 km/h) and fast (speed > 15 km/h), and the running surface was divided into treadmill and ground."[101]

Example 3: "If the ratings of each study were inconsistent, we explored possible explanations (e.g., different languages). If the explanation was reasonable, we provided ratings by subgroup. If the explanation was unreasonable, the overall rating of the measurement property was rated as inconsistent (±). If there was no information to support the rating, the overall rating was rated as uncertain (?)."[31]

Item #19d: *If applicable, describe any sensitivity analyses conducted to assess robustness of the synthesized results.*

Explanation: Sensitivity analyses are undertaken to examine the robustness of findings to decisions made during the review process.[2] This involves repeating an analysis but using different decisions from those originally made and comparing the findings.[2,98] For example, sensitivity analyses might have been done to examine the impact on the results if studies were included that were just outside the population of interest, or if studies with high risk of bias were ignored.[2] If authors performed sensitivity analyses to assess the robustness of the synthesized results to decisions made during the review process, they should provide sufficient details so that readers are able to assess the appropriateness of the analyses and could reproduce the reported results (if they had access to the data).[2] Ideally, sensitivity analyses should be pre-specified, but unexpected issues may emerge during the review process that necessitate their use.

Essential elements

- If sensitivity analyses were performed, provide details of each analysis (such as removal of studies at high risk of bias, use of an alternative synthesis method).[2]

Additional elements

- Consider identifying any sensitivity analyses that were not pre-specified, if any.[2]

Example of item #19d

Example 1: "Sensitivity analyses were performed for methodological quality and test procedure by restricting the meta-analyses to studies with an RoB [risk of bias] rating of "adequate" or "very good" and specific starting knee angles, respectively. Statistical significance was set at $P < 0.05$."[102]

Example 2: "Sensitivity analyses were performed by deleting one study at a time to evaluate the stability of the results."[101]

Certainty assessment

Item #20: Describe any methods used to assess certainty (or confidence) in the body of evidence.

Explanation: Authors typically use some criteria to decide how certain (or confident) they are in the body of evidence for each measurement property of an OMI in relation to the purpose of measurement and context of use. Common factors considered include study design limitations (risk of bias), consistency of findings across studies, sample size (i.e., imprecision), and how directly the studies address the research question.[2] Tools and frameworks can be used to provide a systematic, explicit approach to assessing these factors and provide a common approach and terminology for communicating certainty.[2,11,103] For example, the modified GRADE approach allows authors to grade the quality of the evidence, taking risk of bias, inconsistency of results, imprecision, and indirectness into consideration.[7] These factors result in an overall judgment of whether the evidence supporting a result is of high, moderate, low, or very low certainty. This is done for the synthesized result of each measurement property of an OMI. Reporting the factors considered and the criteria used to assess each factor enables readers to determine which factors fed into reviewers' assessment of certainty.[2] Reporting the process by which assessments were conducted enables readers to assess the potential for errors and facilitates replication.[2]

Essential elements

- Specify the tool or system (and version) used to assess certainty in the body of evidence.[2]
- Report the factors considered (such as risk of bias, inconsistency of results, imprecision, and indirectness) and the criteria used to assess each factor when assessing certainty in the body of evidence.[2]
- Describe the decision rules used to arrive at an overall judgment of the level of certainty (such as high, moderate, low, very low), together with the intended interpretation (or definition) of each level of certainty.[2,103]
- If any adaptations to an existing tool or system to assess certainty were made, specify the rationale and adaptations in sufficient detail that the approach is replicable.[2]
- Report how many reviewers assessed the certainty of evidence, whether multiple reviewers worked independently, and any processes used to resolve disagreements between assessors.[2]

Where a published system is adhered to, it may be sufficient to briefly describe the factors considered and the decision rules for reaching an overall judgment and reference the source guidance for full details of assessment criteria.[2]

Example of item #20

Example 1: "[…] the quality of the evidence was graded using a modified Grading of Recommendations Assessment, Development and Evaluation (GRADE) approach resulting in 'high', 'moderate', 'low', or 'very low' quality [citation provided]. Quality of the evidence was not graded for studies for which the overall rating was indeterminate (?). For all other situations, starting with high-quality evidence, quality of evidence was down-graded (online supplemental appendix 4). For internal consistency, the quality of evidence started at the level of structural validity. Each step of the quality evaluation was done by two reviewers independently. Discrepancies were resolved by discussion and/or consultation of a third reviewer."[18]

The following is a reproduced version of Appendix 4 in the review by Elsman et al., 2022.[18]

Appendix 4. Approach for grading the quality of the evidence

Grade factor	Downgrading	Definition
Risk of bias	0	Multiple studies of at least adequate quality *OR* one study of very good quality
	-1	Only one study of adequate quality *OR* multiple studies of doubtful quality
	-2	Only one study of doubtful quality *OR* multiple studies of inadequate quality
	-3	Only one study of inadequate quality
Imprecision (not for content validity, structural validity, and cross-cultural validity\ measurement invariance)	0	Total sample size of all studies >100
	-1	Total sample size of all studies 50-100
	-2	Total sample size of all studies <50
Inconsistency	0	Results are consistent *OR* results are summarized and rated per subset of studies, and subsequently graded
	-1	Overall rating based on the majority of consistent results
Indirectness	0	Does not occur; definitions for construct and/or target population have been stated in the inclusion criteria

0: high, -1: moderate, -2: low, -3: very low; Per protocol of the COSMIN guideline for systematic reviews: the quality of evidence for internal consistency cannot be higher than the quality of evidence for structural validity[23]

Example 2: "The quality of evidence was graded using the Grading of Recommendations Assessment, Development, and Evaluation (GRADE) approach considering the methodological quality of studies, total sample size, and consistency of results [citation provided]. In case of concerns regarding the trustworthiness of a result, the quality of evidence of the summarized results was downgraded per measurement property per PROM. Downgrading was possible due to risk of bias, inconsistency, imprecision, and/or indirectness. The quality of evidence was rated as either high, moderate, low, or very low. We did not grade the quality of evidence if an overall rating was indeterminate or inconsistent."[27]

Example 3: "In accordance with COSMIN guidelines, a modified Grading of Recommendations Assessment, Development, and Evaluation (GRADE) approach was used for grading the evidence [citation provided]. The summarized results were graded as 'high', 'moderate', 'low' or 'very low', based on three factors: risk of bias (based on methodological quality), inconsistency and imprecision (i.e., sample size). The fourth factor 'indirectness' was not taken into consideration in evaluating evidence quality, this review only included studies with a predefined and fixed patient population. If the quality of the summarized result was rated 'inconsistent' or 'indeterminate', the quality of the evidence could not be graded. The above-mentioned subsequent steps of the COSMIN evaluation were performed by two reviewers independently. If consensus could not be reached during any of the evaluation procedures, an additional reviewer was consulted."[36]

Formulating recommendations

Item #21: If appropriate, describe any methods used to formulate recommendations regarding the suitability of OMIs for a particular use.

Explanation: Systematic reviews of OMIs are conducted for a variety of reasons (e.g., to select the most suitable available OMI for a particular use, or to identify gaps in knowledge in the measurement properties of available OMIs). If the rationale is to select the most suitable OMI for a particular use, recommendations can be made regarding the suitability of OMIs. Although systematic reviews might include evidence that could be important in more than one context, decisions about what tools are most useful might depend on time, place, and population characteristics. If recommendations regarding the suitability of OMIs for particular uses are formulated, authors should provide details about the methods and processes used to make these recommendations to enable readers to assess the aspects that informed the recommendations. This also includes specifying how each of the measurement properties considered in the review were taken into account while formulating recommendations. Recommendations can be based upon published guidelines (e.g., [7,16]). In some cases, making recommendations might not be appropriate or allowable, for example if making recommendations is not permitted by the funder of the review or is not in line with the rationale for the systematic review.

Essential elements

- If methods were used to formulate recommendations, specify what formed the basis of recommendations.
- Specify which measurement properties were used in formulating recommendations.

Example of item #21

Example 1: "To formulate recommendations, we considered the results on the measurement properties in order of importance. According to COSMIN, PROMs [patient-reported outcome measures] that have any level of sufficient content validity, which is the most important measurement property, and at least low-quality evidence for sufficient internal consistency (and as such also at least low-quality evidence for sufficient structural validity) can be recommended for use, except when there is high-quality evidence for any insufficient measurement property [citation provided]. We subsequently took results on reliability into account when formulating recommendations, and considered construct validity and responsiveness as least important. Importantly, we also took into account the limitations of the PROMs arising from the recommendations."[18]

Example 2: "Evidence on each metric property from studies using good or amber methods was extracted and summarized in Summary of Measurement Properties (SOMP) tables. Each measurement property was given a final rating based on the gathered evidence according to OMERACT [Outcome Measures in Rheumatology] guidance. A green rating indicates consistently good performance from multiple studies identified as having good methods; amber indicates a noncritical limitation in the evidence, which merits a research plan. Finally, an overall rating across all the measurement properties for each instrument was proposed by the working group, evaluated by the TAG [technical advisory group] and finally brought to a broader group of the OMERACT community for final approval of our proposed level of endorsement."[35]

Results

Study selection

Item #22a: Describe the results of the search and selection process, from the number of records identified in the search to the number of study reports included in the review, ideally using a flow diagram. If applicable, also report the final number of OMIs included and the number of study reports relevant to each OMI.

Explanation: Review authors should report, ideally with a flow diagram, the results of the search and selection process so that readers can understand the flow of retrieved records through to inclusion in the review.[2] Such information is useful for future systematic review teams seeking to estimate resource requirements and for information specialists in evaluating their searches.[2,104,105] Specifying the number of records yielded per database and from additional sources will make it easier for others to assess whether they have successfully replicated a search.[2] In addition to the reports included in the systematic review, authors should also report the number of OMIs included in the review and indicate how many reports were found for each OMI. The PRISMA-COSMIN for OMIs 2024 flow diagram, presented below, provides a template of the flow of records through the review separated by source in which the number of OMIs can be reported as well, although other layouts may be preferable depending on the information sources consulted.[2,91] For example, review authors may opt to complete a separate flow diagram for each OMI included, or specify the number of studies or measurement properties for each OMI.

Essential elements

- Report, ideally using a flow diagram, the number of: records identified from each source; records excluded before screening (for example, because they were duplicates or deemed ineligible by machine classifiers); records screened; records excluded after screening titles or titles and abstracts; reports retrieved for detailed evaluation; potentially eligible reports that were not retrievable; retrieved reports that did not meet inclusion criteria and the primary reasons for exclusion (such as ineligible outcome domain, ineligible population, or ineligible (type of) OMI);[2] and the number of reports and OMIs included in the review, indicating how many reports were found for each OMI.
- If the review is an update of a previous review, report results of the search and selection process for the current review and specify the number of reports and OMIs included in the previous review. An additional box could be added to the flow diagram indicating the number of studies included in the previous review.[2]
- If applicable, indicate in the flow diagram how many records were excluded by a human and how many by automation tools/AI.[2]

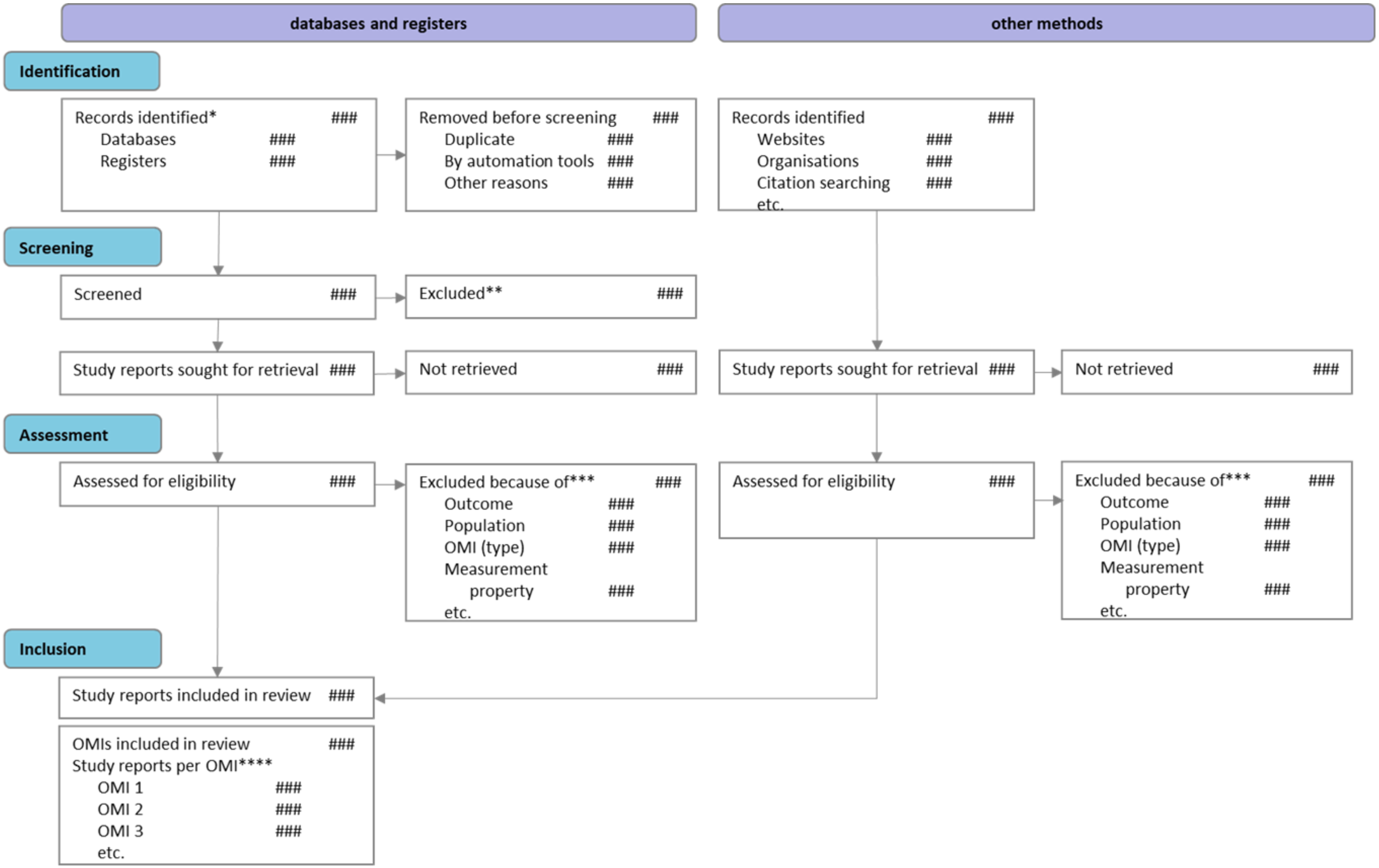

*Consider, if feasible to do so, reporting the number of records identified from each database or register searched (rather than the total number across all databases/registers).
**If automation tools were used, indicate how many records were excluded by a human and how many were excluded by automation tools.
***Change or add reasons of exclusion as applicable.
****Replace 'OMI 1' etc. by the OMI name or acronym.

Template for the PRISMA-COSMIN for OMIs 2024 flow diagram. The boxes below 'other methods' should only be completed if methods other than databases and registers were searched.

Example of item #22a

Example 1: "The database search and reference check resulted in 12771 unique abstracts, of which 341 were assessed full text for eligibility. Ultimately, 21 articles were included in this review, describing 12 versions of 7 unique PROMs or subscales measuring physical functioning."[18] A flow diagram is available at http://dx.doi.org/10.1136/bmjdrc-2021-002729.

Example 2: "The database searches found 10,037 publications after removing duplicates. Based on the title and abstract, 224 appeared to meet the inclusion criteria. After assessing the full text, 86 publications were included. Four additional publications were identified by checking reference lists and using citation tracking resources. In total, we included 90 publications and 62 questionnaire measurement instruments."[40] A flow diagram is available at https://doi.org/10.1016/j.jpsychores.2023.111161.

Item #22b: Cite study reports that might appear to meet the inclusion criteria, but which were excluded, and explain why they were excluded.

Explanation: Identifying the excluded records allows readers to make an assessment of the validity and applicability of the systematic review.[2,69] At a minimum, a list of reports containing studies that might appear to meet the inclusion criteria, but which were excluded, with citation and a reason for exclusion, should be reported.[2] This would include studies meeting most inclusion criteria (such as those with appropriate outcome domain, (type of) OMI and measurement property but an ineligible population). Often this concerns the reports retrieved for detailed evaluation (i.e., for full-text assessment). It is also useful to list reports that were potentially relevant but for which the full text or data essential to inform eligibility were not accessible,[2] or to list reports that were not available in the required language. This information can be provided as a list/table in the report or in the supplementary material.[2] Potentially contentious exclusions should be clearly stated in the report.[2]

Essential elements

- List reports that might appear to meet the inclusion criteria, but which were excluded, and explain why they were excluded in the report or in an online supplement.[2]

Example of item #22b

Example 1: The excluded full texts and the reasons for exclusion according to the inclusion criteria or the critical appraisal are listed in Appendix II."[52]

The following is an abridged version of Appendix II in the review by Kipfer et al., 2020.[52]

Reference	Reason for exclusion
Reynolds T, Thornicroft G, Abas M, Woods B, Hoe J, Leese M, *et al.* Camberwell Assessment of Need for the Elderly (CANE). Development, validity and reliability. Br J Psychiatry 2000;176:444–452.	Criteria 1
Nicolaou PL, Egan SJ, Gasson N, Kane RT. Identifying needs, burden, and distress of carers of people with frontotemporal dementia compared to Alzheimer's disease. Dementia 2010;9(2):215–235.	Criteria 1,4
van der Roest HG, Meiland FJM, Comijs HC, Derksen E, Jansen APD, van Hout HPJ, *et al.* What do community-dwelling people with dementia need? A survey of those who are known to care and welfare services. Int Psychogeriatr 2009;21(5):949-65.	Criteria 1,4
Orrell M, Hancock GA, Liyanage KCG, Woods B, Challis D, Hoe J. The needs of people with dementia in care homes: the perspectives of users, staff and family caregivers. Int Psychogeriatr 2008;20(5):941-51.	Criteria 1,2,4

Inclusion criteria recommended by the COSMIN guidelines for systematic reviews of measurement properties:[39] 1) the instrument should aim to measure the construct of interest (types of intervention(s)/phenomena of interest), 2) the study sample should concern the target population of interest (types of participants), 3) the study should concern the type of measurement instrument of interest (self-reported or professionally interviewed), 4) the aim of the study should be the development of a measurement instrument or the evaluation of one or more of its measurement properties (types of studies).

Example 2: "Excluded studies and the reasons for their exclusion are provided in Appendix S3."[106]

The following is an abridged version of Appendix S3 in the review by Baamer et al., 2022.[106]

Excluded papers:

1. Arnstein P, Gentile D, Wilson M. validating the functional pain scale for hospitalized adults. *Pain Manag Nurs*. 2019; **20:** 418-24.

Explanation: Paper validating functional scale for hospitalized chronic pain patient but did not report separte result for surgical patients.

Reason for exclusion: No separate results for postoperative pain assessment.

2. Barber MD, Janz N, Kenton K, et al. Validation of the surgical pain scales in women undergoing pelvic reconstructive surgery. *Female Pelvic Med Reconstr Surg*. 2012; **18:** 198-204.

Explanation: Surgical pain scale looked at long term functional outcome following surgery.

Reason for exclusion: Patients not assessed as inpatients/irrelevant outcome.

3. McCarthy Jr M, Chang CH, Pickard AS, et al. Visual analog scales for assessing surgical pain. *Jl Amn Coll Surg*. 2005; **201:** 245-52.

Reason for exclusion: Patients not assessed as inpatients or irrelevant outcome.

4. Blumstein HA, Moore D. Visual analog pain scores do not define desire for analgesia in patients with acute pain. *Acad Emerg Med.* 2003; **10:** 211-4.

Explanation: VAS to detect desire of analgesia in acute emergency pain.

Reason for exclusion: Not surgical population.

Example 3: ""Excluded full text articles are listed in S4 Table."[107]

The following is an abridged version of S4 Table in the review by Mihaljevic et al., 2022.[107]

Ref.	Abbreviation	Reason for exclusion
Development and evaluation of a questionnaire to assess patient satisfaction with chemotherapy nursing care. Eur J Oncol Nurs 1999;3:126–140	N/A	No multidimensional PREM
Evaluation properties of the French version of the OUT-PATSAT35 satisfaction with care questionnaire according to classical and item response theory analyses. Qual Life Res 2014	OUT-PATSAT35	PREM for outpatient sector
The cancer outpatient satisfaction with care questionnaire for chemotherapy, OUT-PATSAT35 CT: a validation study for Spanish patients. Support Care Cancer 2012;20:3269–3278.	OUT-PATSAT35-CT	PREM for outpatient sector
The EORTC cancer outpatient satisfaction with care questionnaire in ambulatory radiotherapy: EORTC OUT-PATSAT35 RT. Validation study for Spanish patients. Psycho-Oncology 2010;19(6):657–664	OUT-PATSAT35-RT	PREM for outpatient sector
Multisite validation study of questionnaire assessing out-patient satisfaction with care questionnaire in ambulatory chemotherapy or radiotherapy treatment. Bull Cancer 2006;93:315–327	N/A	PREM for outpatient sector
Development and evaluation of a questionnaire to assess patient satisfaction with chemotherapy nursing care. Eur J Oncol Nurs 1999;3:126–140	WCSQ	No generic, surgery- or cancer care-specific PREM

Example 4: "[...] (see Appendix III for excluded studies with exclusion reasons)."[30]

The following is an abridged version of Appendix III in the review by Halvorsen et al., 2023.[30]

Appendix III Supplementary material: Excluded studies with exclusion reasons

Abdelghani, E. A., Apollonsky, N., Bernstein, B., & Tarazi, R. (2017). Steady-state cognitive function and pain severity in youth with sickle cell disease. *Blood. Conference: 59th Annual Meeting of the American Society of Hematology, ASH, 130*(Supplement 1). Exclusion reason: Wrong patient population

Abozeid, M., Hamouda, M., Bahry, H., Elmadny, A., Alakbawy, A., & Ismail, A. (2011). Psychiatric morbidity among a sample of orphanage children in Cairo. *European Child and Adolescent Psychiatry, 1)*, S166-S167. https://doi.org/10.1007/s00787-011-0181-5. Exclusion reason: Conference Abstract

Accordino, R. E., Kidd, C., Politte, L. C., Henry, C. A., & McDougle, C. J. (2016). Psychopharmacological interventions in autism spectrum disorder. *Expert Opinion on Pharmacotherapy, 17*(7), 937-952. https://doi.org/10.1517/14656566.2016.1154536. Exclusion reason: Review

OMI characteristics

Item #23a: Present characteristics of each included OMI, with appropriate references.

Explanation: Providing characteristics of the OMIs included in the review allows readers to understand what the included OMIs look like and understand the applicability of the review. As some OMIs may be available in different formats or versions, this information will also allow readers to understand the differences between formats or versions. Characteristics of the OMI(s) include the outcome domain of interest, the target population for which the OMI was developed, the mode of administration, the recall period, the (sub)scales and number of items, the response options, the ranges of scores or scoring method, the original language in which the OMI was developed, and any available translations. Additional characteristics can be reported as applicable.

Essential elements

- Present characteristics of each OMI in a table (considering a format that will facilitate comparison of characteristics across OMIs)
- Provide appropriate references for each OMI, for example the first report on the OMI in the literature (e.g., the development paper), which may be different from the reports selected for inclusion in the review.

Example of item # 23a

Example 1: In a review examining the measurement properties of situational awareness instruments in healthcare providers,[108] the authors included a table combining the characteristics of included OMIs with characteristics of included studies (item #24).

The following is an abridged version of Table 1 in the review by Ghaderi et al., 2023.[108]

Table 1 Characteristics of the instruments used to measure SA in HCPs and description of the included studies

Instrument name	Type of measure	Number of subscales	Total items	Response Options	Reference country	Study participants	Setting (clinical vs. simulation)	Measurement properties
Anesthetists' Non-Technical Skills System (ANTS)	Observational checklist	4	15	4-point rating scale	Fletcher et al., 2003 [28] UK	50 anesthetists	Simulation (simulated anesthetic scenarios)	Content validity<br>Internal consistency<br>Inter-rater reliability
Anesthetists' Non-Technical Skills System (ANTS)	Observational checklist	4	15	4-point rating scale	Graham et al., 2010 [29] Australia	26 anesthetists	Clinical (videos of real-time and routine anesthesia)	Internal consistency<br>Inter-rater reliability
Anaesthetic Non-technical Skills for Anesthetic Practitioners System (ANTS-AP)	Observational checklist	3	9	4-point rating scale	Rutherford et al.,2015 [30] UK	48 anesthetic practitioners	Simulation (Simulated anesthetic scenarios in OR	Content validity<br>Internal consistency<br>Reliability<br>Inter-rater reliability
Trauma Non-Technical Skills (T-NOTECHS) tool	Observational checklist	5	5	5-point scale	van Maarseveen et al., 2020 [31] Netherland	18 recorded videos of resuscitations team 3 assessors	Clinical (trauma center)	Reliability<br>Inter-rater reliability

Example 2: In a review examining the measurement properties of diabetes-specific PROMs measuring physical functioning,[18] the authors included a table presenting for each included OMI the first citation, the construct, target population, mode of administration, recall period, subscales and number of items, language of the OMI and available translation.

The following is an abridged version of Table 1 in the review by Elsman et al., 2022.[18]

Table 1 Characteristics of included diabetes-specific PROMs measuring physical functioning in people with type 2 diabetes (n=11)

PROM—subscale	Construct(s)	Target population	Mode of administration	Recall period	(Sub)scale(s) and number of items*	Original language	Available translations
Diabetic Foot Ulcer Scale (DFS)—Daily activities[42]	PROM: Impact of diabetic foot ulcers on quality of life Included subscale: NR	People with diabetes and foot ulcers	Self-report	NR	11 subscales, 58 items: **Daily activities—six items**	English[42]	Dutch, Danish, Italian, French†
Diabetic Foot Ulcer Scale—Short Form (DFS-SF)—Dependence/daily life[34]	PROM: Impact of diabetic foot ulcers on quality of life Included subscale: Issues related to dependence on others and changes in daily activities	People with diabetes and foot ulcers	Self-report‡	Depending on assessment point, varying from 4 to 20 weeks	Six subscales, 29 items: **Dependence/daily life—five items**	English[34]	Polish,[45] Chinese,[38] Greek,[60] Spanish,[61] Dutch, Danish, Italian, French†
Patient-reported outcomes instrument for Thai patients with type two diabetes (PRO-DM-Thai)—Physical function[35]	PROM: Evaluate outcomes of diabetic care in terms of health and the process of care Included subscale: Relating to physical ability and measuring physical functioning, eg, mobility, dexterity, range of movement, physical activity, activities of daily living	Thai people with type 2 diabetes	Self-report/ interview based	NR	Seven subscales, 44 items: **Physical function—five items**	Thai[35]	
Impact of Weight on Activities of Daily Living Questionnaire (IWADL/ APPADL)—(Physical) activities of daily living[38]	PROM (=subscale): Ability to perform daily physical activities	People with type 2 diabetes with moderate obesity (BMI: 30–40)	Self-report	Current	One subscale, seven items: **Physical activities of daily living—seven items**	English[38 46]	

Example 3: In a review examining the concurrent validity and test–retest reliability of inertial measurement units for measuring gait spatiotemporal and lower-extremity kinematics outcomes during running in healthy adults,[101] the authors included a table combining the characteristics of included OMIs with characteristics of included studies (item #24).

The following is an abridged version of Table 3 in the review by Zeng et al., 2022.[101]

Table 3 Study characteristics

Author(s), Year [Reference No.]	Participant (size, age, height, weight, population)	IMUs					Reference system	Running speed/running distance	Research field	Parameters
		Name (manufacturer)	Composition	Number	Placement	Sample frequency				
Ammann et al., 2016 [32]	12 subjects (5 F, 7 M; age: 25.3 ± 3.2 years; height: 174.4 ± 7.9 cm; weight: 64.8 ± 10.2 kg) High-level running athletes	PARTwear (PW, HuCE-microLab, University of Applied Sciences, Biel, Switzerland)	3D accelerometer (± 16 g); 3D gyroscope; 3D magnetometer	2	Lace of the shoe	1000 Hz	OMC (Camera Marathon Ultra CL600, Videal AG, Niederonz, Switzerland)	Maximal sprinting speed (8.0 ± 0.5 m/s); intense training speed (6.2 ± 0.7 m/s); normal training speed (4.3 ± 0.7 m/s); all speeds (6.2 ± 1.6 m/s) (40 m)	Indoor track	Stance time
Bergamini et al., 2012 [33]	Group A: 6 amateur athletes (2F, 4 M; height: 172 ± 12 cm; weight: 63.50 ± 10.84 kg) Group E: 5 elite athletes (2F, 3 M; height: 177 ± 76 cm; weight: 65.00 ± 8.25 kg) Total: 11 participants (4F, 7 M; height: 174 ± 10 cm; weight: 64.18 ± 9.78 kg)	IMU (FreeSense, Sensorize, Italy)	3D accelerometer (± 6 g); 3D gyroscope (± 500°/s)	1	Lower back trunk (L1 level)	200 HZ	OMC (Casio Exilim EX-F1, Japan); 9 force platforms (Z20740AA, Kistler, Switzerland)	Three sprint runs of 60 m	Indoor track; outdoor training track	Stance time, stride time

Item #23b: If applicable, present interpretability aspects for each included OMI.

Explanation: Reporting information on interpretability of the included OMIs facilitates authors' conclusions regarding the suitability of OMIs and informs readers in the selection of the most suitable OMI for a specific purpose. Reporting information on interpretability is particularly relevant if the rationale for the review is to select the most suitable OMI for a particular use (e.g., for use as a primary outcome in research, for use in clinical practice, or for inclusion in a core outcome set). Information on interpretability helps inform the qualitative meaning (i.e., the clinical or commonly understood meaning) of an OMI's quantitative score.[12] Interpretability aspects might include the distribution of scores in the population, percentage of missing items and/or scores, floor and ceiling effects, scores and change scores for relevant (sub)groups (e.g., reference/norm scores), minimal important change or difference, and information on response shift. Authors could also report on the confidence they have in the meaning derived from the interpretation of OMI scores for an intended measurement purpose in an intended context of use. Presenting interpretability aspects of each OMI in a table can facilitate comparison of characteristics across OMIs. This table can be included in the main report or in the supplementary materials. Citing each report enables retrieval of relevant reports if desired.

Essential elements

- Provide references for each included report from which information on interpretability was collected.
- Present interpretability aspects of each OMI in a table or figure (considering a format that will facilitate comparison of characteristics across OMIs).

Example of item #23b

"Information on feasibility and information on interpretability can be found in online supplemental appendix 8 and online supplemental appendix 9, respectively."[18]

The following is an abridged version of Appendix 9 in the review by Elsman et al., 2022.[18]

Appendix 9. Information on interpretability of PROMs

**PROM – *subscale***	**Distribution of scores in the study population**	**Percentage of missing items or percentage of missing scores**	**Floor and ceiling effects**	**Scores and change scores available for relevant (sub)groups**	**Minimal important change (MIC) or minimal important difference (MID)**
DFS – *Daily activities*[42]				Healed ulcer: ~69, Current ulcer: ~63[a]	
DFS-SF – *Dependence/ daily life*[34,38,45,60,61]	Ref 45: mean=47.7, median =50.0, SD=29.3<br>Ref 38: mean=71.4, median =85.0, SD=32.9<br>Ref 60: mean=56.3, median=55.0, SD=25.7	Ref 45: 0.0-1.5%<br>Ref 38: 0%	Ref 45: 7.8% floor, 2.9% ceiling<br>Ref 38: 5% floor, 30% ceiling<br>Ref 60: 0.9% floor, 5.5% ceiling	Ref 34: Pre vs. post closure of target ulcer change score – study 1: +3.7; study 2 +10.0<br>Ref 38: Healed vs. unhealed ulcer Change score: +13.9<br>Ref 60: >1 complication: 44.7, 1 complication: 55.8, No complication: 69.5	

Item #23c: If applicable, present feasibility aspects for each included OMI.

Explanation: Reporting information on feasibility of the included OMIs facilitates authors' conclusions regarding the suitability of OMIs and informs readers in the selection of the most suitable OMI for a specific purpose. Reporting information on feasibility is particularly relevant if the rationale for the review is to select the most suitable OMI for a particular use (e.g., for use as a primary outcome in research, for use in clinical practice, or for inclusion in a core outcome set). Information on feasibility helps readers understand the ease of application of the OMI in its intended context of use.[64] Feasibility aspects might include type and ease of administration, length of the OMI, completion time, patient's required mental and physical ability, ease of standardization and score calculation, copyright, cost of an OMI, required equipment, and requirement for approval. Presenting feasibility aspects of each OMI in a table can facilitate comparison of characteristics across OMIs. This table can be included in the main report or in the supplementary materials. Citing each report enables retrieval of relevant reports if desired.

Essential elements

- Provide references for included report from which information on feasibility was collected.
- Present feasibility aspects of each OMI in a table or figure (considering a format that will facilitate comparison of characteristics across OMIs).

Example of item #23c

"Information on feasibility and information on interpretability can be found in online supplemental appendix 8 and online supplemental appendix 9, respectively."[18]

The following is an abridged version of Appendix 8 in the review by Elsman et al., 2022.[18]

Appendix 8. Information on feasibility of PROMs

PROM	Type and ease of administration	Length of instrument[a]	Response options included subscale	Completion time	Patient's required mental and physical ability level	Ease of score calculation	Copyright
DFS[42]	Self-report	11 subscales, 58 items: Leisure (5); Physical health (6); **Daily activities (6)**; Emotions (17); Noncompliance (2); Family (5); Friends (5); Treatment (4); Satisfaction (1); Positive attitude (5); Financial (2)	Daily activities: 1 = none of the time, 2 = a little bit of the time, 3 = some of the time, 4 = most of the time, and 5 = all of the time			Scores are based on the sum of items associated with a subscale if at least 50% of the items in a scale are completed. When necessary, raw item scores are reverse coded so that the minimum possible score (1) represents the worst quality of life, and the maximum possible score (5) represents the best quality of life (all items except in the positive attitude subscale). Each subscale is scored from 0 to 100, higher scores indicate better quality of life.	Johnson & Johnson Research & Development, LCC
DFS-SF[34,38,45,60,61]	Self-report/interview-based	6 subscales, 29 items: Leisure (5); **dependence/ daily life (5)**; negative emotions (6); physical health (5); worried about ulcers/feet (4); bothered by ulcer care (4)	Dependence/ daily life: 1 = none of the time, 2 = a little of the time, 3 = some of the time, 4 = most of the time, and 5 = all of the time	12.5 minutes for interview-based administration		Scores are based on the sum of items associated with a subscale if at least 50% of the items in a scale are completed. In case of item-level missing data <50%, the subscale score is calculated by substituting the mean item score for the missing item values. Raw item scores are reverse coded so that the	Johnson & Johnson Research & Development, LCC

Study characteristics

Item #24: Cite each included study report evaluating one or more measurement properties and present its characteristics.

Explanation: Reporting the details of the included studies allows readers to understand the characteristics of studies that have addressed the review question(s) and is therefore important for understanding the applicability of the review.[2] Characteristics of interest might include characteristics of the population (e.g., sample size, age, sex/gender, disease characteristics (e.g., disease, duration, severity)), characteristics of OMI administration (e.g., setting, country, language), response rate, measurement properties evaluated, funding source, and competing interests of study authors. Presenting the key characteristics of each study in a table or figure can facilitate comparison of characteristics across the studies.[2,109] This table can be included in the report or in an online supplement. Citing each study report enables retrieval of relevant reports if desired.[2]

Essential elements

- Provide references for each included report.
- Present the key characteristics (e.g., for the population, OMI administration, and evaluated measurement properties) of each study in a table or figure (considering a format that will facilitate comparison of characteristics across the studies).[2]
- If different studies on different measurement properties with different characteristics are described in one report, report key characteristics for each study separately.

Examples of item #24

Example 1: In a review examining the measurement properties of situational awareness instruments in healthcare providers,[108] the authors included a table combining the characteristics of included studies with characteristics of included OMIs (item #23a).

See *Example 1 of item #23a* for an abridged version of Table 1 in the review by Ghaderi et al., 2023.[108]

Example 2: In a review examining the measurement properties of measurement tools for mental health problems in children and adolescents with intellectual disability,[30] the authors included a table presenting for each included study the citation, country, description of the sample, disease characteristics, sample size, study design, rater and measurement properties assessed.

The following is an abridged version of Table 2 in the review by Halvorsen et al., 2023.[30]

Table 2 Overview of studies: study characteristics and psychometric data

Measure	Author, year	Country	Sample description	IQ/adaptive level	N	Study design	Rater	Psychometric properties
ABC								
	Brown et al. (2002)[a]	US	Special education 56% boys. Age range 6–22 years	FSIQ ≤ 80 indexed by school placement	601	Cross-sectional	Parent	Factor structure (EFA/CFA)<br>Internal consistency
	Chadwick et al. (2000)	UK	Special education. 62% boys. Age range 4–11 years	Severe ID defined by means of adaptive level	102	Cross-sectional	Parent (n = 102)<br>Teacher (n = 65)	Interrater agreement<br>Convergent validity (VABS)
	Freund & Reiss, 1991)	US	Outpatients. 69% boys. Age range 3–25 years	Borderline to severe<br>FSIQ: *M* = 53.0 (*SD* = 14.9)<br>Adaptive level: NR	110/94	Cross-sectional	Parent (n = 110)<br>Teacher (n = 94)	Factor structure (EFA)<br>Internal consistency<br>Test–retest<br>Interrater reliability
	Marshburn and Aman (1992)[a]	US	Special education. Gender frequency: NR. Age range 6–21 years	FSIQ ≤ 80. Indexed by school placement<br>FSIQ/adaptive level: NR	666	Cross-sectional	Teacher	Factor structure (EFA). Internal consistency<br>Norms
	Rojahn and Helsel (1991)	US	Inpatient psychiatric unit. 75% boys. Age range 3–23 years	Borderline to profound ID<br>FSIQ/adaptive level: NR	199	Follow-up	Direct care staff	Factor structure (EFA)<br>Internal consistency. Interrater reliability<br>Criterion validity
	Sansone et al. (2012)	US	Fragile X. 73% boys. Age range 3–25 years	FSIQ: *M* = 58.0 (*SD* = 18.3)	630	Cross-sectional	Parent/guardian	Factor structure (EFA/CFA)
ASEBA CBCL	Borthwick-Duffy et al. (1997)	US	Children with ID. 52% boys. Age range 8–20 years	Mild to profound ID<br>FSIQ: NR	67	Cross-sectional	Parent	Factor structure (EFA)

Risk of bias in studies

Item #25: Present assessments of risk of bias for each included study.

Explanation: For readers to understand whether the results of individual studies can be trusted, they need to know the risk of bias in results of each included study.[2,15] The best approach is to present tables or figures indicating the risk of bias for each study on a measurement property. This can be presented in the main manuscript or in supplementary files. Presentation of risk of bias ratings can be combined with extracted results of the studies and the ratings of the measurement properties against quality criteria (see item #26).

Essential elements

- Present tables or figures indicating the risk of bias of each study on a measurement property (considering a format that will facilitate understanding of risk of bias in studies in relation to the results).

Additional elements

- Consider presenting an explanation for suboptimal risk of bias ratings of each study on a measurement property (e.g., in brackets following the risk of bias rating, as footnotes in a table or in the main text).

Example of item #25

Example 1: In a review examining the measurement properties of diabetes-specific PROMs measuring physical functioning,[18] the authors presented a table combining the risk of bias ratings with the ratings of the measurement property (item #26). In the appendix, they provided a more extensive table, combining the risk of bias ratings with the results and ratings of measurement properties (item #26). The appendix also shows the synthesized results, consisting of the summarized or pooled result with the overall rating (item #27a), and the certainty of the evidence (item #28).

The following are abridged versions of Table 2 and Appendix 10 in the review by Elsman et al., 2022.[18]

Table 2 Results and quality of studies on measurement properties of diabetes-specific PROMs measuring physical functioning in people with type 2 diabetes

PROM—subscale	Structural validity	Internal consistency	Cross-cultural validity\ measurement invariance	Reliability	Measurement error	Criterion validity	Hypotheses testing for construct validity*	Responsiveness†
DFS—Daily activities[42]	Inadequate<br>?	Inadequate<br>?		Inadequate<br>Daily life: –<br>Dependence: +			a. Adequate 4+ / 1–<br>b. Very good ?	c. Doubtful<br>2–
DFS-SF study 1—Dependence/ daily life[34]	Inadequate<br>?	Very good<br>+		Inadequate<br>+			a. Adequate 2+ / 3–	c. Very good<br>1–
DFS-SF study 2—Dependence/ daily life[34]	Very good<br>+	Very good<br>+		Inadequate<br>+			a. Adequate 2+ / 3–	c. Very good<br>1+
DFS-SF Polish—Dependence/ daily life[45]		Very good<br>+	Inadequate<br>+				a. Very good 4+ / 3–;<br>b1. Inadequate 1+ / 3–;<br>b2. Doubtful ?	
DFS-SF Chinese—Dependence/ daily life[38]		Very good<br>+					a. Very good 5+ / 2–<br>b. Very good 1+ / 1–	
DFS-SF Greek—Dependence/ daily life[60]		Very good<br>+					a. Very good 4+ / 3–<br>b. Very good 14+ / 1–	
DFS-SF Spanish—Dependence/ daily life[61]	Inadequate<br>CFA: –<br>EFA: +	Very good<br>+		Doubtful<br>+			a. Adequate 4+ / 1–	c. Very good<br>1+
PRO-DM-Thai—Physical function[35]	Very good<br>+	Very good<br>+					b1. Inadequate 1–;<br>b2. Very good ?	
IWADL/APPADL—(Physical) activities of daily living[36]	Doubtful<br>+	Very good<br>+					b. Very good 12+ / 22–	
IWADL/APPADL—(Physical) activities of daily living[46]		Very good<br>+		Adequate<br>+	Doubtful<br>–			d. Very good<br>2+ / 1–

Appendix 10. Extensive results of studies on measurement properties

**PROM – *subscale***	**Structural validity**			**Internal consistency**			**Reliability**		
	**n**	**Meth qual**	**Result (rating)**	**n**	**Meth qual**	**Result (rating)**	**n**	**Meth qual**	**Result (rating)**
IWADL/APPADL – *(Physical) activities of daily living*[36]	349	Doubtful	(physical) activities of daily living (7 items): 73% variance explained; factor loadings 0.82-0.90; eigenvalue 5.1 (+)	349	Very good	(physical) activities of daily living: α=0.94 (+)			
IWADL/APPADL – *(Physical) activities of daily living*[46]				106	Very good	(physical) activities of daily living: α≥0.89[d] (+)	106	Adequate	(physical) activities of daily living: ICC agr=0.91 (+)
**Pooled or summary result (overall rating)**	**349**	**Low**	**(physical) activities of daily living (7 items) (+)**	**455**	**Low**[e]	**(physical) activities of daily living: α≥0.89 (+)**	**106**	**Moderate**	**(physical) activities of daily living: ICC agr=0.91 (+)**

**PROM – *subscale***	**Measurement error**			**Hypotheses testing for construct validity**<br>**a=comparison with other instruments**<br>**b=comparison between subgroups**			**Responsiveness**<br>**a=comparison to gold standard**<br>**b=comparison with other instruments**<br>**c=comparison between subgroups**<br>**d=before and after intervention**		
	**n**	**Meth qual**	**Result (rating)**	**n**	**Meth qual**	**Result (rating)**	**n**	**Meth qual**	**Result (rating)**
IWADL/APPADL – *(Physical) activities of daily living*[36]				b. 349	b. Very good	b. Results in line with 12 hypos (12+); results not in line with 22 hypos (22-)			
IWADL/APPADL – *(Physical)*	106	Doubtful	(physical) activities of daily				d. 40	d. Very good	d. Results in line with 2

activities of daily living[46]			living: SEM=6.3; SDC=17.5; MIC=9.8-13.6^k (-)						hypos (2+); results not in line with 1 hypo (1-)
**Pooled or summary result (overall rating)**	**106**	**Low**	**(physical) activities of daily living: SEM=6.3; SDC=17.5 ; MIC=9.8-13.6^k (-)**	**b. 349**	**b. High**	**b. 12+ and 22- (±)**	**d. 40**	**d. Low**	**d. 2+ and 1- (±)**

The authors also provide an explanation for common suboptimal risk of bias ratings in the main text, when discussing each measurement property.

"For the other PROMs [patient-reported outcome measures], the development was rated as inadequate, because the construct of the included physical functioning subscale was not clearly described or the PROM was not pilot tested. […] If studies had inadequate quality for structural validity or cross-cultural validity\measurement invariance, this was often due to small sample sizes. […] Reliability was evaluated for six PROMs or subscales. All studies with inadequate quality had a time interval that was considered to be too long (i.e., more than 4 weeks). […] Three studies were of inadequate quality, because they did not apply an appropriate statistical method to compare subgroups."[18]

Example 2: In a review examining the measurement properties of oral health assessments,[38] the authors assessed risk of bias using an old version of the COSMIN risk of bias checklist. To present the risk of bias of each study on a measurement property, the authors include tables showing the methodological quality of studies for each measurement property domain in combination with the results and a rating of the measurement property (item #26). Here, the table for measurement properties in the domain reliability is shown.

The following is a reproduced version of Table 5 in the review by Everaars et al., 2020.[38]

Table 5 Methodological quality of the measurement property "reliability" by the COSMIN and quality criteria of the measurement properties per assessment

Assessment	Study	Reliability								Raters
		Internal-consistency		Intra-rater reliability		Inter-rater reliability		Test-retest reliability		
		M	Q	M	Q	M	Q	M	Q	
ROAG	Andersson et al. (2002a) [18]					Good[a]	?/– (κ/κ^{w}: 0.45-0.84)[b]			Nurse/Dental hygienist
	Ribeiro et al. (2014) [37]			Good	+/– (κ^{w}: 0.38-0.88)					Community health workers
MDS	Arvidson-Bufano et al. (1996) [28]					Poor[a]	N.A.			Nurse/Dentist
	Blank et al. (1996) [27]					Poor[a]	N.A.			Nurse/Dentist
	Cohen-Mansfield (2002) [28]					Poor[a]	N.A.			Geriatricians/Dentist
	Hawes et al. (1995) [31]					Poor	N.A.			Nurses
MDS-HC	Morris et al. (1997) [35]					Good	+/– (κ^{w}: 0.57-0.7)			Nurses
OHAT	Chalmers et al. (2005) [17]			Fair	+ (ICC = 0.78) ? (κ: 0.51-0.80)[b]	Fair	+ (ICC = 0.74) ? (κ: 0.48-0.80)[b]	Poor	N.A.	Nurses
	Simpelaere et al. (2016) [38]			Poor	N.A.	Fair	+ (ICC = 0.96) ? (κ: 0.83-1.00)	Fair	+ (ICC = 0.81 & 0.78) ? (κ: 0.14-0.91)	Speech pathologists
THROAT	Dickinson et al.(2001) [19]			Good	+/– (κ^{w}: 0-0.96)	Good[a]	+/– (κ^{w}: 0.46-0.97)			Dental hygienist,/ stroke specialist nurse and staff Nurse
DHR	Fjeld et al. (2017) [29]			Fair	+ (κ: 0.7-0.8)	Fair[a]	? (κ: 0.4-0.8)			Dental hygienist and Nurse
MPS	Henriksen et al. (1999) [32]			Poor	N.A.	Poor[a]	N.A.			Dentist, 2 Dental Hygienist, and Nurse
BOHSE	Kayser-Jones et al. (1995) [33]					Fair[a]	-(r: 0.4-0.68) ? (κ: -0.02-0.82)[b]	Fair	+/– (r: 0.79-0.88)	Dentist and Nurses
	Lin et al. (1999) [34]					Fair[a]	? (κ: -0.018-0.519)[b]			Dentist and Nurses
OAS	Yanagisawa et al. (2017) [39]	Poor	N.A			Fair	? (κ: 0.25-0.90) +/- (ICC: 0.54-0.98)			Dental professionals and care workers

M = Assessment of methodological quality: "excellent", "good", "fair", "poor" by COSMIN. Q = criteria for measurement properties; + = positive rating;? = indeterminate rating; – = negative rating.
[a] Inter-rater reliability measurements have been performed by two different professions.
[b]Only kappas are reported instead of percent agreement because this reflects better methodological quality according to the COSMIN criteria
N.A. Not applicable was reported for the quality criteria when an article had poor methodological quality.

The authors also provide the reasons for poor methodological quality of each study in a table and explain common reasons for poor methodological quality in the main text.

"In total, five studies showed good methodological quality on at least one measurement property and 14 studies showed poor methodological quality on some of their measurement properties. An overview of the reasons for poor methodological quality is shown in Table 3. Below, the results on the methodological quality per measurement property will be described. […] all five studies that assessed content validity, scored poor on their methodological quality, mainly because the patient population was not involved in developing the oral health assessment and studies did not assess if the items comprehensively reflect the construct (i.e., "oral health") to be measured (see Table 3)."[38]

The following is a reproduced version of Table 3 in the review by Everaars et al., 2020.[38]

Table 3 Reasons for scoring poor methodological quality on the measurement property for assessing oral health per study

Study	Assessment	Measurement property	Reason for poor methodological quality
Andersson et al. (2002b) [25]	ROAG	Content validity	- Target population not involved<br>- Not assessed if all items together comprehensively reflect the construct to be measured
Arvidson-Bufano et al. (1996) [26]	MDS-RAI	Inter-rater reliability	- Small sample size<br>- Only percent agreement calculated
Blank et al. (1996) [27]	MDS-RAI	Inter-rater reliability	- Unclear how many patients the dentist assessed<br>- Only percent agreement is calculated<br>- Other important methodological flaws in design or execution of study
Chalmers et al. (2005) [10]	OHAT	Content validity<br>Criterion Validity<br>Test-retest	- Target population not involved<br>- Not assessed if all items together comprehensively reflect the construct to be measured<br>- Small sample size<br>- No ICC or correlation calculated
Cohen-Mansfield et al. (2002) [28]	MDS	Inter-rater reliability	- Small sample size<br>- No ICC or correlations calculated<br>- Other important methodological flaws in design or execution of study
Dickinson et al. (2001) [19]	THROAT	Content validity	- Target population not involved
Fjeld et al. (2017) [29]	DHR	Content validity	- Target population not involved
Hanne et al. (2012) [30]	ROAG	Cross-cultural validity	- Only forward translation
Hawes et al. (1995) [31]	MDS	Inter-rater reliability	- Only percent agreement is calculated
Henriksen et al. (1999) [32]	MPS	Intra-rater reliability<br>Inter-rater reliability	- Small sample size
Kayser-Jones et al. (1995) [33]	BOHSE	Content validity	- Target population not involved
Paulsson et al. (2008) [36]	ROAG	Criterion validity	- Other important methodological flaws in design or execution of study<br>- Correlations or AUC not calculated<br>- Sensitivity and specificity not calculated
Simpelaere et al. (2016) [38]	OHAT	Intra-rater reliability	- Small sample size<br>- Only percent agreement is calculated
Yanagisawa et al. (2017) [39]	OAS	Criterion-validity	- No factor analysis performed and no reference to another study

Results of individual studies

Item #26: For all measurement properties, present for each study: (a) the reported result and (b) the rating against quality criteria, ideally using structured tables or plots.

Explanation: Presenting results from individual studies on measurement properties facilitates understanding of each study's contribution to conclusions about an OMI. It also allows reuse of the data by others seeking to perform additional analyses or perform an update of the review.[2] There are different ways of presenting results of individual studies (e.g., in the main text, tables, figures, or forest plots), and it might depend on the measurement property what format would be preferred. Ideally, results from different studies on the same measurement properties of the same OMI should be presented and grouped together.

Results of each study should be rated against predefined quality criteria, and this rating should be reported. For example, the criterion for internal consistency is to have a Cronbach's alpha of at least 0.70 for a unidimensional scale. If a study finds a Cronbach's alpha of 0.65 in a unidimensional scale, the study is rated as insufficient. Ratings can be combined with the presentation of results in tables or figures.

Authors may choose to present only the ratings in the main manuscript, because the results are too extensive. In that case, results accompanied by a rating can be presented in the supplementary files. Presentation of reported results and/or ratings against quality criteria can also be combined with risk of bias ratings.

Essential elements

- For each study, report quantitative or qualitative results on each measurement property, ideally grouped per OMI.
- Accompany each quantitative or qualitative result of a study with a rating about the quality of the results, determined based on predefined quality criteria for good measurement properties.
- If applicable, indicate which results were not reported directly in the included report and had to be computed or estimated from other information (e.g., as footnotes in a table).[2]

Additional elements

- If data are presented visually or reported in the main text (or both), consider also presenting a tabular display of the results to aid with independent interpretation of the data.[2]

Example of item #26

Example 1: In a review examining the measurement properties of oral health assessments,[38] the authors presented the results of individual studies with a rating against predefined quality criteria. The authors combined this information with a presentation of the risk of bias (item #18), assessed using an old version of the COSMIN risk of bias checklist.

See *Example 2 of item #25* for a reproduced version of Table 5 in the review by Everaars et al., 2020.[38]

Example 2: In a review examining the measurement properties of diabetes-specific PROMs measuring physical functioning,[18] the authors presented a table combining the ratings of the measurement property with the risk of bias. In the appendix, they provided a more extensive table, combining the results and ratings of measurement properties with the risk of bias ratings (item #18). The appendix also shows the synthesized results, consisting of the summarized or pooled result with the overall rating (item #20b), and the certainty of the evidence (item #22).

See *Example 1 of item #25* for abridged versions of Table 2 and Appendix 10 in the review by Elsman et al., 2022.[18]

Example 3: In a review examining the measurement properties of teacher proxy-report tools of children's physical literacy,[24] the authors presented the results of each study and its rating against predefined quality criteria for each measurement property.

The following is an abridged version of Table 3 in the review by Essiet et al., 2021.[24]

Table 3 Evaluating results for measurement properties against COSMIN's updated criteria for good measurement properties

Instrument name	Citation	Structural validity (rating)	Criterion validity (rating)	Cross-cultural validity (rating)	Construct validity (rating)	Internal consistency (rating)	Reliability (rating)
**Single Domain Measures**							
Motor Observation Questionnaire for Teachers (MOQ-T)	Schoemaker et al. [45]	–	With Movement Assessment Battery for Children test $r = 0.57$, $p < 0.001$; AUC = 0.77, CI: 0.71–0.84; Sensitivity = 80.5%; Specificity = 62% for cut-off score > 35 (+)	–	*Convergent* With Developmental Coordination Disorder-Questionnaire $r = -0.64$, $p < 0.001$ (1+) *Discriminant* Children in referred group (49.0, SD = 11.0) versus comparison group (30.2, SD = 11.2), $F(1,182) = 130.442$, $p < 0.001$ (1?)	–	–
	Giofrè et al. [46]	EFA: 2 factors accounting for 58.26% of total variance CFA: χ^2 (134) = 269.01, RMSEA = 0.05, SRMR = 0.05, CFI = 0.99, NNFI = 0.99, AIC = 343.01 (+)	–	–	–	Cronbach's α 0.95 (?)	–

Results of syntheses

Item #27a: Present results of all syntheses conducted. For each measurement property of an OMI, present: (a) the summarized or pooled result and (b) the overall rating against quality criteria.

Explanation: Users of reviews rely on the reporting of all syntheses conducted so that they have complete and unbiased evidence on which to base their decisions.[2] As in other fields, selectively reporting results in systematic reviews is a risk.[110] Transparent reporting of all results is encouraged. In systematic reviews in which measurement properties are evaluated, this is sometimes done by a statistical synthesis (e.g., meta-analyses or pooling results), but more often by qualitatively summarizing the results (e.g., giving a range of the results).[7] It is important to present both the summarized or pooled result and the overall rating against quality criteria.[7] For multi-dimensional OMIs, summarized results and ratings should be provided for all subscales separately.[7]

Essential elements

- Report results of all syntheses described in the protocol and all syntheses conducted that were not pre-specified.[2]
- If qualitative synthesis was conducted, report the summarized result (e.g., a range of the results, the number of hypotheses confirmed)
- If meta-analysis was conducted, report for each:[2]
    - the pooled estimate and its precision (such as standard error or 95% confidence/credible interval).
    - measures of statistical heterogeneity (such as τ2, I2, prediction interval).
    - pooled sample size across studies included.
- Report the overall rating against quality criteria used at a synthesis level.
- If an OMI is multi-dimensional, report results per subscale relevant to the outcome domain of interest.[7]

Example of item #27a

Example 1: "Construct validity via hypothesis testing was assessed in three studies for the *PROMIS-PF* item bank and in two studies for the UE [upper extremity] subdomain. For convergent validity and known-groups validity together, 12 out of 15 hypotheses (80%) for unique correlations/group differences were correct for the PF item bank, and 4 out of 5 (80%) for the UE subdomain. Correlations for some instruments (i.e., *HAQ-DI*, *SF-36-PF10* and *MHQ-ADL*) were determined in more than one study. Since these showed consistent positive results in study populations of adequate sample size, even without statistical pooling these correlations clearly confirmed the hypothesis and contributed to the high quality evidence for sufficient construct validity for both the *PROMIS-PF* item bank and the UE subdomain."[37]

Example 2: In a review examining the measurement properties of patient- and proxy-reported outcomes targeted at children with impairment of the upper limb,[36] the authors presented a table combining the summarized results and the overall ratings of each measurement property with the certainty of the evidence with (item #28).

The following is an abridged version of Table 4 in the review by Kalle et al., 2022.[36]

Table 4 Synthesized evidence

PROM (refs)	Measurement property	Summarized result	Overall rating*	Quality of evidence§
ABILHAND-Kids (Original version) [23, 33, 40–42, 48]	Structural validity	INFIT mean square range 0.66–1.18; OUTFIT mean square range 0.45–1.55	–	Moderate
	Internal consistency	Person separation reliability coefficient 0.94	?	
	Reliability	ICC range = 0.81–0.91	+	Moderate
	Measurement error	SEM = 1.7; SDD_{95} = 6.7; SDD_{95}/range = 0.16; SEM = 1.9; SDD_{90} = 4.8; SDD/range = 0.11; LOA = -2.06–1.40	?	
	Construct validity	9 out of 20 hypotheses confirmed	±	
	Responsiveness	RM ANOVA F = 29.89, p < 0.001; Effect size T1vsT2 = 0.916, T2vsT3 = 0.158; Correlation changes measured by PEDI and ABILHAND-Kids Spearman r = 0.430, p = 0.003; Correlation changes measured by AHA and ABILHAND-Kids Pearson r = –0.104, p = 0.493	?	
ABILHAND-Kids (Ukrainian version) [27]	Structural validity	Standardized residuals range = -2.19–1.58	–	Moderate
	Internal consistency	Person separation index = 0.95	?	
	Cross-cultural validity	3 major DIF's were observed across countries (Ukrainian versus Belgian cohort)	–	Moderate
ABILHAND-Kids (Danish version) [28]	Structural validity	TLI = 0.98; CFI = 0.98; RMSEA = 0.07; SRMR = 0.07 Fit residuals (z) range = -2.178–2.170	–	Moderate
	Internal consistency	Cronbach's alpha = 0.96	?	
	Measurement invariance	1 non-uniform DIF was observed across age groups	–	Moderate
	Reliability	ICC2.1 = 0.97 (95% CI 0.95–0.98)	+	High
	Measurement error	SE = 0.5; LOAs range: –4.8–5.5; SDC = 5.15 points	?	
ABILHAND-Kids (Turkish version) [29]	Structural validity	Residual (z) range = -1.636–1.934	?	
	Internal consistency	Cronbach's alpha = 0.94	?	
	Measurement invariance	No DIF was observed	+	Very low
	Reliability	ICC = 0.98 (95% CI 0.98–1.00)	+	Very low
	Construct validity	2 out of 2 hypotheses confirmed	+	High

* The results of the different studies on a particular measurement property of a PROM were qualitatively summarized and then rated against the updated criteria for good measurement properties: – = insufficient; + = sufficient; ± = inconsistent; ? = indeterminate

§ The quality of the evidence was graded by using a modified GRADE approach

Example 3: In a review examining the measurement properties of diabetes-specific PROMs measuring physical functioning,[18] the authors included an appendix showing the summarized result for each measurement property, including a rating for the summarized result. It also shows the certainty in the body of evidence (item #28).

See *Example 1 of item #25* for an abridged version of Appendix 10 in the review by Elsman et al., 2022.[18]

The authors also discuss the results of syntheses in the main text.

"Considering results of the PROM [patient-reported outcome measure] development studies, content validity studies if both were at least doubtful, and the reviewer ratings, the content validity of the *DFS*, *DFS-SF*, and *IWADL* for measuring physical functioning was considered sufficient, but often with very low-quality evidence. […] Sufficient structural validity *and* internal consistency was found for the *DFS-SF*, *PRO-DM-Thai*, *IWADL*, and Chinese Cardiff Wound Impact Schedule (C-CWIS)."[18]

Example 4: In a review examining the content validity of PROMs specifically developed to measure (aspects of) health-related quality of life in people with type 2 diabetes,[19] the authors included a

table showing the rating for each aspect of content validity (there are often no summarized results for content validity), together with the certainty in the body of evidence (item #28).

The following is an abridged version of Table 3 in the review by Terwee et al., 2022.[19]

Table 3 Content validity (relevance, comprehensiveness, comprehensibility) of disease-specific patient-reported health outcome measures developed for patients with type 2 diabetes mellitus (PROMs with positive ratings for content validity are presented in green)

PROM	Subscale	Number of items	Relevance		Comprehensiveness		Comprehensibility			Comments
			OVERALL RATING	QUALITY OF EVIDENCE	OVERALL RATING	QUALITY OF EVIDENCE	OVERALL RATING	QUALITY OF EVIDENCE		
			+ / - / ?	High, moderate, low, very low	+ / - / ?	High, moderate, low, very low	+ / - / ?	High, moderate, low, very low	Language *	
SYMPTOM STATUS										
DISEASE-SPECIFIC SYMPTOMS										
C-CWIS[59]	Physical symptoms and everyday living	12	-	very low	-	very low	+	low	CH	three questions are not related to health, no questions on discomfort other than pain (e.g. itching, throbbing)
DFS[29]	Physical health	6	+	very low	+	very low	+	moderate	EN	
DFS-SF[30]	Physical health	5	+	low	+	Very low	+	moderate	EN	
DIMS[75]	Diabetes-specific symptoms	6	±	very low	-	very low	±	very low	EN	Questions about spontaneous hypoglycemia are missing
	Non-specific symptoms	11	±	very low	+	very low	±	very low	EN	

Item #27b: If applicable, present results of all investigations of possible causes of inconsistency among study results.

Explanation: Presenting results from all investigations of possible causes of inconsistency among study results is important for users of reviews and for future research.[2] For users, understanding the factors that may, and equally, may not, explain variability in measurement properties' results, may inform decision making.[2] Similarly, presenting all results is important for designing future studies, as the results may help to generate hypotheses about potential modifying factors that can be tested in future studies.[2] Selective reporting of the results leads to an incomplete representation of the evidence that risks misdirecting decision making and future research.[2]

Essential elements

- If investigations of possible causes of inconsistency were conducted:[2]
    - present results of all possible causes of inconsistency.
    - identify the studies contributing to each subgroup.
- If qualitative methods were used to investigate inconsistency, describe the results observed. For example, present a table that groups study results by study quality, subpopulations, study characteristics or contextual factors and comment on any patterns observed.[111]
- If subgroup analysis was conducted, report for each analysis within each subgroup, the summary estimates, their precision if applicable (such as standard error or 95% confidence/credible interval) and descriptions of inconsistency. Results from subgroup analyses might usefully be presented graphically.[2]

Example of item #27b

Example 1: "The convergent validity of the *ASQOL* questionnaire is weak to good. The summary r values of the association with *ASQOL* questionnaire and *BASDAI* were 0.78 (95% CI 0.74 to 0.82) and 0.54 (95% CI 0.47 to 0.61) in the Europe and regions beyond Europe. Subgroup analysis demonstrated that the *ASQOL* questionnaire was more validated and reliable to evaluate the QoL [quality of life] in the Europe than other regions.[34]

Example 2: "Two studies of excellent and good quality concluded that, over the total study sample, the *BIS* has a one-factor solution. In subgroup analyses, a two-factor structure was found among breast cancer patients after mastectomy and breast cancer patients after surgery with immediate breast reconstruction. Three fair quality studies also reported a one-factor solution and one fair quality study reported a two-factor solution among breast cancer patients after breast-conserving surgery (BCS) or mastectomy. […] Based on these findings, structural validity of the *BIS* overall was rated sufficient (+) because two studies of at least good quality and three studies of fair quality support unidimensionality of the scale. It should be noted that in some studies, a two-factor solution was also found.[20]

Example 3: In In a review examining the validity and reliability of inertial measurement units on lower extremity kinematics during running,[101] the authors performed subgroup analysis to explore potential sources of heterogeneity in intraclass correlation coefficients (ICCs). Because the intraclass

correlation coefficient was not normally distributed, they transformed the ICC to Fisher's Z and back transformed those to ICCs when discussing the results.

"Subgroup analysis showed no significant effect of running speed on the validity for stance time derived from IMUs [inertial measurement units] (p = 0.54), while IMUs at the shoe (ICC (95% CI) = 0.929 (0.869, 0.961), I2 = 71%) showed higher agreement compared to at the waist (ICC (95% CI) = 0.226 (− 0.282, 0.641), I2 = 94%) (p < 0.001) (Fig. 2)."[101]

The following is a reproduced version of Figure 2 in the review by Zeng et al., 2022.[101]

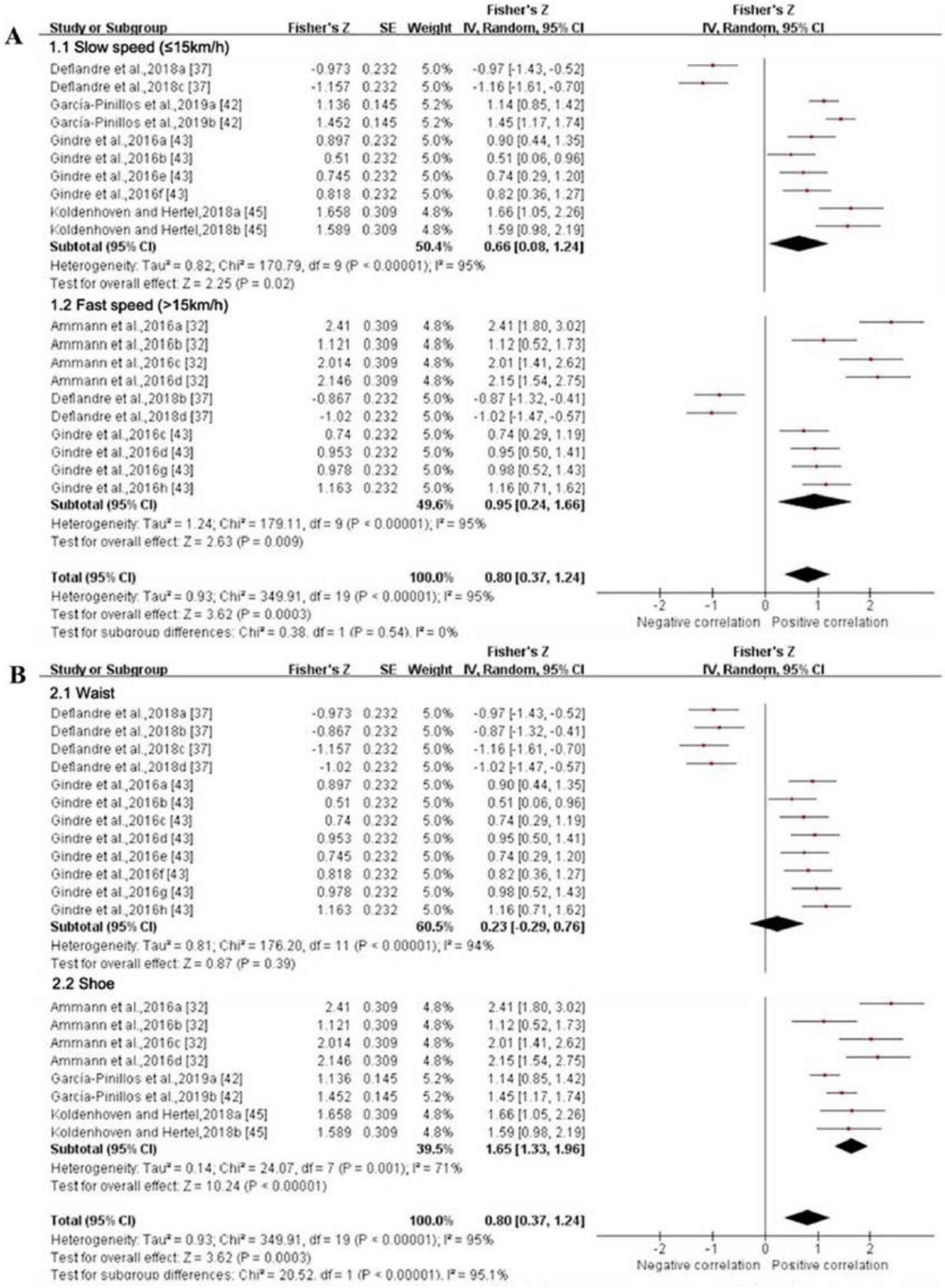

Item #27c: If applicable, present results of all sensitivity analyses conducted to assess the robustness of the synthesized results.

Explanation: Presenting results of sensitivity analyses conducted allows readers to assess how robust the synthesized results were to decisions made during the review process.[2] Reporting results of all sensitivity analyses is important; presentation of a subset, based on the nature of the results, risks introducing bias due to selective reporting.[2] Sensitivity analyses for the subset of interest can best be reported in a summary table.[98]

Essential elements

- If any sensitivity analyses were conducted:[2]
    - report the results for each sensitivity analysis.
    - comment on how robust the main analysis was given the results of all corresponding sensitivity analyses.

Additional elements

- If any sensitivity analyses were conducted, consider:[2]
    - presenting results in tables that indicate: (i) the summarized result, a measure of precision (and potentially other relevant statistics, for example, I2 statistic) and contributing studies for the original analysis; (ii) the same information for the sensitivity analysis; and (iii) details of the original and sensitivity analysis assumptions.
    - presenting results of sensitivity analyses visually (e.g., using forest plots).

Example of item #27c

"Data from three MQ [moderate quality] studies suggested that the validity for flight time measured by IMUs [inertial measurement units] was poor with no statistical significance (ICC [intraclass correlation coefficient] (95% CI) = 0.371 (− 0.110, 0.711), I2 = 95%, p = 0.13). […] The sensitivity analysis showed that after excluding the study of Deflandre et al., the I2 reduced (I2 = 0%), summary ICC value increased (ICC (95% CI) = 0.774 (0.716, 0.818), p < 0.001). Sensitivity analysis showed that the results were unstable."[101]

Certainty of evidence

Item #28: Present assessments of certainty (or confidence) in the body of evidence for each measurement property of an OMI assessed.

Explanation: For readers to understand whether the synthesized result is trustworthy, they need to know the certainty or confidence in the body of evidence for each measurement property of an OMI.[7] An important feature of systems for assessing certainty, such as GRADE, is explicitly reporting of the level of certainty (or confidence) in the evidence.[2,42,112] Evidence summary tables, such as Summary of Findings or Summary of Measurement Properties tables,[65,113] are an effective and efficient way to report assessments of the certainty of evidence.[2,112] Reviewers can also report the level and its justification in the body of the manuscript.

Essential elements

- Report the overall level of certainty in the body of evidence (such as high, moderate, low, or very low) for each synthesized result.[2]
- Communicate certainty in the evidence wherever synthesized results are reported (that is, abstract, evidence summary tables, results, conclusions).[2] Use a format appropriate for the section of the review.[2] For example, in the main text, certainty might be reported explicitly in a sentence (such as "Moderate-certainty evidence indicates that…") or in brackets alongside a pooled measurement property result (such as "[pooled ICC 0.86, 95% CI 0.78 to 0.95; 2 studies, 181 participants; moderate certainty evidence]").

Additional elements

- Consider including evidence summary tables, such as Summary of Findings or Summary of Measurement Properties tables.[2,65,113]
- Consider providing an explanation of reasons for grading down the certainty of evidence (such as in the main text, in tables after the level of certainty, or in footnotes to an evidence summary table). Explanations for each judgment should be concise, informative, relevant to the target audience, easy to understand, and accurate (that is, addressing criteria specified in the methods guidance).[2,114] Use a format appropriate for the section of the review. For example, in the main text, certainty might be reported explicitly in a sentence (such as "Moderate-certainty evidence (downgraded for risk of bias) indicates that…").[2]

Example of item #28

Example 1: In a review examining the measurement properties of patient-reported outcome measures following knee replacement,[29] the authors presented a table combining the certainty of the evidence with the overall ratings of the measurement property (item #20b). The authors also report the overall rating with the certainty of the evidence in the main text.

"The quality of the evidence for measurement properties of the included PROMs [patient-reported outcome measures] is provided in table 7. […] The only measurement property to receive a 'sufficient' rating was reliability for both the *KOOS* and the *LEAS*, supported by 'low' and 'moderate' quality evidence, respectively."[29]

The following is a reproduced version of Table 7 in the review by Sabah et al., 2021.[29]

Table 7 Quality of the evidence for measurement properties of the PROMs						
	**KOOS**		**LEAS**		**WOMAC**	
	**Overall rating**	**Quality of evidence**	**Overall rating**	**Quality of evidence**	**Overall rating**	**Quality of evidence**
	**+/–/?**	**High, moderate, low, very low**	**+/–/?**	**High, moderate, low, very low**	**+/–/?**	**High, moderate, low, very low**
Structural validity	–	Very low	N	N	N	N
Internal consistency	?	Moderate	N	N	N	N
Cross-cultural validity	?	Very low	N	N	N	N
Measurement invariance	?	Very low	N	N	N	N
Reliability	+	Low	+	Moderate	N	N
Measurement error	?	Low	?	Very low	N	N
Criterion validity	N	N	N	N	N	N
Construct validity	–	Low	–	Very low	?	Very low
Responsiveness	N	N	?	Very low	?	Very low

+ = sufficient, – = insufficient, ? = indeterminate.
KOOS, Knee Injury and Osteoarthritis Outcome Score; LEAS, Lower Extremity Activity Scale; N, not assessed; PROM, patient-reported outcome measure; WOMAC, Western Ontario and McMaster Universities Arthritis Index.

Example 2: In a review examining the measurement properties of patient- and proxy-reported outcomes targeted at children with impairment of the upper limb,[36] the authors presented a table combining the certainty of the evidence with the summarized results and the overall ratings of the measurement property (item #27a).

See *Example 2 of item #27a* for an abridged version of Table 4 in the review by Kalle et al., 2022.[36]

Recommendations

Item #29: If appropriate, make recommendations for suitable OMIs for a particular use.

Explanation: Systematic reviews of OMIs are conducted for a variety of reasons (e.g., to select the best available OMI for a particular use, to provide an overview of the quality of available OMIs, etc.) and as such might make recommendations regarding the suitability of OMIs. Users of systematic reviews might use the results to select an OMI. Therefore, authors could report recommendations for the suitability of OMIs for a particular use if these are made. Although some systematic authors might believe that making recommendations for policy and practice is beyond the scope of a systematic review, others believe that providing recommendations on the suitability of OMIs for particular uses (e.g., health-care setting, research setting, conditions/diagnoses, follow-up timing, etc.) might help users in selecting an OMI and standardization of measurements.

Essential elements

- If recommendations on the suitability of OMIs for a particular use are made, report which OMIs can be recommended and/or which OMIs cannot be recommended.

Additional elements

- Consider reporting possible limitations for each of the recommended OMIs, e.g., in content, target population, feasibility, interpretability, or measurement properties.

Example of item #29

Example 1: "The *DFS-SF* and *IWADL* had sufficient relevance, comprehensiveness, and comprehensibility, and at least low-quality evidence for sufficient internal consistency, and can thus be considered for use in research and clinical practice. Both also had sufficient reliability, but measurement error of the IWADL was insufficient. The *DFS-SF* and *IWADL* had inconsistent responsiveness, with high-quality evidence for the subscale of the *DFS-SF*. This limitation should be taken into account when considering using the *DFS-SF* and *IWADL*."[18]

Example 2: "The combined rating of the evidence was supportive of a provisional endorsement of both *MHQ* subscales as core OMI […]. The working group noted the need to re-assess clinical trial discrimination in future clinical trials on their research agenda. *AUSCAN* received a provisional endorsement to serve as a second measure of function […]. While *AUSCAN* function may have better metric properties than *MHQ*, the working group felt that due to important feasibility issues (i.e., not available in public domain, costs associated with use of questionnaire), this instrument could not be recommended as a mandatory instrument to measure function in all hand OA [osteoarthritis] trials."[35]

Discussion

Discussion

Item #30a: Provide a general interpretation of the results in the context of other evidence.

Explanation: Discussing how the results of the review relate to other relevant evidence should help readers interpret the findings.[2] For example, authors might compare the current results to results of other similar systematic reviews (such as reviews that addressed the same question using different methods or that addressed slightly different questions) and explore possible reasons for discordant results.[2] Similarly, authors might summarize additional information relevant to decision makers that was not explored in the review,[2] such as evidence of patient and clinician preferences, including the acceptability and feasibility of using particular OMI in specific populations and settings.

Essential elements

- Provide a summary of the key findings in relation to the rationale and objective of the review.
- Provide a general interpretation of the results in the context of other evidence.[2]

Example of item #30a

Example 1: "No single tool reported all nine psychometric properties outlined by the COSMIN methodology. Measurement properties frequently reported included construct validity, structural validity, and internal consistency. Content validity and cross-cultural validity were the most rarely reported. No studies reported measurement error and responsiveness. These mirror findings of a recently published review of motor competence assessments for children and adolescents, which highlighted that construct validity was frequently reported whereas content validity was the least evaluated psychometric property."[24]

Example 2: "Musculoskeletal disorders account for one-third of all reviews on the COSMIN database. At least three reviews have evaluated the measurement properties of PROMs [patient-reported outcome measure] following primary knee replacement. These studies found that many PROM instruments had limited evidence to support their measurement properties, justifying the need for further research. We are not aware of previous reviews that have examined the measurement properties of PROMs following discretionary revision knee replacement. While many of the goals from discretionary revision knee replacement are shared with primary knee replacement, there are important differences in the patient populations and disease processes being treated and the surgical interventions themselves. […] As such, the evidence for PROMs developed in primary knee replacement cannot necessarily be assumed to be transferable across."[29]

Item #30b: Discuss any limitations of the evidence included in the review.

Explanation: Discussing the completeness, applicability, and uncertainties in the evidence included in the review should help readers interpret the findings appropriately.[2] For example, authors might acknowledge that they identified few eligible studies or studies with a small number of participants, leading to imprecision; have concerns about risk of bias in studies or missing results; or identified studies that only partially or indirectly address the population of interest, leading to concerns about their relevance and applicability to particular patients, settings, or other target audiences.[2] The assessments of certainty (or confidence) in the body of evidence (item #22) can support the discussion of such limitations.[2]

Essential elements

- Discuss any limitations of the evidence included in the review.[2]

Example of item #30b

Example 1: "Also for other measurement properties, information was sometimes reported poorly or unclear. Thus, as a team, we had to make decisions on how to value the information."[18]

Example 2: "There were a number of limitations in the studies reviewed. First, the number of studies examining self-report measures of exercise designed to be used within an eating disorder population is small. Only 12 studies were found that met inclusion criteria. In addition, this number was not distributed evenly between the tests, with only three studies examining the *EED*. Results pertaining to the quality of the *CET* and *EED* should therefore be interpreted with caution. Second, sample sizes varied significantly in the included studies. Some studies had small sample sizes and did therefore not meet the recommended criteria of 10 participants per item or more than 1000 participants for factor analysis."[50]

Example 3: "One of the main limitations [of the included studies] is represented by the fact that the included studies were only a few, very heterogeneous, with small samples and considerable differences in the age range; moreover, studies lacked in reporting the complete characteristics of the patients (as for example, the Gross Motor Function Classification System data), which are suggested to be described in future papers in order to allow the assessment of external validity of the findings."[43]

Item #30c: Discuss any limitations of the review processes used.

Explanation: Discussing limitations, avoidable or unavoidable, in the review process should help readers understand the trustworthiness of the review findings.[2] For example, authors might acknowledge the decision to restrict eligibility to studies in English only, search only a small number of databases, have only one reviewer screen records or collect data, or not contact study authors to clarify unclear information.[2] They might also acknowledge that they were unable to access all potentially eligible study reports or to carry out some of the planned synthesis because of insufficient data.[2] While some limitations may affect the validity of the review findings, others may not.[2]

Essential elements

- Discuss any limitations of the review processes used and comment on the potential impact of each limitation.[2]

Example of item #30c

"This study is not without limitations. Only studies published in English Language were included, due to our limited resources, time and expertise in non-English languages. Studies with English abstracts and non-English full text were also excluded because when it is not possible to obtain a translation, extracting all the information needed to meaningfully inform the systematic review based on the abstract only is difficult. Therefore, some findings may have been overlooked. Furthermore, because of the lack of rigorous peer-review, grey literature including conference, poster abstracts, dissertations, and tool manuals were excluded. As such, it is possible that some measurement properties (e.g., content validity) were reported within tool manuals."[24]

Item #30d: Discuss implications of the results for practice, policy, and future research.

Explanation: There are many potential end users of systematic reviews of OMIs (such as researchers, healthcare providers, patients, insurers, and policy makers), each of whom will want to know what actions they should take given the review findings.[2] Systematic reviews of OMIs are often conducted to select the most suitable OMI for a particular use, or to foster standardization.[7] As such, authors might discuss the implications for practice and policy with respect to the suitability of OMIs. Moreover, authors might clarify the impact of results found for different measurement properties, the potential effects on different contexts of use, and how the interpretation of the most important results of the review might lead different people to make different decisions. In addition, rather than making recommendations for practice or policy that apply universally, authors might discuss factors that are important in translating the evidence to different settings and factors that may modify measurement properties' results.

Explicit recommendations for future research – as opposed to general statements such as "More research on this question is needed" – can better direct the questions future studies should address and the methods that should be used.[2] For example, authors might consider describing the type of understudied participants who should be enrolled in future studies, specific OMIs or measurement properties that could be evaluated or that should not be evaluated further, and ideal study design features to employ.

Essential elements

- Discuss implications of the results for practice and policy.[2]
- Make explicit recommendations for future research.[2]

Example of item #30d

Example 1: "Of the disease-specific scales that were rated positively for both aspects of validity, the *HAQ* received the most favorable overall evaluation. Owing to its longstanding and extensive use in RA [rheumatoid arthritis], the measurement properties of the *HAQ* have been exhaustively studied. This review showed that it has predominantly favorable measurement properties that have been studied with adequate methodological rigor. The *HAQ* met the standards we set for responsiveness and its test-retest reliability was found to be very high in a sample of stable patients, indicating that the scale is appropriate for evaluative purposes (i.e., to track physical functioning over time), both at the group level and at the individual level. However, one important limitation of the *HAQ* is that multiple studies noted a considerable group of patients scoring the best possible score. Therefore, it may not be the most appropriate scale for use in patient populations with relatively good functional capacity, since it cannot measure improvement in a substantial proportion of patients."[39]

Example 2: "In the present review, only six studies described the PROM [patient-reported outcome measure] development process and this was only briefly presented. It is hard to tell whether the PROM development process had not been properly carried out or was just not reported. Detailed information about the PROMs development process should be described in future research."[32]

Example 3: "The measurement properties that have not been evaluated for various PROMs [patient-

reported outcome measures] could be evaluated in future studies. However, it is not very useful to study these measurement properties for a PROM with insufficient content validity. To measure physical functioning in a valid way, a PROM needs to contain items referring to the functioning of one's upper extremities, lower extremities or central regions, or relevant activities of daily living for people with type 2 diabetes and should not contain items that are not related to physical functioning or that lack key aspects of physical functioning. Only the Dependence/Daily Life subscale of the *DFS-SF* and the *IWADL* fulfill these requirements and are worthwhile to be subject of future validation studies."[18]

Example 4: "Our review suggests that licensure OSCEs [Objective Structured Clinical Examinations] for national professional program graduates have not been justified by formal research studies or by international practice standards. From a policy perspective this means that licensure OSCEs for national graduates could be discontinued to reduce the burden on new graduates entering the profession while maintaining public protection. However, while the usefulness of the OSCE appears limited from the current results, the undue burden is not as certain and should be evaluated. Furthermore, the value for international graduates requires further investigation. The evidence is more supportive of the use of OSCEs during professional training and yet the evidence has many gaps. Further research on their measurement properties, how they are best constructed, how they should be distributed across the curriculum, optimal methods of scoring and interpretation, their uses as formative and summative assessment, rater effects, and relationships to performance in clinical settings are all avenues of needed investigation."[115]

References

1. Page MJ, McKenzie JE, Bossuyt PM, et al. The PRISMA 2020 statement: an updated guideline for reporting systematic reviews. *International journal of surgery*. 2021;88:105906.
2. Page MJ, Moher D, Bossuyt PM, et al. PRISMA 2020 explanation and elaboration: updated guidance and exemplars for reporting systematic reviews. *bmj*. 2021;372
3. Higgins Jpt T, Chandler J, Cumpston M, Li T, Page M, Welch V. *Cochrane Handbook for Systematic Reviews of Interventions version 6.3 (updated February 2022)*. Cochrane; 2022. www.training.cochrane.org/handbook
4. Gates M, Gates A, Pieper D, et al. Reporting guideline for overviews of reviews of healthcare interventions: development of the PRIOR statement. *bmj*. 2022;378
5. Salameh J-P, Bossuyt PM, McGrath TA, et al. Preferred reporting items for systematic review and meta-analysis of diagnostic test accuracy studies (PRISMA-DTA): explanation, elaboration, and checklist. *bmj*. 2020;370
6. Butcher NJ, Monsour A, Mew EJ, et al. Guidelines for reporting outcomes in trial reports: the CONSORT-Outcomes 2022 extension. *JAMA*. 2022;328(22):2252-2264.
7. Prinsen CA, Mokkink LB, Bouter LM, Alonso J, Patrick DL, De Vet HC, Terwee CB. COSMIN guideline for systematic reviews of patient-reported outcome measures. *Quality of life research*. 2018;27:1147-1157.
8. Mokkink LB, Boers M, Van Der Vleuten C, et al. COSMIN Risk of Bias tool to assess the quality of studies on reliability or measurement error of outcome measurement instruments: a Delphi study. *BMC medical research methodology*. 2020;20:1-13.
9. Walton MK, Powers III JH, Hobart J, et al. Clinical outcome assessments: conceptual foundation—report of the ISPOR clinical outcomes assessment–emerging good practices for outcomes research task force. *Value in Health*. 2015;18(6):741-752.
10. OMERACT. *The OMERACT Handbook for establishing and implementing core outcomes in clinical trials across the spectrum of rheumatologic conditions*. 2021. Accessed March 2023. https://omeract.org/wp-content/uploads/2021/12/OMERACT-Handbook-Chapter-Final-June_2_2021.pdf
11. Schünemann H, Brożek J, Guyatt G, Oxman A. Handbook for grading the quality of evidence and the strength of recommendations using the GRADE approach. *Updated October*. 2013;2013
12. Mokkink LB, Terwee CB, Patrick DL, et al. The COSMIN study reached international consensus on taxonomy, terminology, and definitions of measurement properties for health-related patient-reported outcomes. *Journal of clinical epidemiology*. 2010;63(7):737-745.
13. Prinsen CA, Vohra S, Rose MR, et al. How to select outcome measurement instruments for outcomes included in a "Core Outcome Set"–a practical guideline. *Trials*. 2016;17(1):1-10.
14. Boutron I, Page M, Higgins J, Altman D, Lundh A, Hróbjartsson A. Chapter 7: considering bias and conflicts of interest among the included studies. Cochrane Handbook for Systematic Reviews of Interventions version 6.1 (updated September 2020). Cochrane 2020. In: Higgins J, Thomas J, Chandler JC, M, Li T, Page M, Welch V, eds. *Cochrane Handbook for Systematic Reviews of Interventions version 63 (updated February 2022)*. Cochrane; 2022. www.training.cochrane.org/handbook
15. Mokkink LB, De Vet HC, Prinsen CA, Patrick DL, Alonso J, Bouter LM, Terwee CB. COSMIN risk of bias checklist for systematic reviews of patient-reported outcome measures. *Quality of Life Research*. 2018;27:1171-1179.
16. Beaton DE, Maxwell LJ, Shea BJ, et al. Instrument selection using the OMERACT filter 2.1: the OMERACT methodology. *The Journal of rheumatology*. 2019;46(8):1028-1035.
17. Lewis CC, Mettert KD, Stanick CF, Halko HM, Nolen EA, Powell BJ, Weiner BJ. The psychometric and pragmatic evidence rating scale (PAPERS) for measure development and evaluation. *Implementation research and practice*. 2021;2:26334895211037391.

18. Elsman EB, Mokkink LB, Langendoen-Gort M, Rutters F, Beulens J, Elders PJ, Terwee CB. Systematic review on the measurement properties of diabetes-specific patient-reported outcome measures (PROMs) for measuring physical functioning in people with type 2 diabetes. *BMJ Open Diabetes Research and Care*. 2022;10(3):e002729.
19. Terwee CB, Elders PJ, Langendoen-Gort M, et al. Content Validity of Patient-Reported Outcome Measures Developed for Assessing Health-Related Quality of Life in People with Type 2 Diabetes Mellitus: a Systematic Review. *Current Diabetes Reports*. 2022;22(9):405-421.
20. Melissant HC, Neijenhuijs KI, Jansen F, et al. A systematic review of the measurement properties of the Body Image Scale (BIS) in cancer patients. *Supportive Care in Cancer*. 2018;26:1715-1726.
21. Beller EM, Glasziou PP, Altman DG, et al. PRISMA for abstracts: reporting systematic reviews in journal and conference abstracts. *PLoS medicine*. 2013;10(4):e1001419.
22. Uijen AA, Heinst CW, Schellevis FG, van den Bosch WJ, van de Laar FA, Terwee CB, Schers HJ. Measurement properties of questionnaires measuring continuity of care: a systematic review. 2012;
23. Booth A, Clarke M, Ghersi D, Moher D, Petticrew M, Stewart L. Establishing a minimum dataset for prospective registration of systematic reviews: an international consultation. *PLoS One*. 2011;6(11):e27319.
24. Essiet IA, Lander NJ, Salmon J, Duncan MJ, Eyre EL, Ma J, Barnett LM. A systematic review of tools designed for teacher proxy-report of children's physical literacy or constituting elements. *International Journal of Behavioral Nutrition and Physical Activity*. 2021;18(1):1-48.
25. Smith TO, Harvey K. Psychometric properties of pain measurements for people living with dementia: a COSMIN systematic review. *European Geriatric Medicine*. 2022;13(5):1029-1045.
26. Thomas J, Kneale D, McKenzie JE, Brennan SE, Bhaumik S. Chapter 2: Determining the scope of the review and the questions it will address. In: Higgins J, Thomas J, Chandler J, Cumpston, M, Li T, Page M, Welch V, eds. *Cochrane Handbook for Systematic Reviews of Interventions version 63 (updated February 2022)*. 2022. www.training.cochrane.org/handbook
27. Piontek K, Donhauser T, Kann G, Fechtner M, Apfelbacher C, Gabes M. Patient-reported outcome measures for uncomplicated urinary tract infections in women: a systematic review. *Quality of Life Research*. 2023:1-17.
28. Ratter J, Pellekooren S, Wiertsema S, et al. Content validity and measurement properties of the Lower Extremity Functional Scale in patients with fractures of the lower extremities: a systematic review. *Journal of Patient-Reported Outcomes*. 2022;6(1):1-14.
29. Sabah SA, Hedge EA, Abram SG, Alvand A, Price AJ, Hopewell S. Patient-reported outcome measures following revision knee replacement: a review of PROM instrument utilisation and measurement properties using the COSMIN checklist. *BMJ open*. 2021;11(10):e046169.
30. Halvorsen MB, Helverschou SB, Axelsdottir B, Brøndbo PH, Martinussen M. General measurement tools for assessing mental health problems among children and adolescents with an intellectual disability: a systematic review. *Journal of Autism and Developmental Disorders*. 2023;53(1):132-204.
31. Wen H, Yang Z, Zhu Z, Han S, Zhang L, Hu Y. Psychometric properties of self-reported measures of health-related quality of life in people living with HIV: a systematic review. *Health and Quality of Life Outcomes*. 2022;20:1-43.
32. Fan Y, Shu X, Leung KCM, Lo ECM. Patient-reported outcome measures for masticatory function in adults: a systematic review. *BMC oral health*. 2021;21:1-17.
33. Prill R, Walter M, Królikowska A, Becker R. A systematic review of diagnostic accuracy and clinical applications of wearable movement sensors for knee joint rehabilitation. *Sensors*. 2021;21(24):8221.
34. He Q, Luo J, Chen J, Yang J, Yao C, Xu C, Tao Q. The validity and reliability of quality of life questionnaires in patients with ankylosing spondylitis and non-radiographic axial spondyloarthritis: a systematic review and meta-analysis. *Health and Quality of Life Outcomes*. 2022;20(1):116.

35. Kroon FP, van der Heijde D, Maxwell LJ, et al. Core outcome measurement instrument selection for physical function in hand osteoarthritis using the OMERACT Filter 2.1 process. Elsevier; 2021:1311-1319.
36. Kalle J, Saris TF, Sierevelt IN, Eygendaal D, van Bergen CJ. Quality of patient-and proxy-reported outcomes for children with impairment of the upper extremity: a systematic review using the COSMIN methodology. *Journal of Patient-Reported Outcomes*. 2022;6(1):1-17.
37. Abma IL, Butje BJ, Ten Klooster PM, van der Wees PJ. Measurement properties of the Dutch–Flemish patient-reported outcomes measurement information system (PROMIS) physical function item bank and instruments: a systematic review. *Health and quality of life outcomes*. 2021;19(1):1-22.
38. Everaars B, Weening-Verbree LF, Jerković-Ćosić K, Schoonmade L, Bleijenberg N, de Wit NJ, van der Heijden GJ. Measurement properties of oral health assessments for non-dental healthcare professionals in older people: a systematic review. *BMC geriatrics*. 2020;20(1):1-18.
39. Oude Voshaar MA, ten Klooster PM, Taal E, van de Laar MA. Measurement properties of physical function scales validated for use in patients with rheumatoid arthritis: a systematic review of the literature. *Health and quality of life outcomes*. 2011;9(1):1-13.
40. McGhie-Fraser B, Lucassen P, Ballering A, Abma I, Brouwers E, Van Dulmen S, Hartman TO. Persistent somatic symptom related stigmatisation by healthcare professionals: A systematic review of questionnaire measurement instruments. *Journal of Psychosomatic Research*. 2023:111161.
41. Carlton J, Powell PA. Measuring carer quality of life in Duchenne muscular dystrophy: a systematic review of the reliability and validity of self-report instruments using COSMIN. *Health and Quality of Life Outcomes*. 2022;20(1):1-33.
42. Schünemann H, Higgins J, Vist G, Glasziou P, Akl E, Skoetz N, Guyatt G. Chapter 14: Completing 'Summary of findings' tables and grading the certainty of the evidence. In: Higgins J, Thomas J, Chandler JC, M , Li T, Page M, Welch V, eds. *Cochrane Handbook for Systematic Reviews of Interventions version 63 (updated February 2022)*. Cochrane; 2022. www.training.cochrane.org/handbook
43. Pizzinato A, Liguoro I, Pusiol A, Cogo P, Palese A, Vidal E. Detection and assessment of postoperative pain in children with cognitive impairment: A systematic literature review and meta-analysis. *European Journal of Pain*. 2022;26(5):965-979.
44. Aiyegbusi OL, Kyte D, Cockwell P, et al. Measurement properties of patient-reported outcome measures (PROMs) used in adult patients with chronic kidney disease: a systematic review. *PLoS One*. 2017;12(6):e0179733.
45. Brekke M, Berg RC, Amro A, Glavin K, Haugland T. Quality of Life instruments and their psychometric properties for use in parents during pregnancy and the postpartum period: a systematic scoping review. *Health and Quality of Life Outcomes*. 2022;20(1):1-19.
46. Elgueta-Cancino E, Rice K, Abichandani D, Falla D. Measurement properties of smartphone applications for the measurement of neck range of motion: a systematic review and meta analyses. *BMC Musculoskeletal Disorders*. 2022;23(1):1-17.
47. Stallwood E, Elsman EBM, Monsour A, Baba A, Butcher NJ, Offringa M. Measurement properties of pediatric PROMIS questionnaires for overall pediatric health: a systematic review. Abstract submitted for the 9th Annual PROMIS International Conference; 2023:
48. Tancock C. In a nutshell: how to write a lay summary. Elsevier Connect; 2018.
49. McIlwain C, Santesso N, Simi S, et al. Standards for the reporting of Plain Language Summaries in new Cochrane Intervention Reviews (PLEACS). 2014;
50. Harris A, Hay P, Touyz S. Psychometric properties of instruments assessing exercise in patients with eating disorders: a systematic review. *Journal of Eating Disorders*. 2020;8:1-14.
51. Sender D, Clark J, Hoffmann TC. Analysis of articles directly related to randomized trials finds poor protocol availability and inconsistent linking of articles. *Journal of Clinical Epidemiology*. 2020;124:69-74.

52. Kipfer S, Pihet S. Reliability, validity and relevance of needs assessment instruments for informal dementia caregivers: a psychometric systematic review. *JBI evidence synthesis*. 2020;18(4):704.
53. Shamseer L, Moher D, Clarke M, et al. Preferred reporting items for systematic review and meta-analysis protocols (PRISMA-P) 2015: elaboration and explanation. *Bmj*. 2015;349
54. Koensgen N, Rombey T, Allers K, Mathes T, Hoffmann F, Pieper D. Comparison of non-Cochrane systematic reviews and their published protocols: differences occurred frequently but were seldom explained. *Journal of clinical epidemiology*. 2019;110:34-41.
55. Pieper D, Allers K, Mathes T, Hoffmann F, Klerings I, Rombey T, Nussbaumer-Streit B. Comparison of protocols and registry entries to published reports for systematic reviews. *The Cochrane Database of Systematic Reviews*. 2020;2020(2)
56. Lundh A, Lexchin J, Mintzes B, Schroll JB, Bero L. Industry sponsorship and research outcome. *Cochrane database of systematic reviews*. 2017;(2)
57. Taichman DB, Backus J, Baethge C, et al. A disclosure form for work submitted to medical journals: a proposal from the International Committee of Medical Journal Editors. *The Lancet*. 2022;399(10330):e15-e16.
58. Haddaway NR. Open Synthesis: on the need for evidence synthesis to embrace Open Science. *Environmental evidence*. 2018;7(1):1-5.
59. Goldacre B, Morton CE, DeVito NJ. Why researchers should share their analytic code. British Medical Journal Publishing Group; 2019.
60. Saldanha IJ, Smith BT, Ntzani E, Jap J, Balk EM, Lau J. The Systematic Review Data Repository (SRDR): descriptive characteristics of publicly available data and opportunities for research. *Systematic reviews*. 2019;8(1):1-12.
61. Wilkinson MD, Dumontier M, Aalbersberg IJ, et al. The FAIR Guiding Principles for scientific data management and stewardship. *Scientific data*. 2016;3(1):1-9.
62. de Morton NA, Berlowitz DJ, Keating JL. A systematic review of mobility instruments and their measurement properties for older acute medical patients. *Health and Quality of Life Outcomes*. 2008;6(1):1-15.
63. Huang F-F, Yang Q, Wang A-n, Zhang J-P. Psychometric properties and performance of existing self-efficacy instruments in cancer populations: a systematic review. *Health and Quality of Life Outcomes*. 2018;16(1):1-12.
64. Boers M, Beaton DE, Shea BJ, et al. OMERACT filter 2.1: elaboration of the conceptual framework for outcome measurement in health intervention studies. *The Journal of rheumatology*. 2019;46(8):1021-1027.
65. Maxwell LJ, Beaton DE, Boers M, et al. The evolution of instrument selection for inclusion in core outcome sets at OMERACT: Filter 2.2. Elsevier; 2021:1320-1330.
66. Aromataris E, Munn Z. *JBI Manual for Evidence Synthesis*. JBI; 2020. https://synthesismanual.jbi.global
67. IOM. Finding what works in health care: standards for systematic reviews. The National Academies Press Washington, DC; 2011.
68. Tomacheuski RM, Monteiro BP, Evangelista MC, Luna SPL, Steagall PV. Measurement properties of pain scoring instruments in farm animals: A systematic review using the COSMIN checklist. *PloS one*. 2023;18(1):e0280830.
69. Lefebvre C, Glanville J, Briscoe S, et al. Chapter 4: Searching for and selecting studies. . In: Higgins J, Thomas J, Chandler J, Cumpston M, Li T, Page M, Welch V, eds. *Cochrane Handbook for Systematic Reviews of Interventions version 63 (updated February 2022)*. Cochrane; 2022. www.training.cochrane.org/handbook
70. Rethlefsen ML, Kirtley S, Waffenschmidt S, Ayala AP, Moher D, Page MJ, Koffel JB. PRISMA-S: an extension to the PRISMA statement for reporting literature searches in systematic reviews. *Systematic reviews*. 2021;10(1):1-19.

71. Faggion Jr CM, Huivin R, Aranda L, Pandis N, Alarcon M. The search and selection for primary studies in systematic reviews published in dental journals indexed in MEDLINE was not fully reproducible. *Journal of clinical epidemiology*. 2018;98:53-61.
72. Spry C, Mierzwinski-Urban M. The impact of the peer review of literature search strategies in support of rapid review reports. *Research synthesis methods*. 2018;9(4):521-526.
73. Terwee CB, Jansma EP, Riphagen II, de Vet HC. Development of a methodological PubMed search filter for finding studies on measurement properties of measurement instruments. *Quality of Life Research*. 2009;18:1115-1123.
74. Mackintosh A, Casañas i Comabella C, Hadi M, Gibbons E, Fitzpatrick R, Roberts N. PROM GROUP CONSTRUCT & INSTRUMENT TYPE FILTERS. Accessed 2023, May. https://cosmin.nl/wp-content/uploads/prom-search-filter-oxford-2010.pdf
75. Glanville J. Text mining for information specialists. *Systematic Searching: Practical ideas for improving results Facet Publishing*. 2019:147-170.
76. Stansfield C, O'Mara-Eves A, Thomas J. Text mining for search term development in systematic reviewing: A discussion of some methods and challenges. *Research synthesis methods*. 2017;8(3):355-365.
77. Clark J, Glasziou P, Del Mar C, Bannach-Brown A, Stehlik P, Scott AM. A full systematic review was completed in 2 weeks using automation tools: a case study. *Journal of clinical epidemiology*. 2020;121:81-90.
78. McGowan J, Sampson M, Salzwedel DM, Cogo E, Foerster V, Lefebvre C. PRESS peer review of electronic search strategies: 2015 guideline statement. *Journal of clinical epidemiology*. 2016;75:40-46.
79. Wang Z, Nayfeh T, Tetzlaff J, O'Blenis P, Murad MH. Error rates of human reviewers during abstract screening in systematic reviews. *PloS one*. 2020;15(1):e0227742.
80. Waffenschmidt S, Knelangen M, Sieben W, Bühn S, Pieper D. Single screening versus conventional double screening for study selection in systematic reviews: a methodological systematic review. *BMC medical research methodology*. 2019;19(1):1-9.
81. Gartlehner G, Affengruber L, Titscher V, Noel-Storr A, Dooley G, Ballarini N, König F. Single-reviewer abstract screening missed 13 percent of relevant studies: a crowd-based, randomized controlled trial. *Journal of clinical epidemiology*. 2020;121:20-28.
82. Shemilt I, Khan N, Park S, Thomas J. Use of cost-effectiveness analysis to compare the efficiency of study identification methods in systematic reviews. *Systematic reviews*. 2016;5:1-13.
83. Robson RC, Hwee J, Thomas SM, Rios P, Page MJ, Tricco AC. Few studies exist examining methods for selecting studies, abstracting data, and appraising quality in a systematic review. *Journal of clinical epidemiology*. 2019;106:121-135.
84. Li T, Saldanha IJ, Jap J, et al. A randomized trial provided new evidence on the accuracy and efficiency of traditional vs. electronically annotated abstraction approaches in systematic reviews. *Journal of Clinical Epidemiology*. 2019;115:77-89.
85. JY E, Saldanha IJ, Canner J, Schmid CH, Le JT, Li T. Adjudication rather than experience of data abstraction matters more in reducing errors in abstracting data in systematic reviews. *Research synthesis methods*. 2020;11(3):354-362.
86. Li T, Higgins JP, Deeks JJ. Chapter 5: Collecting data. In: Higgins J, Thomas J, Chandler J, Cumpston M, Li T, Page M, Welch V, eds. *Cochrane Handbook for Systematic Reviews of Interventions version 63 (updated February 2022)*. Cochrane; 2019. www.training.cochrane.org/handbook
87. Jonnalagadda SR, Goyal P, Huffman MD. Automating data extraction in systematic reviews: a systematic review. *Systematic reviews*. 2015;4(1):1-16.
88. Marshall IJ, Wallace BC. Toward systematic review automation: a practical guide to using machine learning tools in research synthesis. *Systematic reviews*. 2019;8:1-10.

89. Jackson JL, Kuriyama A, Anton A, et al. The accuracy of Google Translate for abstracting data from non–English-language trials for systematic reviews. *Annals of internal medicine*. 2019;171(9):677-679.
90. Kadic AJ, Vucic K, Dosenovic S, Sapunar D, Puljak L. Extracting data from figures with software was faster, with higher interrater reliability than manual extraction. *Journal of clinical epidemiology*. 2016;74:119-123.
91. Mayo-Wilson E, Li T, Fusco N, Dickersin K, investigators M. Practical guidance for using multiple data sources in systematic reviews and meta-analyses (with examples from the MUDS study). *Research synthesis methods*. 2018;9(1):2-12.
92. Lorente S, Viladrich C, Vives J, Losilla J-M. Tools to assess the measurement properties of quality of life instruments: a meta-review. *BMJ open*. 2020;10(8):e036038.
93. Devji T, Carrasco-Labra A, Qasim A, et al. Evaluating the credibility of anchor based estimates of minimal important differences for patient reported outcomes: instrument development and reliability study. *Bmj*. 2020;369
94. Wang Y, Devji T, Carrasco-Labra A, et al. An extension minimal important difference credibility item addressing construct proximity is a reliable alternative to the correlation item. *Journal of Clinical Epidemiology*. 2023;157:46-52.
95. Isa F, Turner GM, Kaur G, et al. Patient-reported outcome measures used in patients with primary sclerosing cholangitis: a systematic review. *Health and quality of life outcomes*. 2018;16:1-18.
96. Jakobsson M, Gutke A, Mokkink LB, Smeets R, Lundberg M. Level of evidence for reliability, validity, and responsiveness of physical capacity tasks designed to assess functioning in patients with low back pain: a systematic review using the COSMIN standards. *Physical therapy*. 2019;99(4):457-477.
97. Viechtbauer W. Conducting meta-analyses in R with the metafor package. *Journal of statistical software*. 2010;36(3):1-48.
98. Deeks J, Higgins J, Altman D. Chapter 10: analysing data and undertaking meta-analyses. In: Higgins J, Thomas J, Chandler J, Cumpston M, Li T, Page M, Welch V, eds. *Cochrane Handbook for Systematic Reviews of Interventions version 63 (updated February 2022)*. Cochrane; 2022. www.training.cochrane.org/handbook
99. Higgins JP, Thompson SG, Deeks JJ, Altman DG. Measuring inconsistency in meta-analyses. *Bmj*. 2003;327(7414):557-560.
100. Riley RD, Higgins JP, Deeks JJ. Interpretation of random effects meta-analyses. *Bmj*. 2011;342
101. Zeng Z, Liu Y, Hu X, Tang M, Wang L. Validity and reliability of inertial measurement units on lower extremity kinematics during running: A systematic review and meta-analysis. *Sports Medicine-Open*. 2022;8(1):86.
102. Strong A, Arumugam A, Tengman E, Röijezon U, Häger CK. Properties of tests for knee joint threshold to detect passive motion following anterior cruciate ligament injury: a systematic review and meta-analysis. *Journal of Orthopaedic Surgery and Research*. 2022;17(1):1-15.
103. Guyatt GH, Oxman AD, Kunz R, et al. GRADE guidelines 6. Rating the quality of evidence—imprecision. *Journal of clinical epidemiology*. 2011;64(12):1283-1293.
104. Sampson M, Tetzlaff J, Urquhart C. Precision of healthcare systematic review searches in a cross-sectional sample. *Research synthesis methods*. 2011;2(2):119-125.
105. Haddaway NR, Westgate MJ. Predicting the time needed for environmental systematic reviews and systematic maps. *Conservation Biology*. 2019;33(2):434-443.
106. Baamer RM, Iqbal A, Lobo DN, Knaggs RD, Levy NA, Toh LS. The utility of unidimensional and functional pain assessment tools in adult postoperative patients: a systematic review. *British journal of anaesthesia*. 2022;
107. Mihaljevic AL, Doerr-Harim C, Kalkum E, Strunk G. Measuring patient centeredness with German language Patient-Reported Experience Measures (PREM)–A systematic review and qualitative analysis according to COSMIN. *Plos one*. 2022;17(11):e0264045.

108. Ghaderi C, Esmaeili R, Ebadi A, Amiri MR. Measuring situation awareness in health care providers: a systematic review of measurement properties using COSMIN methodology. *Systematic Reviews*. 2023;12(1):1-15.
109. McKenzie J, Brennan S, Ryan R, Thomson H, Johnston R. Chapter 9: summarizing study characteristics and preparing for synthesis. In: Higgins J, Thomas J, Chandler J, Cumpston M, Li T, Page M, Welch V, eds. *Cochrane Handbook for Systematic Reviews of Interventions version 63 (updated February 2022)*. Cochrane; 2022. www.training.cochrane.org/handbook
110. Shah K, Egan G, Huan LN, Kirkham J, Reid E, Tejani AM. Outcome reporting bias in Cochrane systematic reviews: a cross-sectional analysis. *BMJ open*. 2020;10(3):e032497.
111. Campbell M, McKenzie JE, Sowden A, et al. Synthesis without meta-analysis (SWiM) in systematic reviews: reporting guideline. *bmj*. 2020;368
112. Guyatt G, Oxman AD, Akl EA, et al. GRADE guidelines: 1. Introduction—GRADE evidence profiles and summary of findings tables. *Journal of clinical epidemiology*. 2011;64(4):383-394.
113. Mokkink LB, Prinsen CA, Patrick DL, Alonso J, Bouter LM, De Vet HC, Terwee CB. *COSMIN methodology for systematic reviews of Patient-Reported Outcome Measures (PROMs) - user manual. Version 1.0, February 2018.* . 2018. https://www.cosmin.nl/wp-content/uploads/COSMIN-syst-review-for-PROMs-manual_version-1_feb-2018-1.pdf
114. Santesso N, Carrasco-Labra A, Langendam M, et al. Improving GRADE evidence tables part 3: detailed guidance for explanatory footnotes supports creating and understanding GRADE certainty in the evidence judgments. *Journal of clinical epidemiology*. 2016;74:28-39.
115. Bobos P, Pouliopoulou DV, Harriss A, Sadi J, Rushton A, MacDermid JC. A systematic review and meta-analysis of measurement properties of objective structured clinical examinations used in physical therapy licensure and a structured review of licensure practices in countries with well-developed regulation systems. *PloS one*. 2021;16(8):e0255696.
